# Supplementary material for: The effect of type 2 diabetes genetic predisposition on non-cardiovascular comorbidities
Source: Nat Commun. 2025 Oct 10;16:9042. doi: 10.1038/s41467-025-64927-5 (PMC12514310; doi:10.1038/s41467-025-64927-5)
Supplement: Supplementary file 1 — Supplementary Information [file 41467_2025_64927_MOESM1_ESM.pdf]

Supplementary Information

The effect of type 2 diabetes genetic predisposition on non-cardiovascular comorbidities

Arruda et al.

Disease	Study	Ancestry	OR	lci_OR	uci_OR
Carpal tunnel syndrome	PMID: 34367246	EUR	1.17	1.07	1.29
Carpal tunnel syndrome	PMID: 39560586	EUR	1.1	1.05	1.16
Osteoporosis	PMID: 37268885	EUR	0.9985	0.9974	0.9995
Osteoporosis	PMID: 37306802	EAS	0.92	0.86	0.99
Glaucoma	PMID: 35622353	EUR	1.07	1.01	1.14
Glaucoma	PMID: 38672220	EUR	1.05	1	1.1
Glaucoma	PMID: 38075077	EUR	1.08	1.02	1.13
Glaucoma	PMID: 36677034	EUR	1.11	1.06	1.16
Cataracts	PMID: 35013517	EUR	1.06	1.03	1.09
Polycystic ovary syndrome	PMID: 35771237	EUR	1.15	1.06	1.25
Osteoarthritis	PMID: 37433298	EUR	1.01	0.98	1.03
Osteoarthritis	PMID: 31099188	EUR	1.01	0.99	1.03
Erectile dysfunction	PMID: 34842357	EUR	1.14	1.08	1.21
Erectile dysfunction	PMID: 38152129	EUR	1.19	1.08	1.3
Erectile dysfunction	PMID: 40463815	EUR	1.1	1.04	1.16
Erectile dysfunction	PMID: 37236543	EUR	1.15	1.09	1.21
Attention deficit disorder	PMID: 36937092	EUR	1.03	1.01	1.04
Attention deficit disorder	PMID: 36259254	EUR	1.09	1.04	1.14

Supplementary Table 1: Overview of results of previous two-sample Mendelian randomization studies investigating the causal effect of type 2 diabetes liability on comorbidity risk. (OR = odds ratio; lci_OR = lower bound of 95% confidence interval of OR; uci_OR = upper bound of 95% confidence interval of OR).

Genetic ancestry group	Number of cases	Number of controls
AFR	50,251	103,909
EAS	88,109	339,395
EUR	242,283	1,569,734
AMR	29,375	59,368
SAS	16,832	33,767

Supplementary Table 2: Sample size of each genetic similarity group type 2 diabetes GWAS meta-analysis from T2DGGI consortium.

	All T2DGGI risk variants	One variant per T2DGGI locus	Cluster-specific clumping				
			**AFR**	**AMR**	**EAS**	**EUR**	**SAS**
**All**	1289	611	29	22	138	315	8
**Beta cell +PI**	91	45	8	10	26	50	5
**Beta cell -PI**	89	45	4	5	28	51	1
**Body fat**	273	110	5	2	34	110	0
**Lipodystrophy**	45	29	2	0	6	35	0
**Liver/lipid metabolism**	3	2	0	0	0	3	0
**Metabolic syndrome**	166	85	6	5	24	89	2
**Obesity**	233	154	3	2	19	123	0
**Residual glycaemic**	389	141	9	5	65	136	0

Supplementary Table 3: Number of type 2 diabetes genetic instrumental variables (IVs) for each mechanistic cluster using different approaches to select IVs employed as sensitivity analysis for the Mendelian randomization analysis. (As per 1000 Genomes Project genetic ancestry groups: AFR = African, AMR = admixed American, EAS = East Asian, EUR = European, SAS = South Asian).

Genetic ancestry group	Sample size
EUR	91,365
AFR	30,538
AMR	25,688
EAS	2,964
SAS	1,481

Supplementary Table 4: Sample size of each genetic similarity group from the All of Us genetic data used to perform the PheWAS. (As per 1000 Genomes Project genetic ancestry groups: AFR = African, AMR = admixed American, EAS = East Asian, EUR = European, SAS = South Asian).

Mendelian randomization assumption	Sensitivity analysis
Relevance assumption	• F-statistic<br>• Steiger-filtered inverse variance weighted (IVW)
Independence assumption	• Multivariable MR with potential confounders/mediators*<br>• Analysis within genetic similarity groups<br>• Exposure and outcome GWAS summary statistics adjusted for population structure with the removal of related individuals
Exclusion-restriction assumption	• MR-Egger intercept test<br>• Heterogeneity assessed by the I^2 statistic<br>• Same direction of effect across different MR methods:<br>o Steiger-filtered IVW<br>o Correlated IVW<br>o MR-Egger<br>o Weighted median<br>o MR-PRESSO

Supplementary Table 5: Overview of the sensitivity analyses performed to assess the validity of the Mendelian randomization (MR) assumptions. * Definition of potential confounders/mediators: cardiometabolic traits used to cluster the type 2 diabetes (T2D) variants that have a potential causal IVW effect on the comorbidity at a false discovery rate (FDR) of 5%.

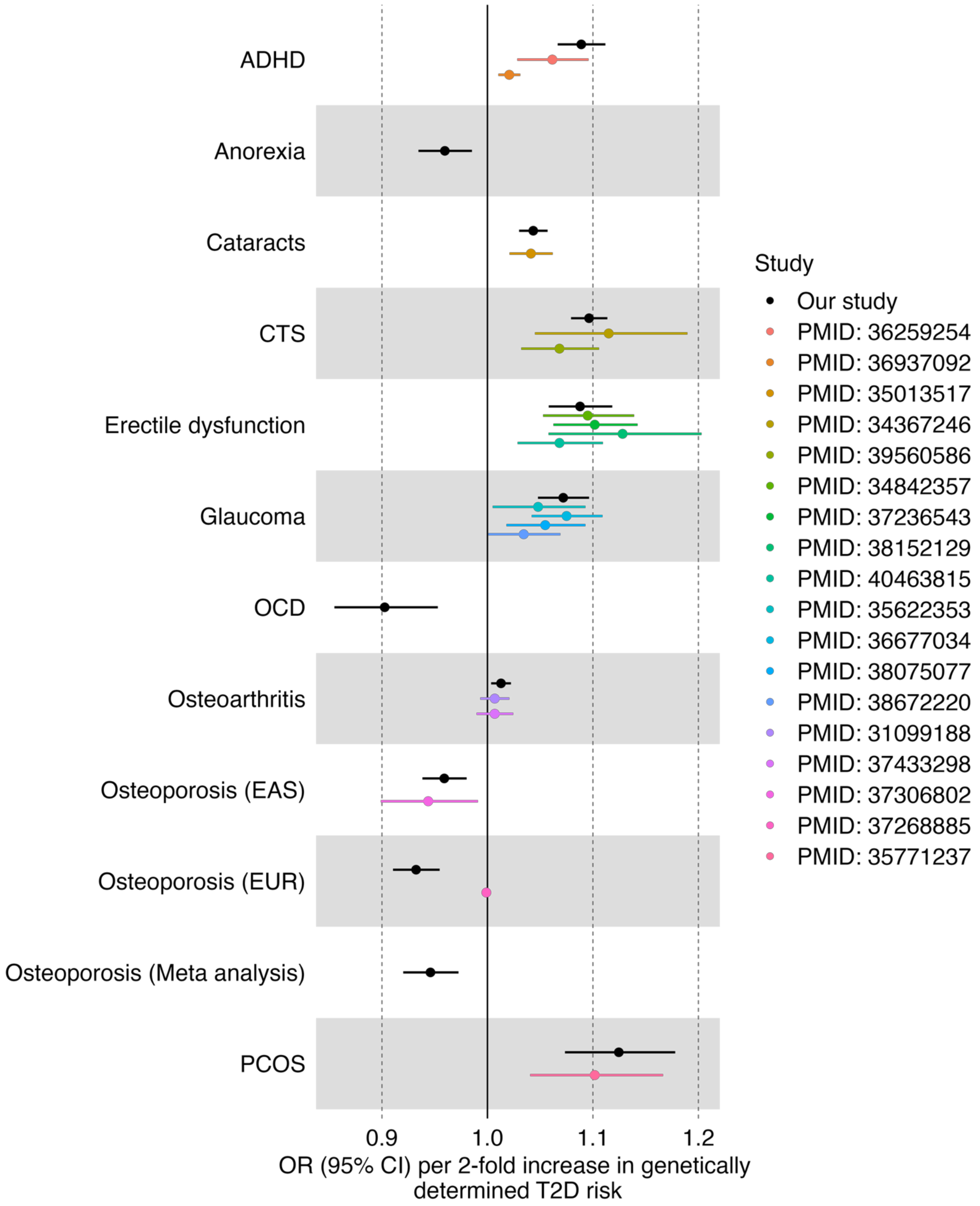

Supplementary Figure 1: Comparison of ours and previous studies results of two-sample Mendelian randomization (MR) analysis of genetic predisposition for type 2 diabetes (T2D) on non-cardiovascular comorbidity risk for the causal relationships. Causal estimates are expressed as the odds ratio (OR) for each comorbidity per doubling (2-fold increase) in genetically determined dichotomous T2D risk. (CI = confidence interval; CTS = Carpal tunnel syndrome; ADHD = attention-deficit/hyperactivity disorder; OCD = obsessive-compulsive disorder; PCOS = polycystic ovary syndrome).

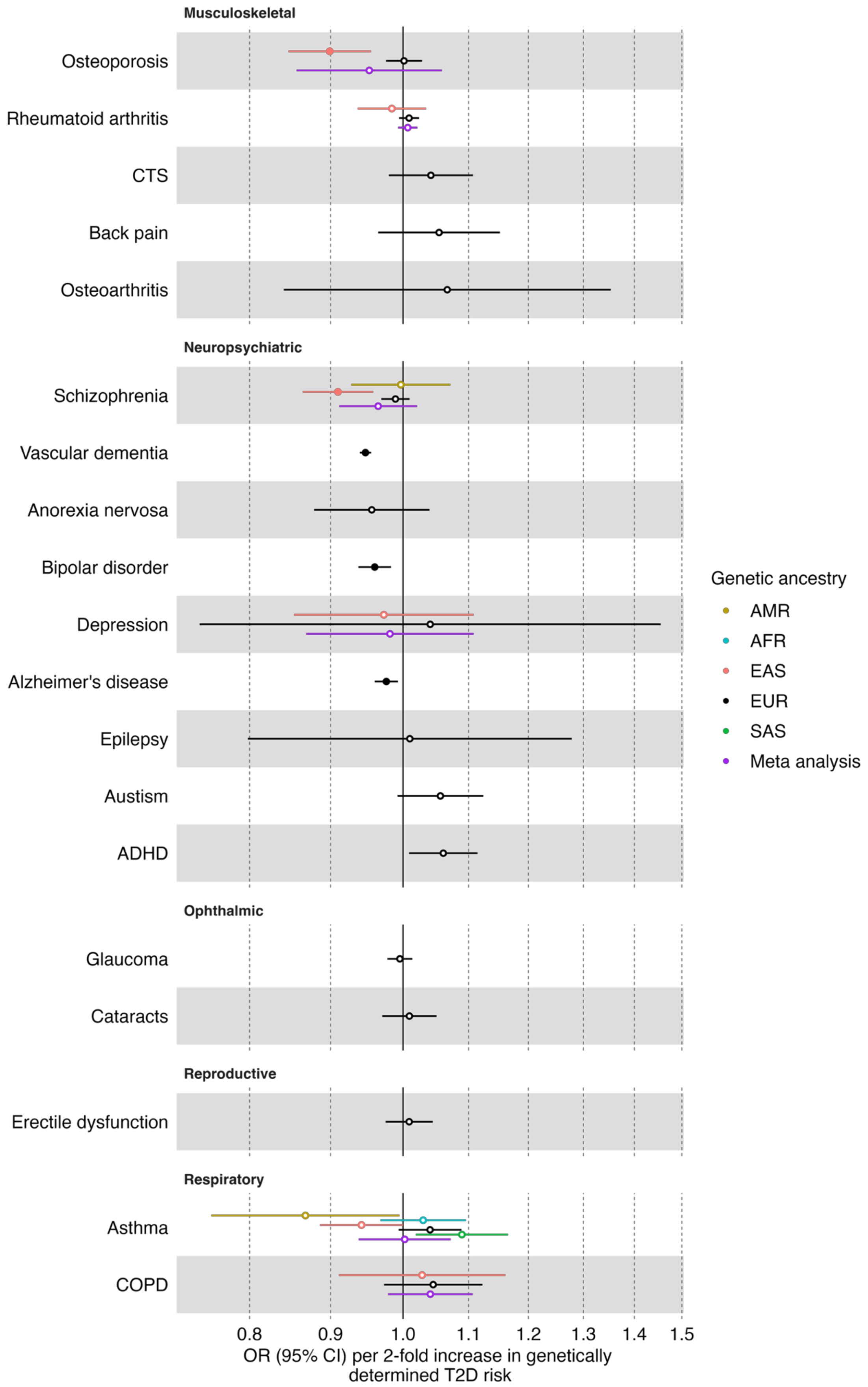

Supplementary Figure 2: Results of the reverse Mendelian randomization (MR) analysis using genetic predisposition for type 2 diabetes (T2D) comorbidities as exposure and T2D as the outcome. Causal estimates are expressed as the odds ratio of comorbidity risk per doubling (2-fold increase) in genetically determined dichotomous T2D risk. Filled circles mark estimates with a false-discovery-rate correction of 5%. No estimate passed the MR sensitivity analyses. The genetic ancestry groups represent individuals genetically similar to Africans (AFR), East Asians (EAS), Europeans (EUR), admixed Americans (AMR) and South Asians (SAS) as defined by the 1000 Genomes Project phase 3. (CI = confidence interval).

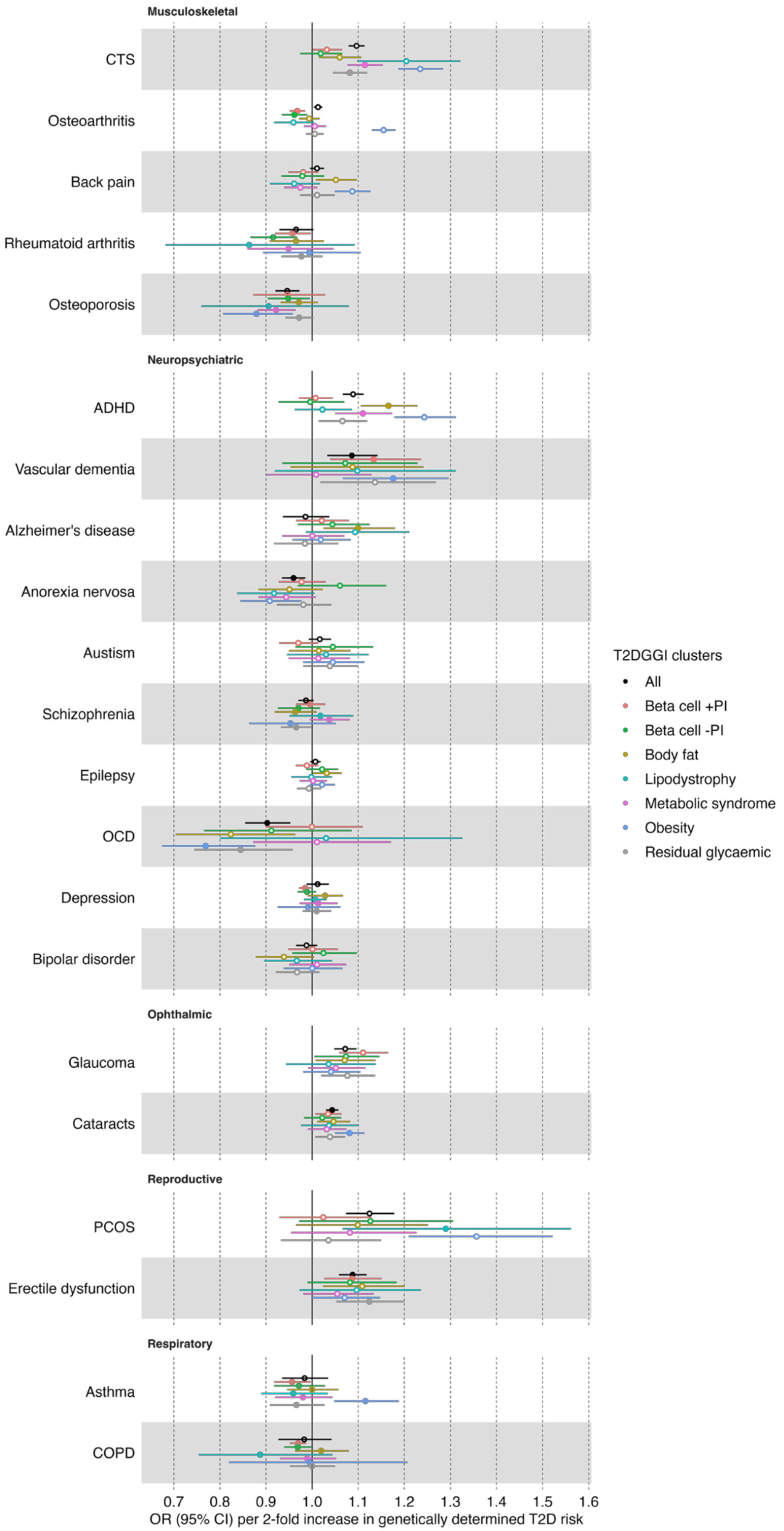

Supplementary Figure 3: Results of cluster-stratified two-sample Mendelian randomization (MR) analysis of genetic predisposition for type 2 diabetes (T2D) on non-cardiovascular comorbidities risk. Causal estimates are expressed as the odds ratio (OR) of each comorbidity per doubling (2-fold increase) in genetically determined dichotomous T2D risk. Filled circles mark estimates with a q-value < 0.05 that passed all sensitivity analyses to assess the validity of the MR assumptions. (T2DGGI = Type 2 Diabetes Global Genomics Initiative; CI = confidence interval; PI = proinsulin; CTS = Carpal tunnel syndrome; ADHD = attention-deficit/hyperactivity disorder; OCD = obsessive-compulsive disorder; PCOS = polycystic ovary syndrome; PCOS = polycystic ovary syndrome; COPD = chronic obstructive pulmonary disease)

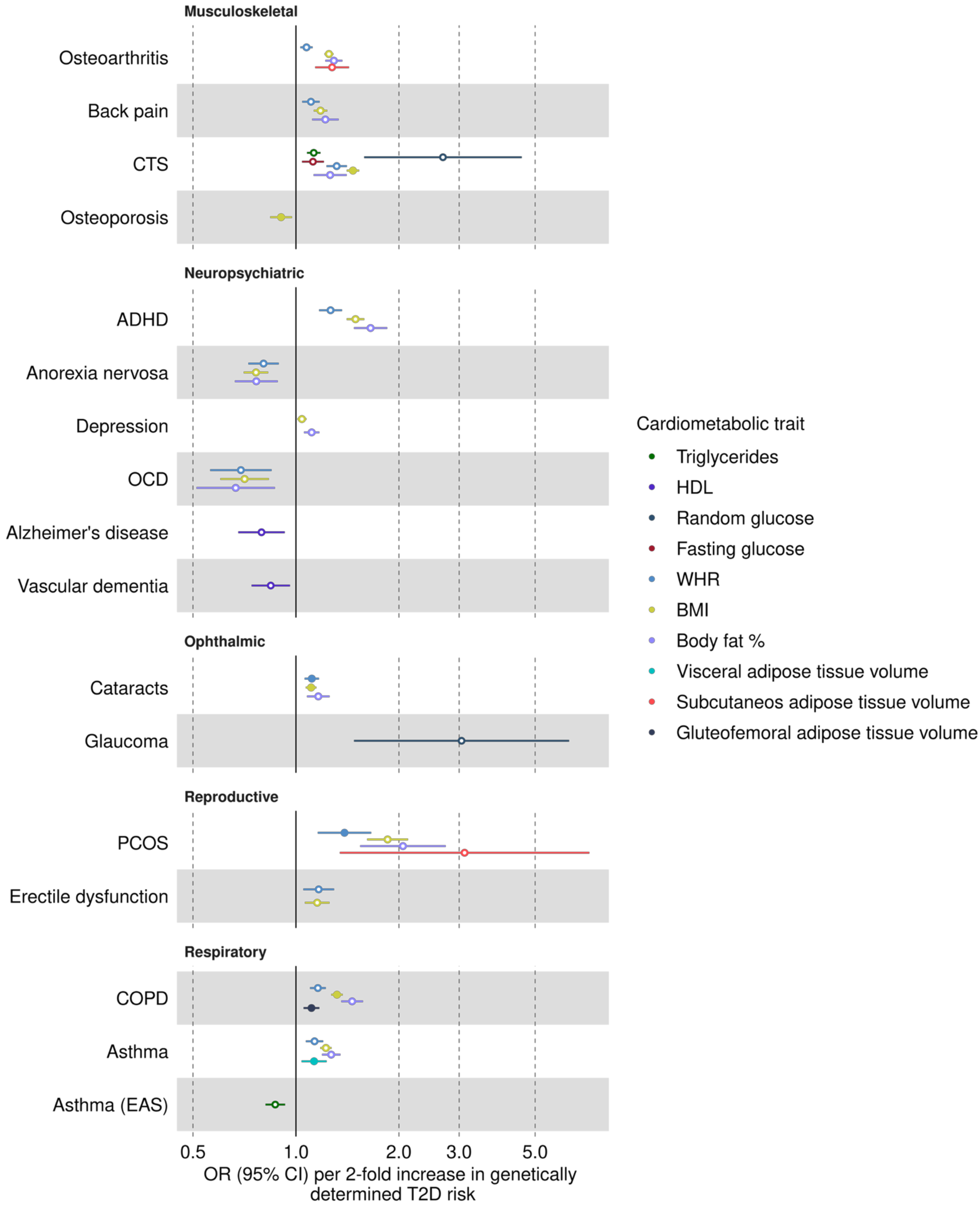

Supplementary Figure 4: Univariable Mendelian randomization (MR) results with genetic predisposition for cardiometabolic traits as exposures and type 2 diabetes (T2D) comorbidities as outcomes. Causal estimates are expressed as the odds ratio of comorbidity risk per doubling (2-fold increase) in genetically determined dichotomous T2D risk. Filled circles denote robust causal estimates that passed sensitivity analysis and false-discovery rate correction at 5%. (CI = confidence interval; PI = proinsulin; CTS = Carpal tunnel syndrome; ADHD = attention-deficit/hyperactivity disorder; OCD = obsessive-compulsive disorder; PCOS = polycystic ovary syndrome; COPD = chronic obstructive pulmonary disease; HDL=high-density lipoprotein cholesterol; WHR = waist-to-hip ratio; BMI=body mass index).

Supplementary Figures 5-28: Forest plots comparing the results of the univariable and multivariable Mendelian randomization (MR) analyses using data from individuals genetically similar to Europeans. Causal estimates are expressed as the odds ratio of comorbidity risk per doubling (2-fold increase) in genetically determined dichotomous T2D risk. (HDL=high-density lipoprotein cholesterol; WHR = waist-to-hip ratio; BMI=body mass index; CI=confidence interval; FDR=false-discovery-rate at 5%).

5

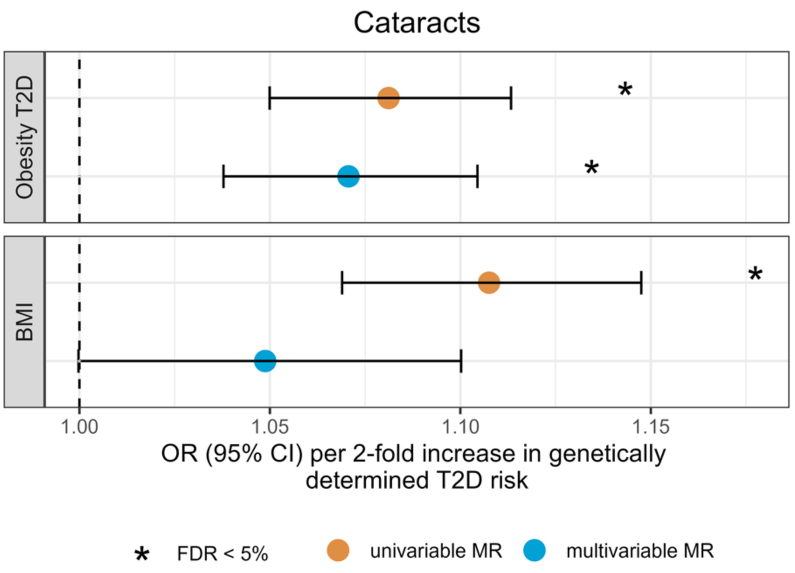
Cataracts
Obesity T2D
BMI
1.00
1.05
1.10
1.15
OR (95% CI) per 2-fold increase in genetically determined T2D risk
* FDR < 5%
univariable MR
multivariable MR

6

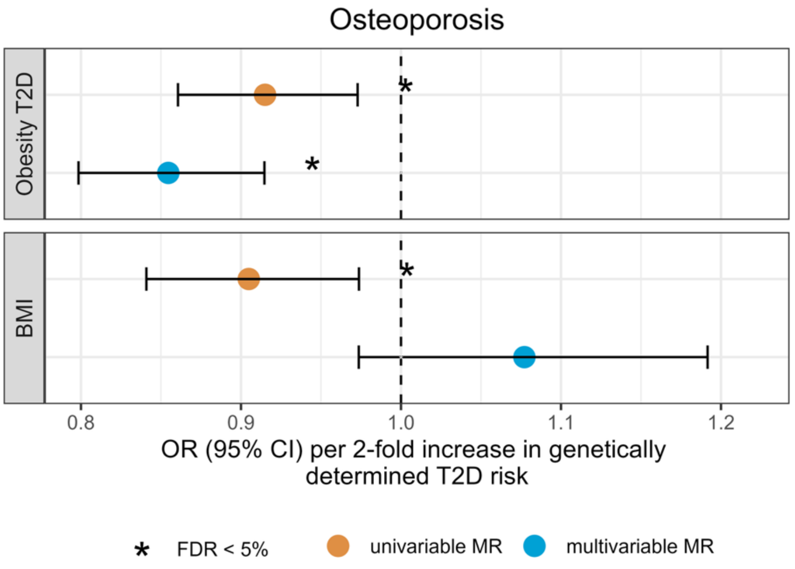
Osteoporosis
Obesity T2D
BMI
0.8
0.9
1.0
1.1
1.2
OR (95% CI) per 2-fold increase in genetically determined T2D risk
* FDR < 5%
univariable MR
multivariable MR

7

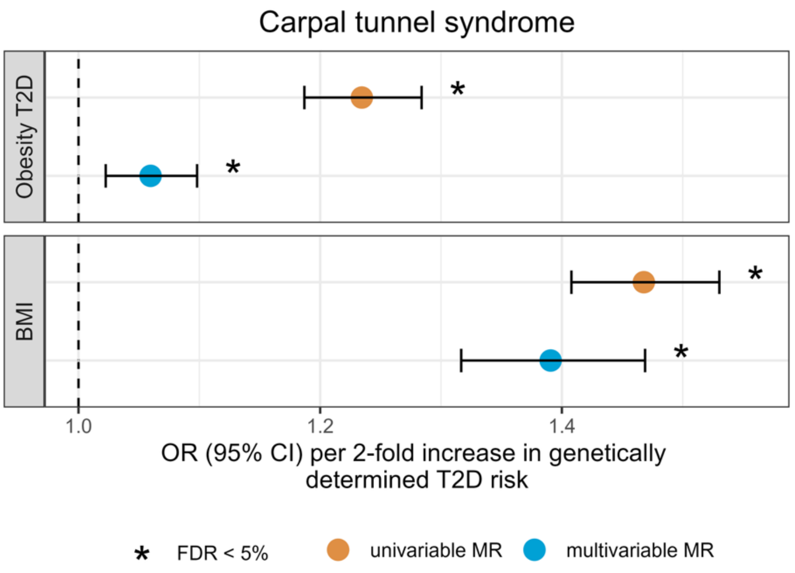
Carpal tunnel syndrome
Obesity T2D
BMI
1.0
1.2
1.4
OR (95% CI) per 2-fold increase in genetically determined T2D risk
* FDR < 5%
univariable MR
multivariable MR

8

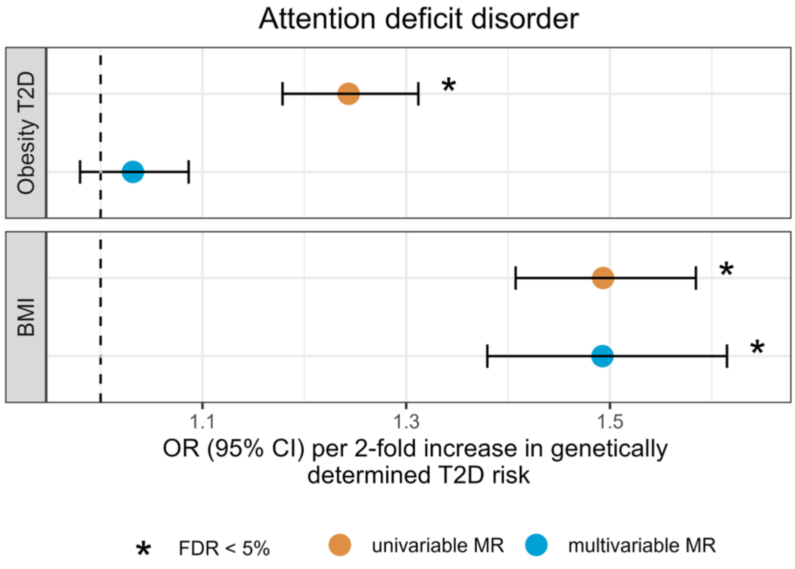
Attention deficit disorder
Obesity T2D
BMI
1.1
1.3
1.5
OR (95% CI) per 2-fold increase in genetically determined T2D risk
* FDR < 5%
univariable MR
multivariable MR

9

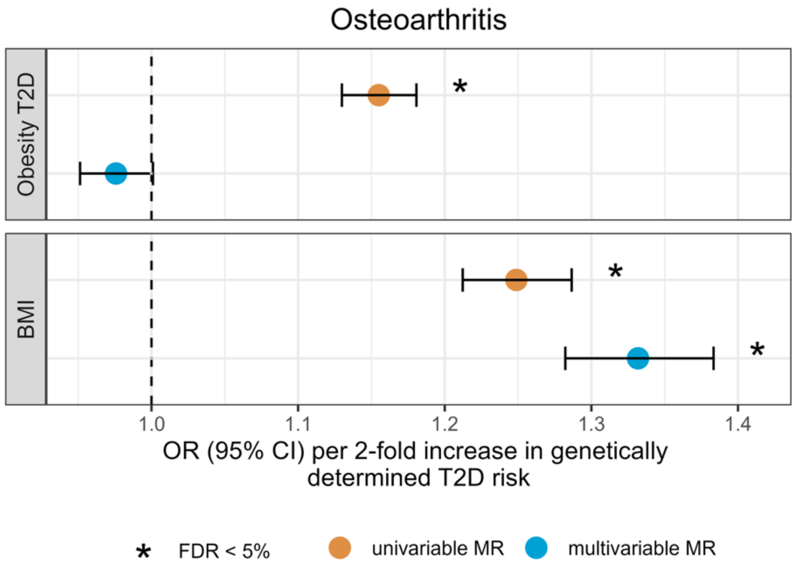
Osteoarthritis
Obesity T2D
BMI
1.0
1.1
1.2
1.3
1.4
OR (95% CI) per 2-fold increase in genetically determined T2D risk
* FDR < 5%
univariable MR
multivariable MR

10

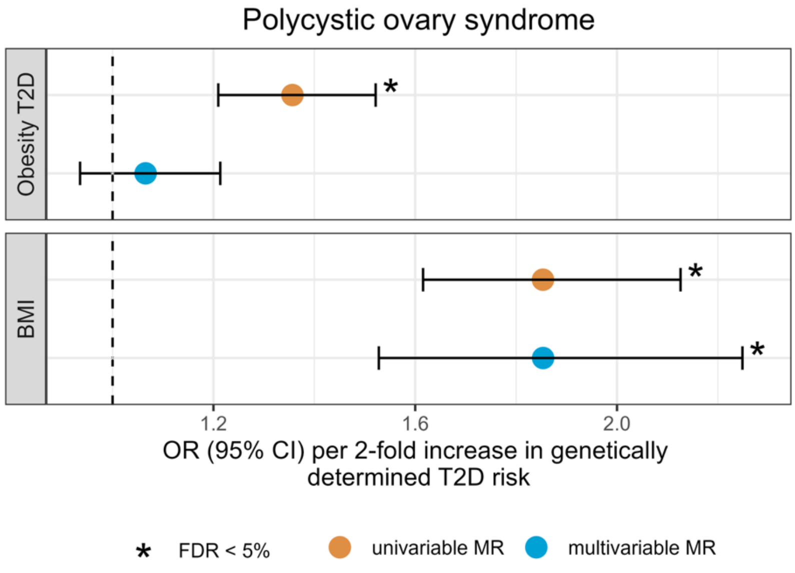
Polycystic ovary syndrome
Obesity T2D
BMI
1.2
1.6
2.0
OR (95% CI) per 2-fold increase in genetically determined T2D risk
* FDR < 5%
univariable MR
multivariable MR

11

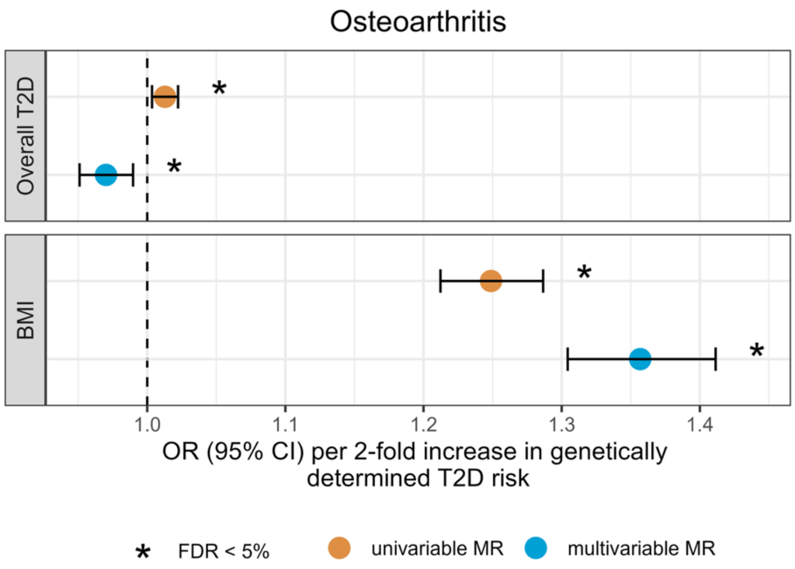
Osteoarthritis
Overall T2D
BMI
*
1.0
1.1
1.2
1.3
1.4
OR (95% CI) per 2-fold increase in genetically determined T2D risk
FDR < 5%
univariable MR
multivariable MR

12

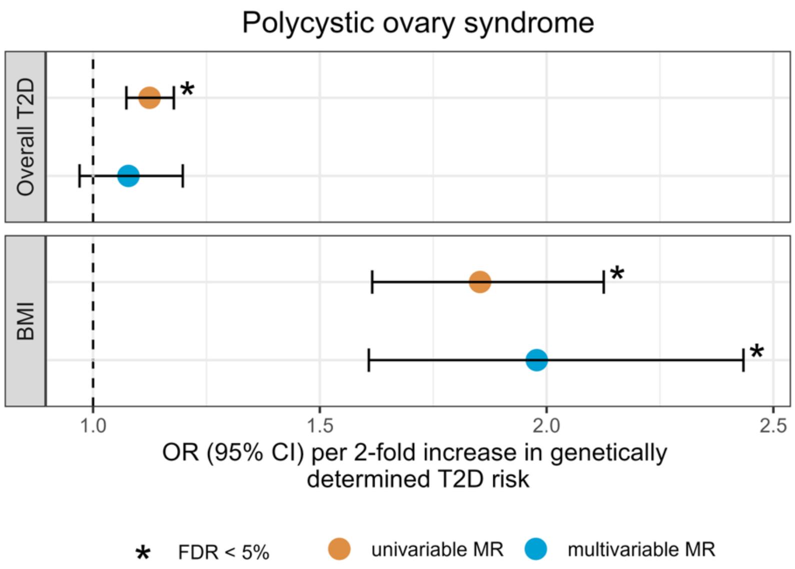
Polycystic ovary syndrome
Overall T2D
BMI
*
1.0
1.5
2.0
2.5
OR (95% CI) per 2-fold increase in genetically determined T2D risk
FDR < 5%
univariable MR
multivariable MR

13

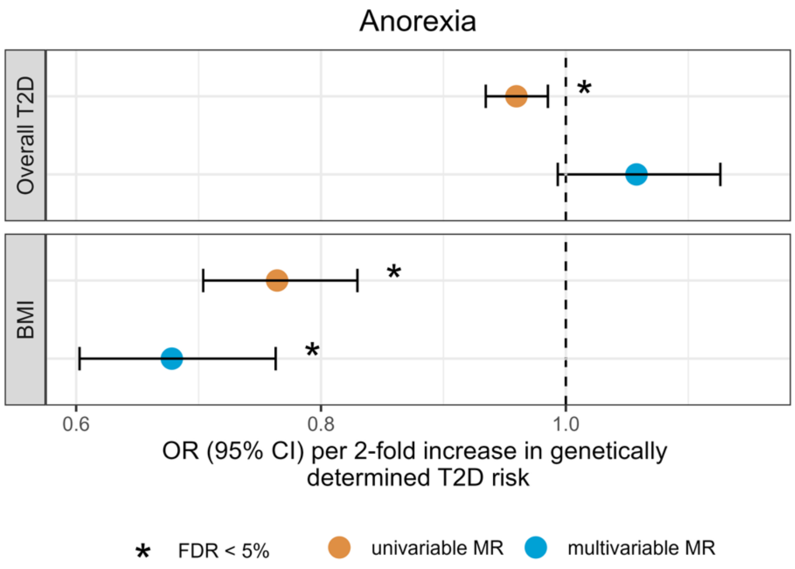
Anorexia
Overall T2D
BMI
*
0.6
0.8
1.0
OR (95% CI) per 2-fold increase in genetically determined T2D risk
FDR < 5%
univariable MR
multivariable MR

14

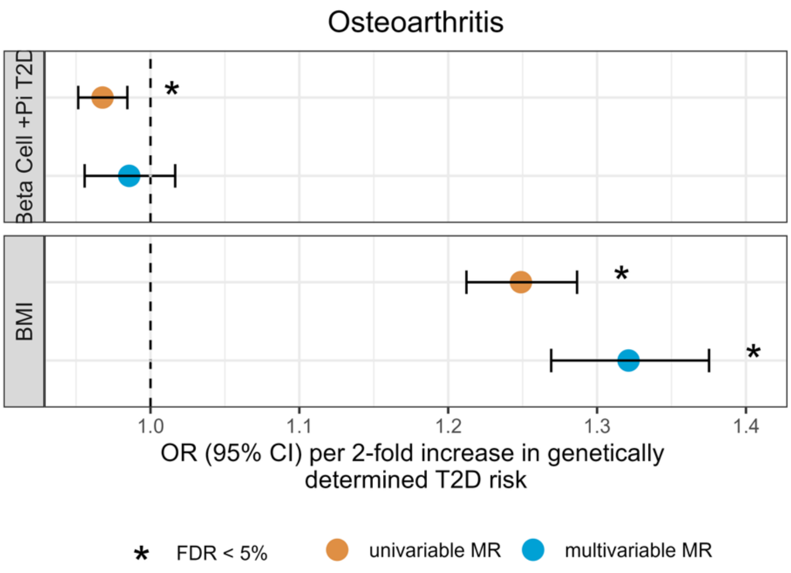
Osteoarthritis
Beta Cell +Pi T2D
BMI
1.0
1.1
1.2
1.3
1.4
OR (95% CI) per 2-fold increase in genetically determined T2D risk
* FDR < 5%
univariable MR
multivariable MR

15

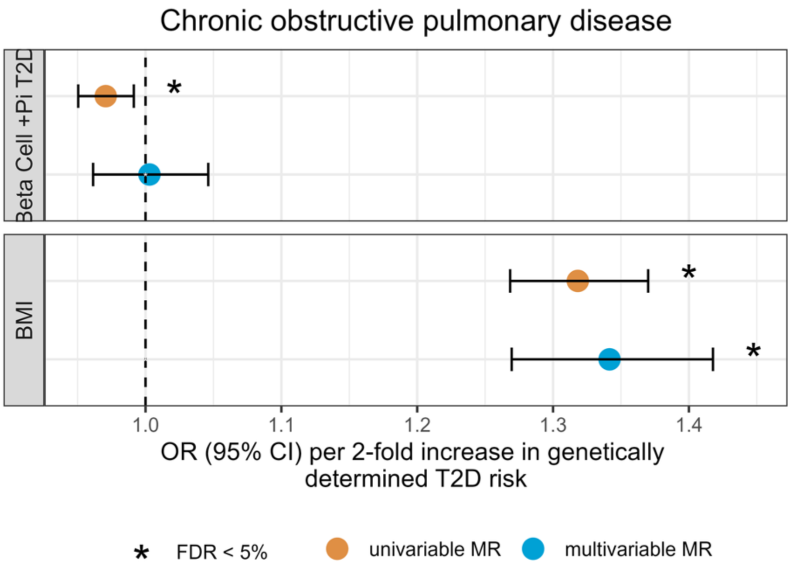
Chronic obstructive pulmonary disease
Beta Cell +Pi T2D
BMI
1.0
1.1
1.2
1.3
1.4
OR (95% CI) per 2-fold increase in genetically determined T2D risk
* FDR < 5%
univariable MR
multivariable MR

16

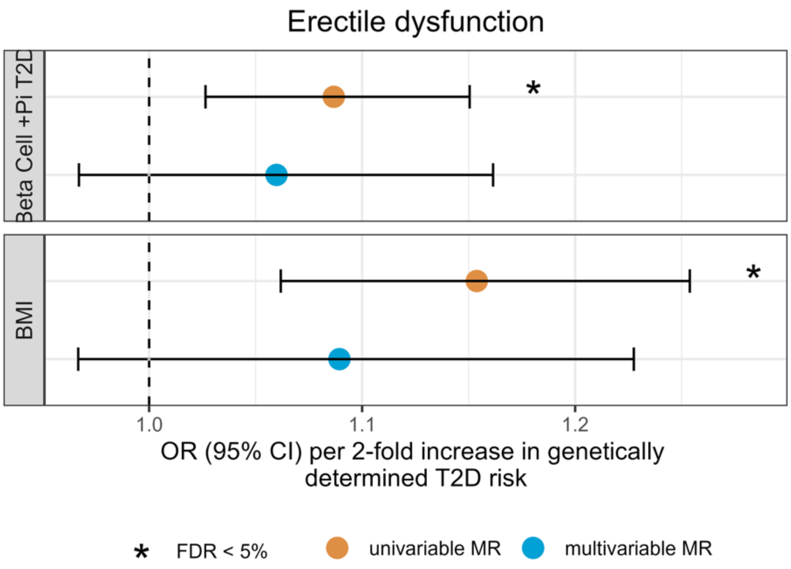
Erectile dysfunction
Beta Cell +Pi T2D
BMI
1.0
1.1
1.2
OR (95% CI) per 2-fold increase in genetically determined T2D risk
* FDR < 5%
univariable MR
multivariable MR

17

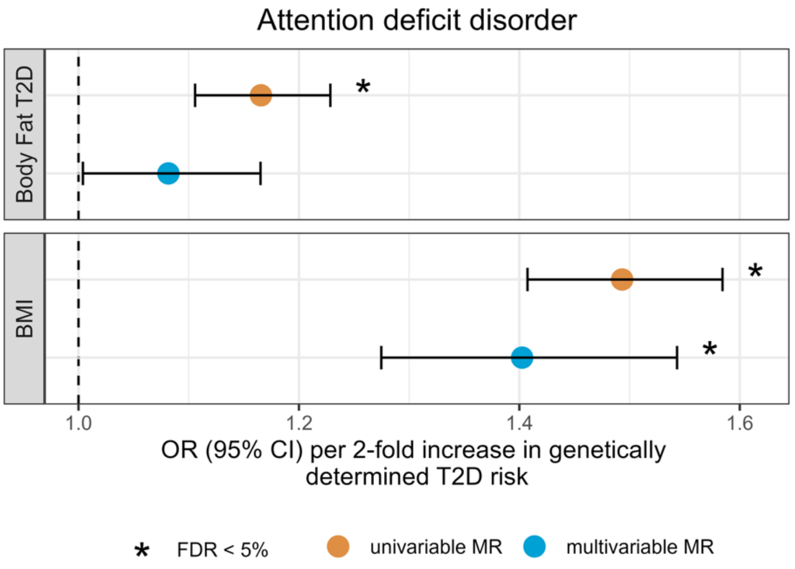

Attention deficit disorder
Body Fat T2D
BMI
*
1.0
1.2
1.4
1.6
OR (95% CI) per 2-fold increase in genetically determined T2D risk
* FDR < 5%
univariable MR
multivariable MR

18

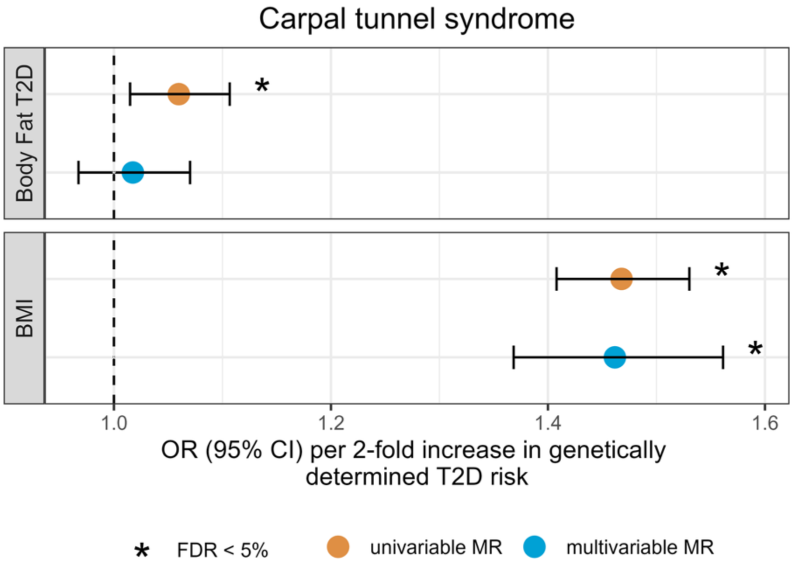

Carpal tunnel syndrome
Body Fat T2D
BMI
*
1.0
1.2
1.4
1.6
OR (95% CI) per 2-fold increase in genetically determined T2D risk
* FDR < 5%
univariable MR
multivariable MR

19

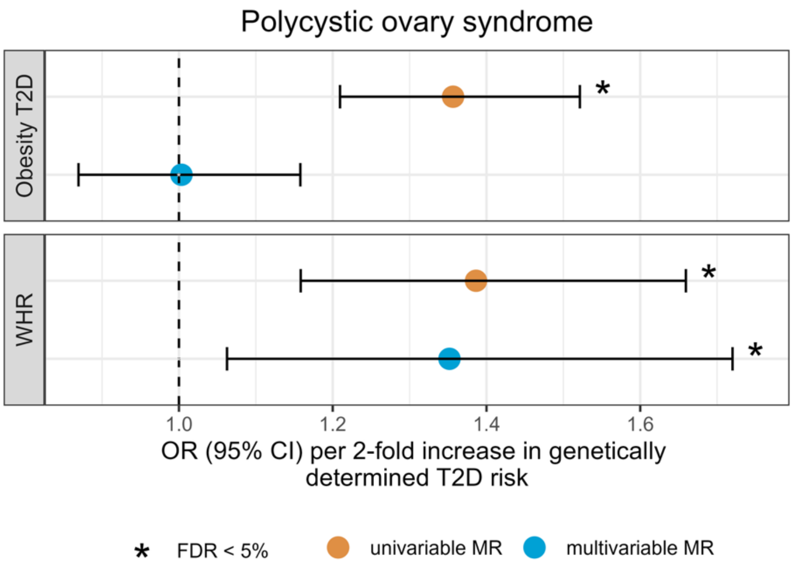

Polycystic ovary syndrome
Obesity T2D
WHR
*
1.0
1.2
1.4
1.6
OR (95% CI) per 2-fold increase in genetically determined T2D risk
* FDR < 5%
univariable MR
multivariable MR

20

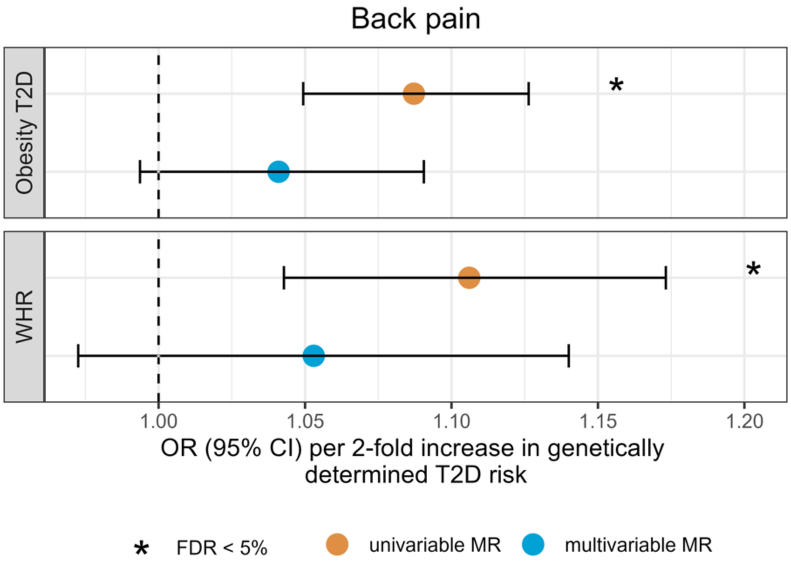

Back pain
Obesity T2D
WHR
*
*
1.00
1.05
1.10
1.15
1.20
OR (95% CI) per 2-fold increase in genetically determined T2D risk
* FDR < 5%
univariable MR
multivariable MR

21

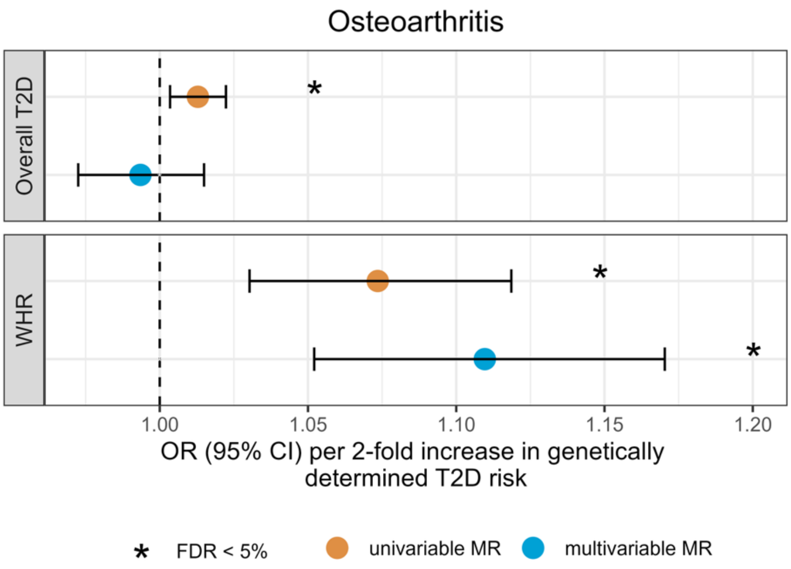

Osteoarthritis
Overall T2D
WHR
*
*
*
1.00
1.05
1.10
1.15
1.20
OR (95% CI) per 2-fold increase in genetically determined T2D risk
* FDR < 5%
univariable MR
multivariable MR

22

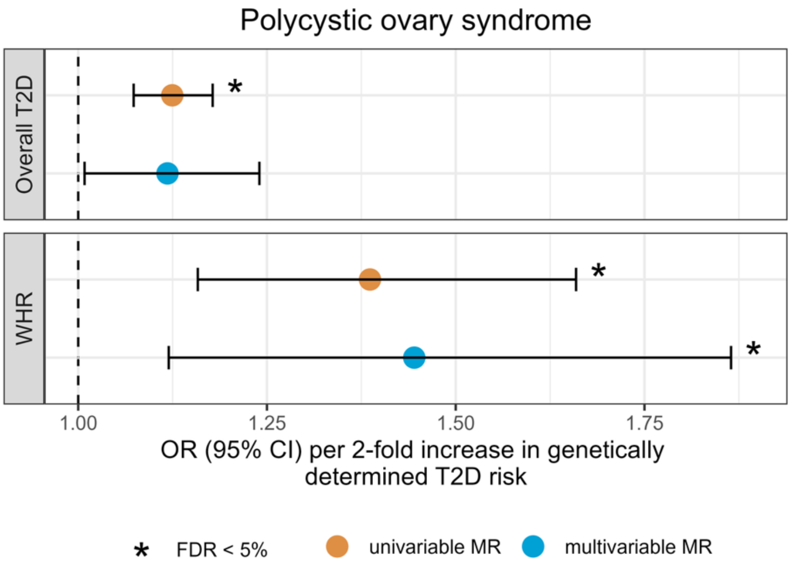

Polycystic ovary syndrome
Overall T2D
WHR
*
*
*
1.00
1.25
1.50
1.75
OR (95% CI) per 2-fold increase in genetically determined T2D risk
* FDR < 5%
univariable MR
multivariable MR

23

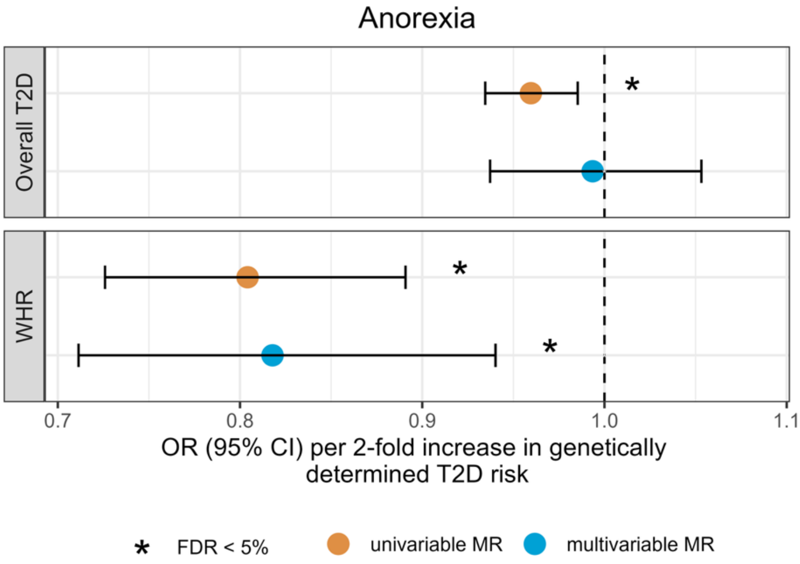

Anorexia
Overall T2D
WHR
0.7
0.8
0.9
1.0
1.1
OR (95% CI) per 2-fold increase in genetically determined T2D risk
* FDR < 5%
univariable MR
multivariable MR

24

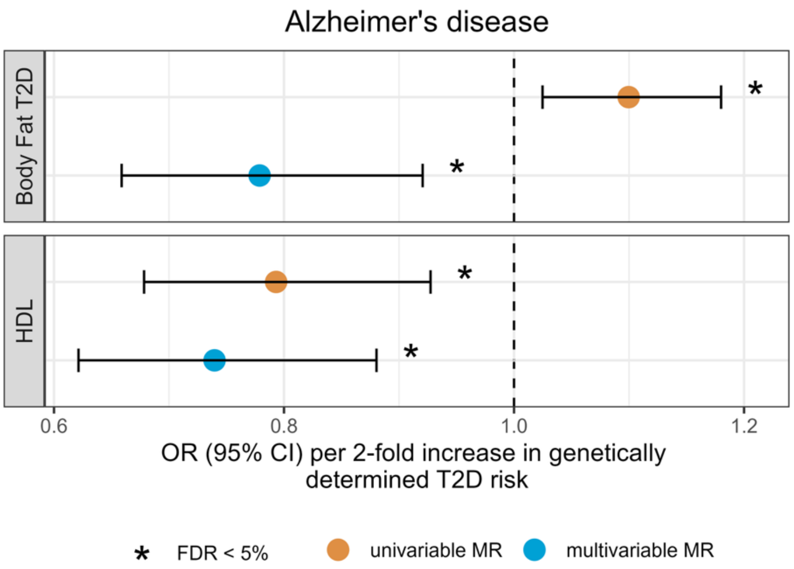

Alzheimer's disease
Body Fat T2D
HDL
0.6
0.8
1.0
1.2
OR (95% CI) per 2-fold increase in genetically determined T2D risk
* FDR < 5%
univariable MR
multivariable MR

25

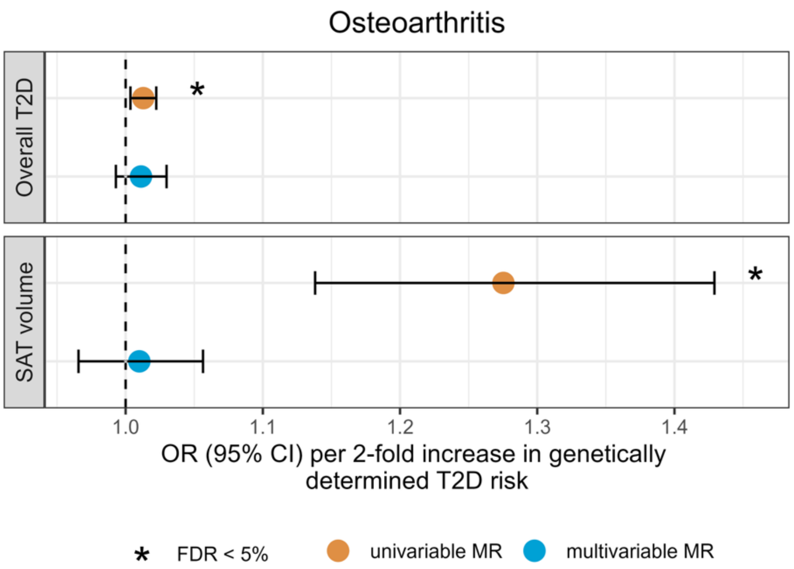

Osteoarthritis
Overall T2D
SAT volume
1.0
1.1
1.2
1.3
1.4
OR (95% CI) per 2-fold increase in genetically determined T2D risk
* FDR < 5%
univariable MR
multivariable MR

26

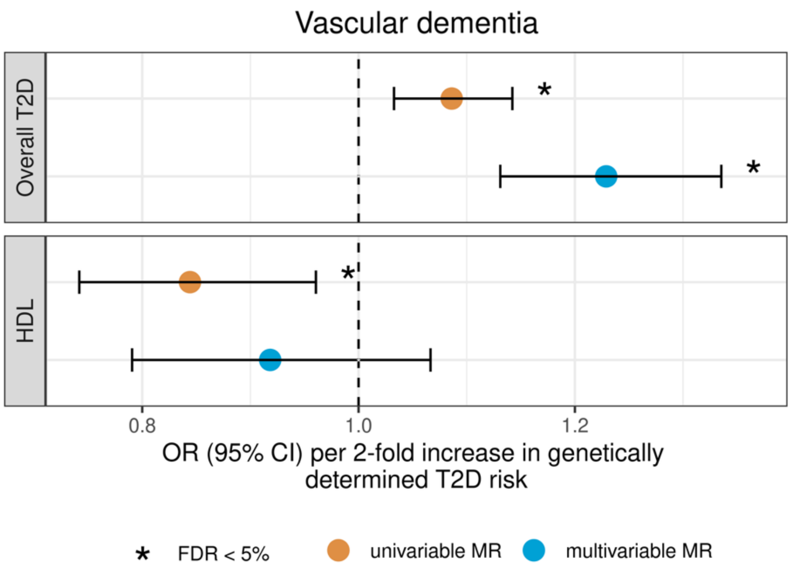

Vascular dementia
Overall T2D
HDL
*
*
*
0.8
1.0
1.2
OR (95% CI) per 2-fold increase in genetically determined T2D risk
* FDR < 5%
univariable MR
multivariable MR

27

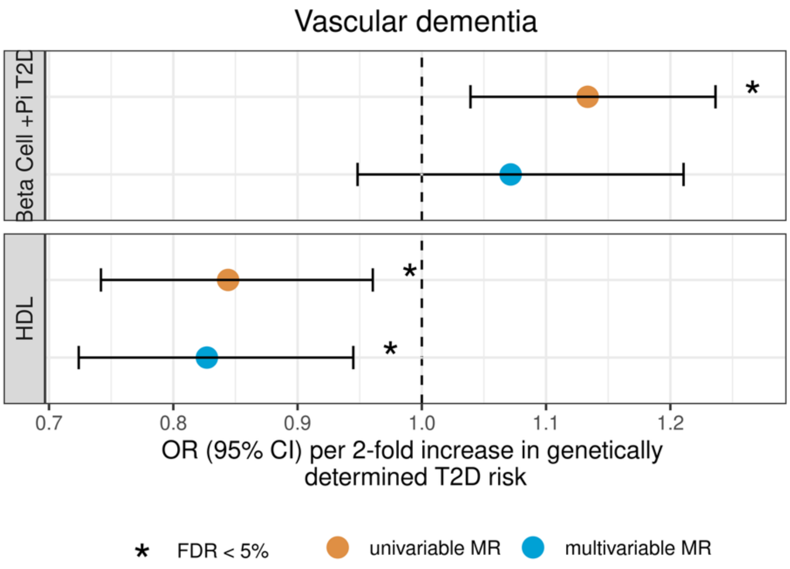

Vascular dementia
Beta Cell +Pi T2D
HDL
*
*
*
0.7
0.8
0.9
1.0
1.1
1.2
OR (95% CI) per 2-fold increase in genetically determined T2D risk
* FDR < 5%
univariable MR
multivariable MR

28

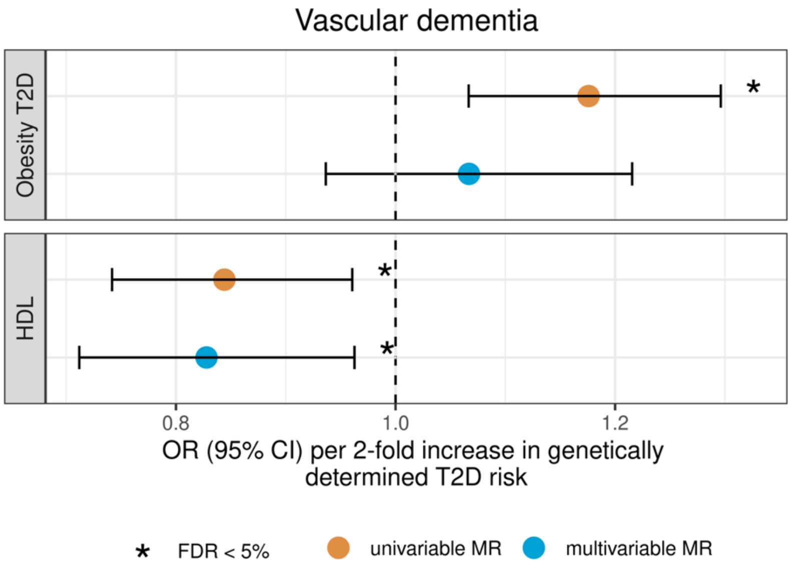

Vascular dementia
Obesity T2D
HDL
*
*
*
0.8
1.0
1.2
OR (95% CI) per 2-fold increase in genetically determined T2D risk
* FDR < 5%
univariable MR
multivariable MR

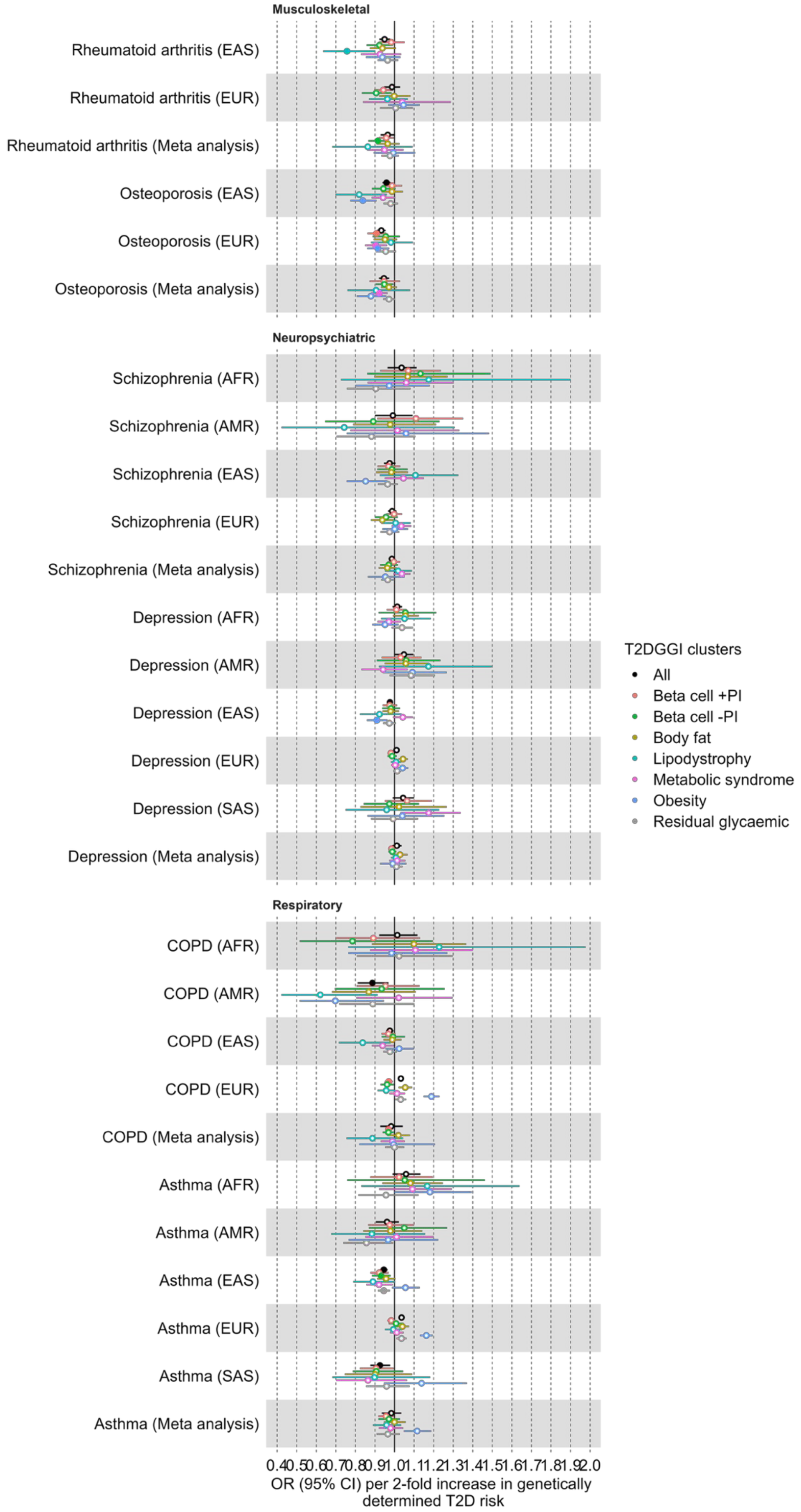

Supplementary Figure 29: Results of the single-ancestry cluster-stratified two-sample Mendelian randomization (MR) analysis of genetic predisposition for type 2 diabetes (T2D) on non-cardiovascular comorbidities risk. Causal estimates are expressed as the odds ratio (OR) of each comorbidity per doubling (2-fold increase) in genetically determined dichotomous T2D risk. Filled circles mark estimates with a q-value < 0.05 that passed all sensitivity analyses to assess the validity of the MR assumptions. (T2DGGI = Type 2 Diabetes Global Genomics Initiative; CI = confidence interval; PI = proinsulin; COPD = chronic obstructive pulmonary disease)

Supplementary Figures 30-55: Comparison between the results of Mendelian randomization (MR) results using the inverse variance weighted method and different approaches to select genetic instrumental variables (IVs). The forest plots depict all cluster-stratified estimates of genetic predisposition for type 2 diabetes (T2D) on comorbidity risk. Causal estimates are expressed as the odds ratio of comorbidity risk per doubling (2-fold increase) in genetically determined dichotomous T2D risk. Filled circles mark estimates with FDR < 5%. The genetic ancestry groups represent individuals genetically similar to Africans (AFR), East Asians (EAS), Europeans (EUR), admixed Americans (AMR) and South Asians (SAS) as defined by the 1000 Genomes Project. (CI = confidence interval).

30

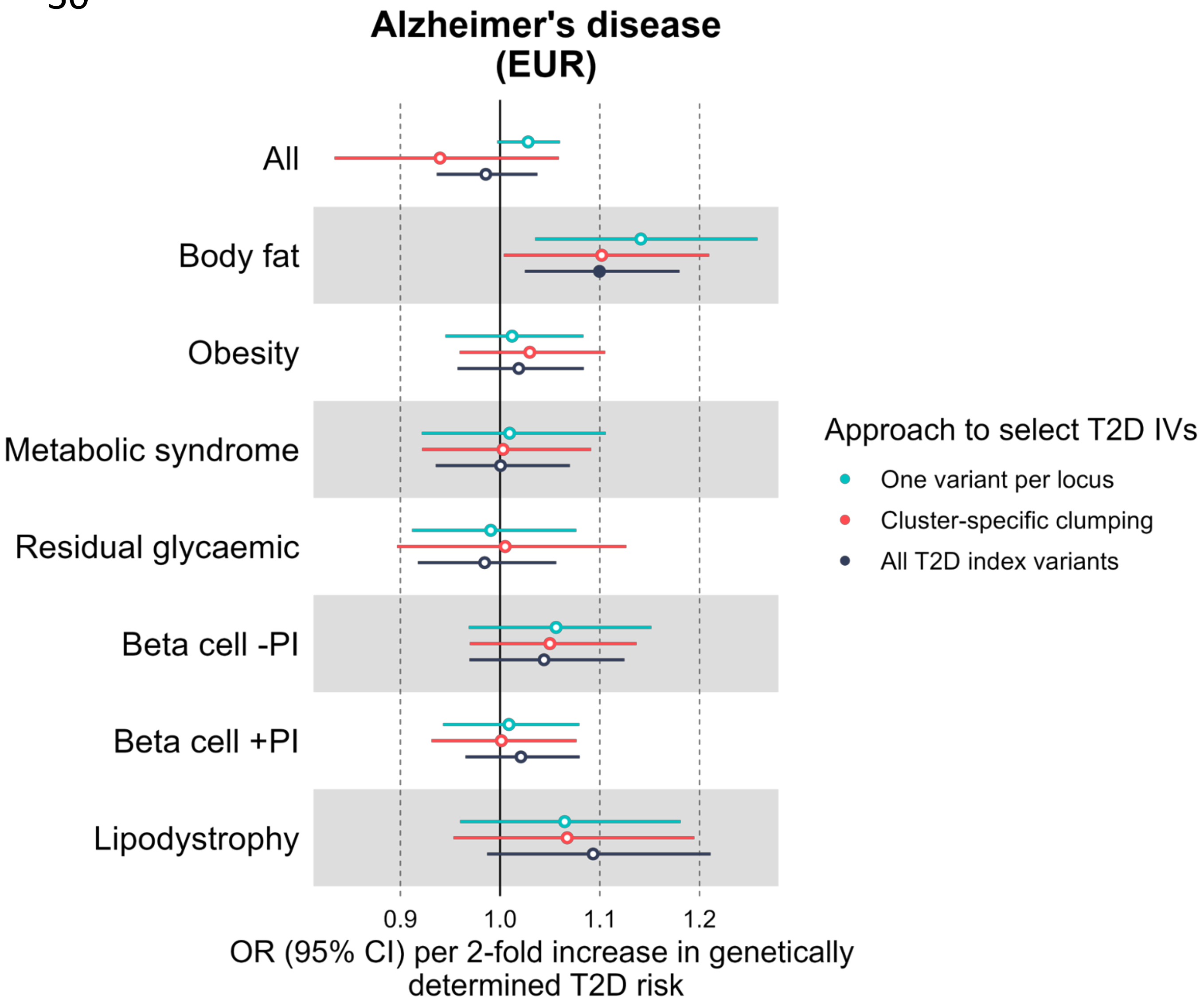

Alzheimer's disease
(EUR)
All
Body fat
Obesity
Metabolic syndrome
Residual glycaemic
Beta cell -PI
Beta cell +PI
Lipodystrophy
0.9
1.0
1.1
1.2
OR (95% CI) per 2-fold increase in genetically determined T2D risk
Approach to select T2D IVs
One variant per locus
Cluster-specific clumping
All T2D index variants

31

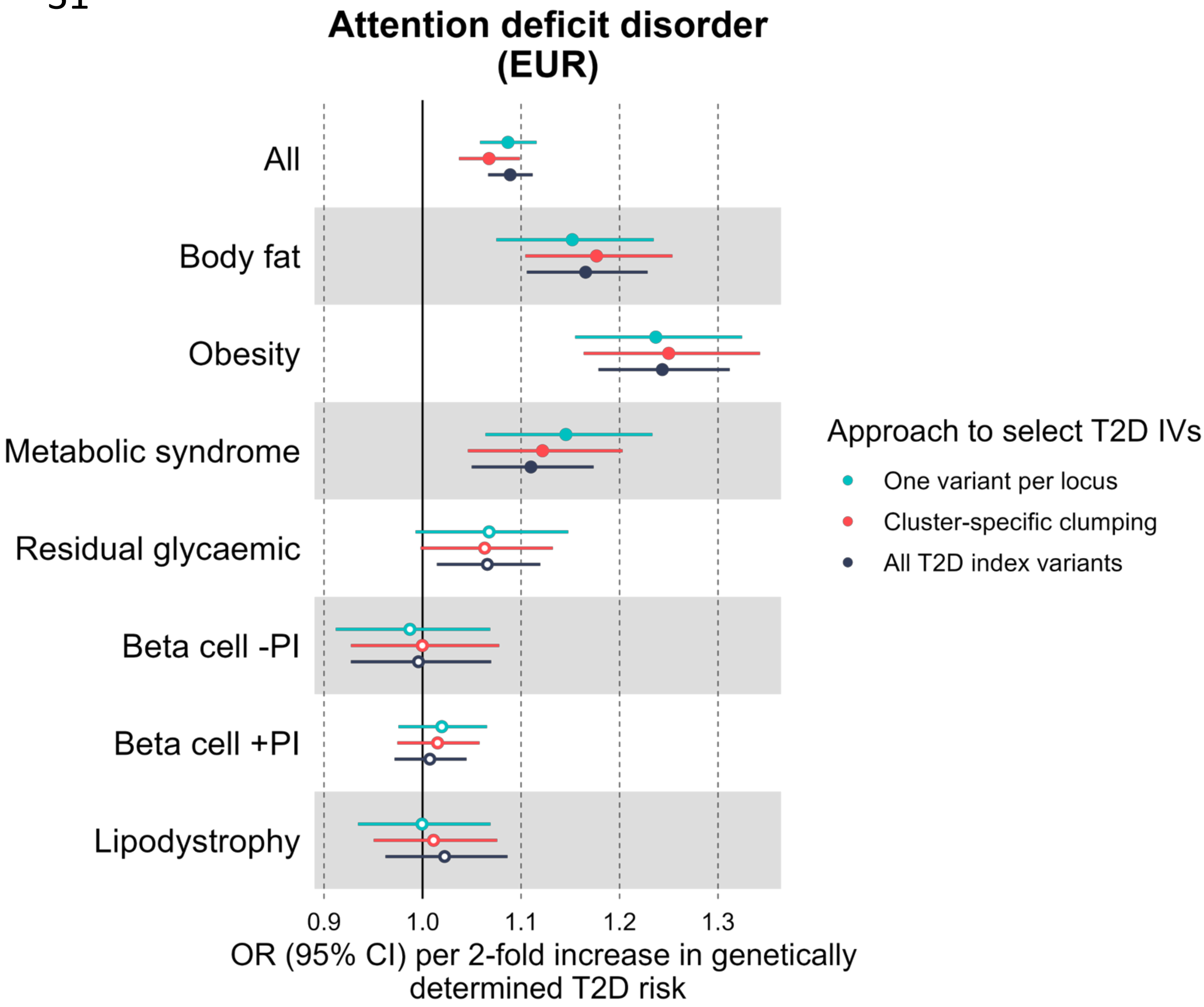

Attention deficit disorder
(EUR)
All
Body fat
Obesity
Metabolic syndrome
Residual glycaemic
Beta cell -PI
Beta cell +PI
Lipodystrophy
0.9
1.0
1.1
1.2
1.3
OR (95% CI) per 2-fold increase in genetically determined T2D risk
Approach to select T2D IVs
One variant per locus
Cluster-specific clumping
All T2D index variants

32

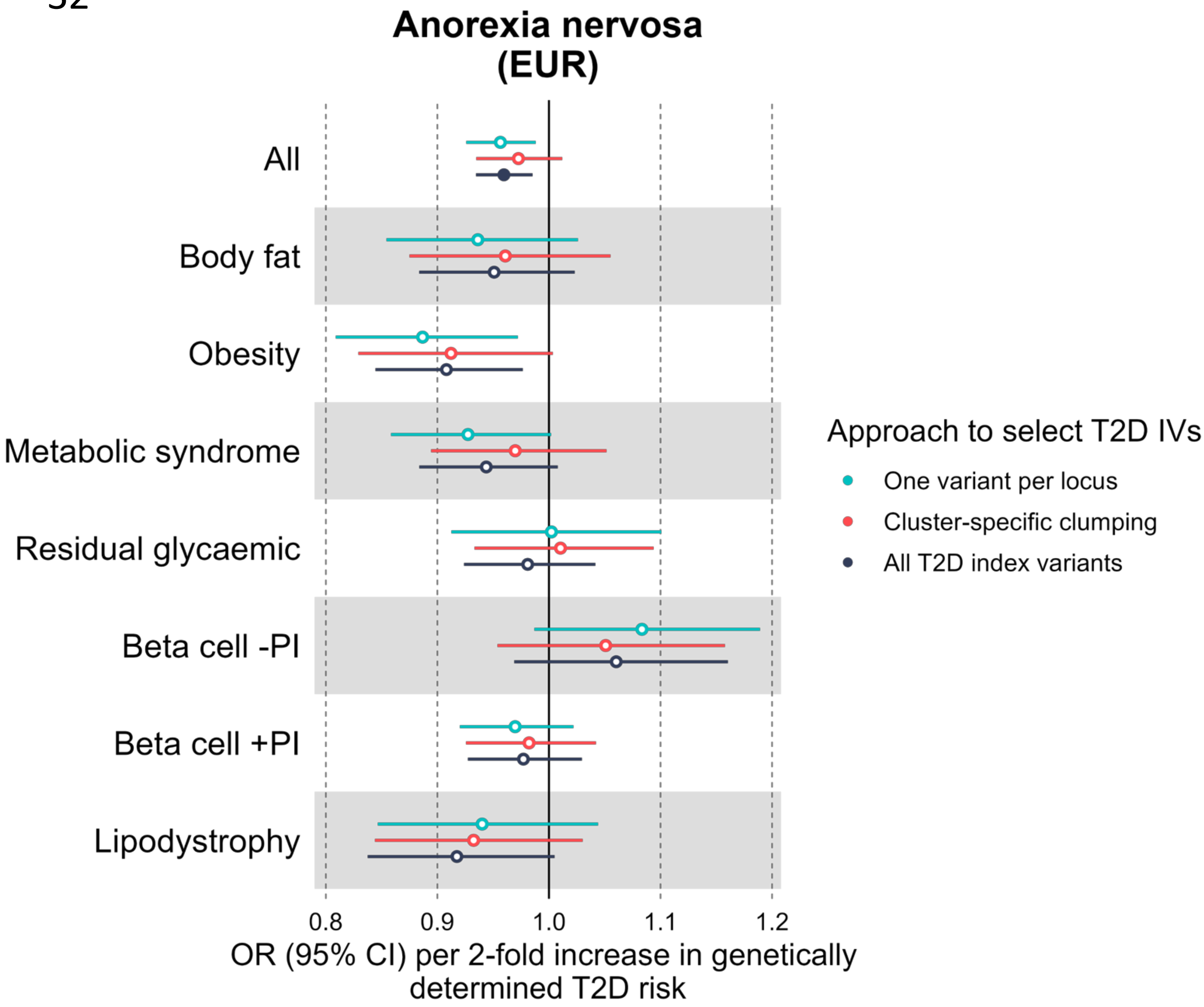

Anorexia nervosa
(EUR)
All
Body fat
Obesity
Metabolic syndrome
Residual glycaemic
Beta cell -PI
Beta cell +PI
Lipodystrophy
0.8
0.9
1.0
1.1
1.2
OR (95% CI) per 2-fold increase in genetically determined T2D risk
Approach to select T2D IVs
One variant per locus
Cluster-specific clumping
All T2D index variants

33

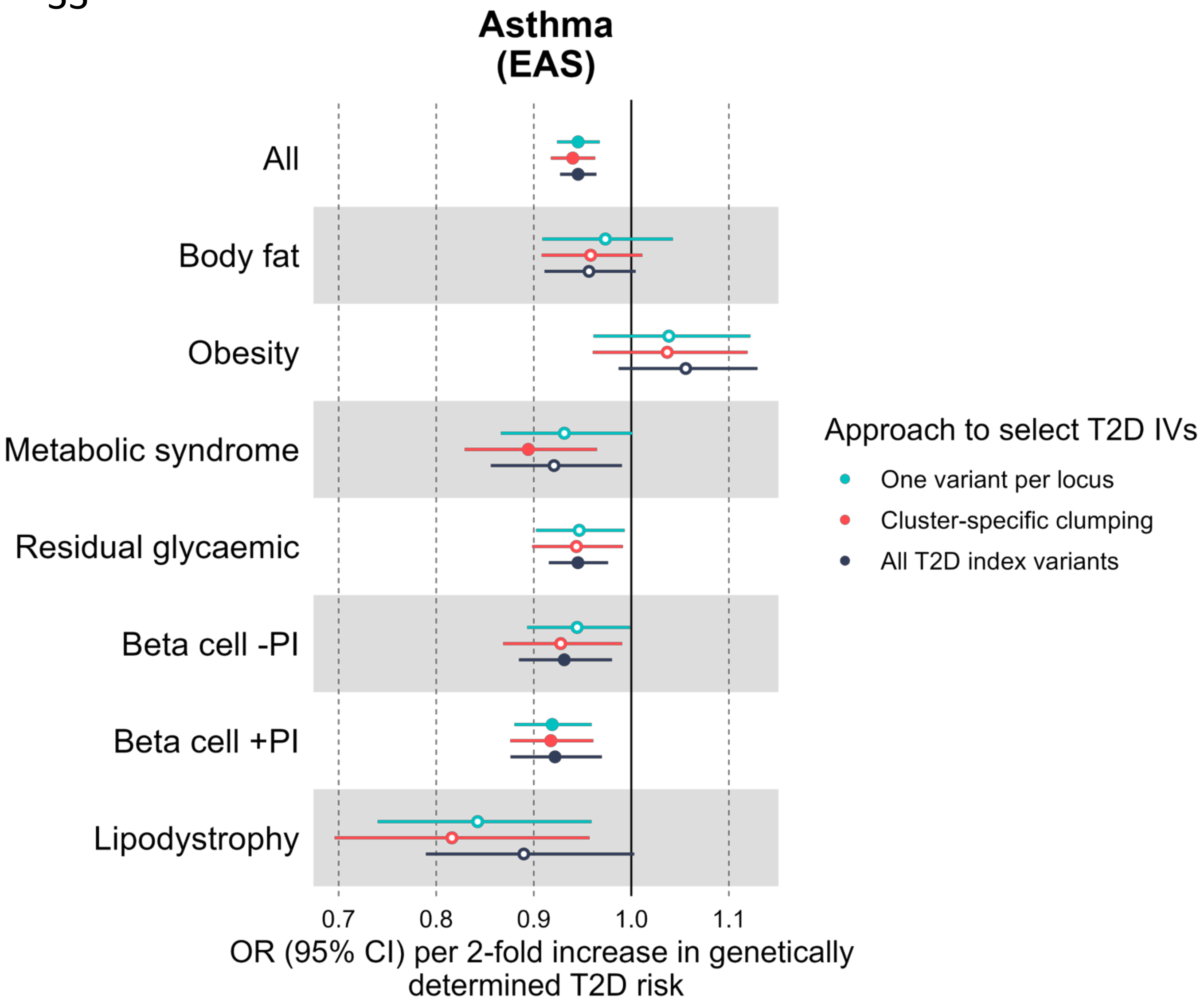

Asthma
(EAS)
All
Body fat
Obesity
Metabolic syndrome
Residual glycaemic
Beta cell -PI
Beta cell +PI
Lipodystrophy
0.7
0.8
0.9
1.0
1.1
OR (95% CI) per 2-fold increase in genetically determined T2D risk
Approach to select T2D IVs
One variant per locus
Cluster-specific clumping
All T2D index variants

34

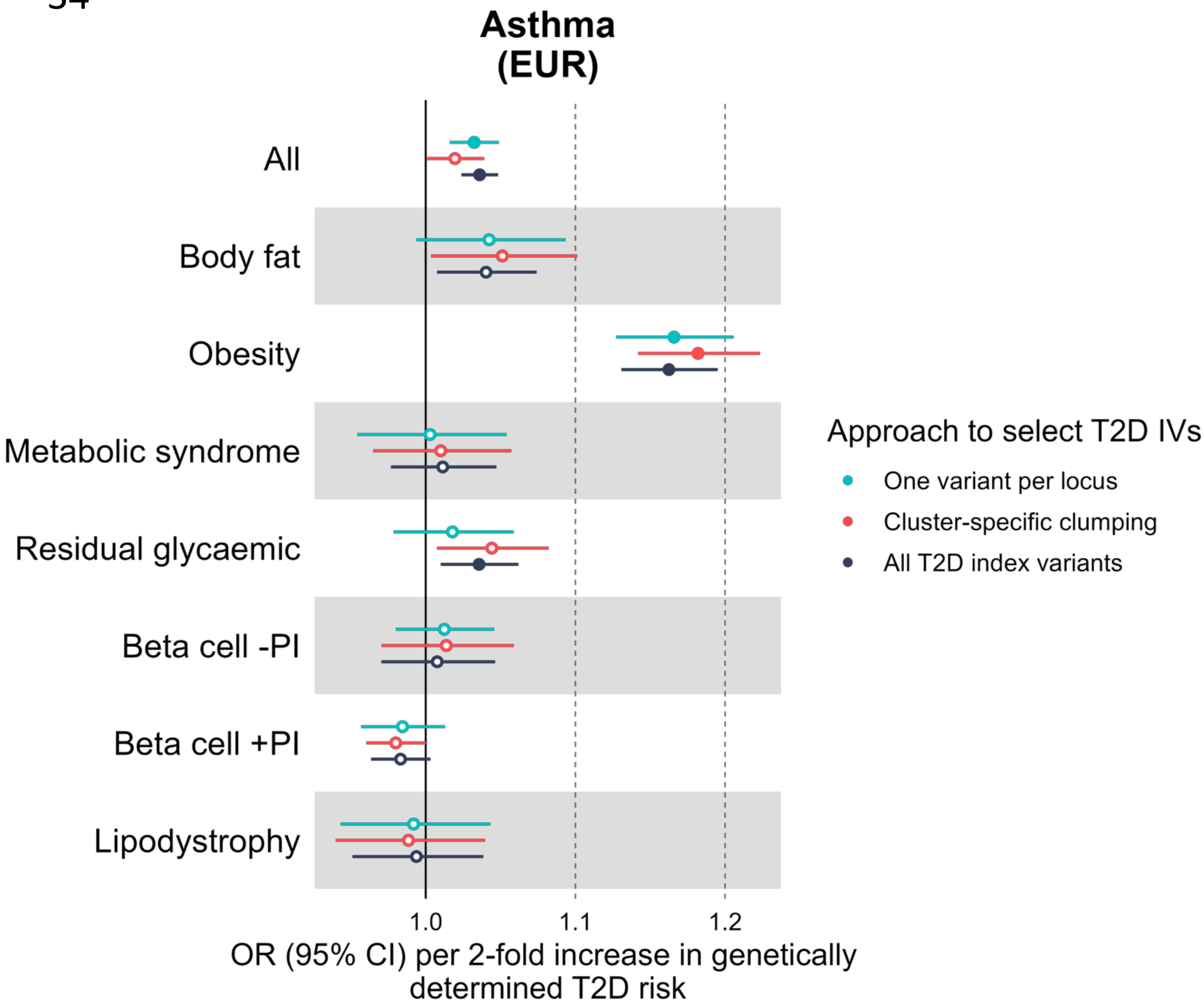

Asthma
(EUR)
All
Body fat
Obesity
Metabolic syndrome
Residual glycaemic
Beta cell -PI
Beta cell +PI
Lipodystrophy
1.0
1.1
1.2
OR (95% CI) per 2-fold increase in genetically determined T2D risk
Approach to select T2D IVs
One variant per locus
Cluster-specific clumping
All T2D index variants

35

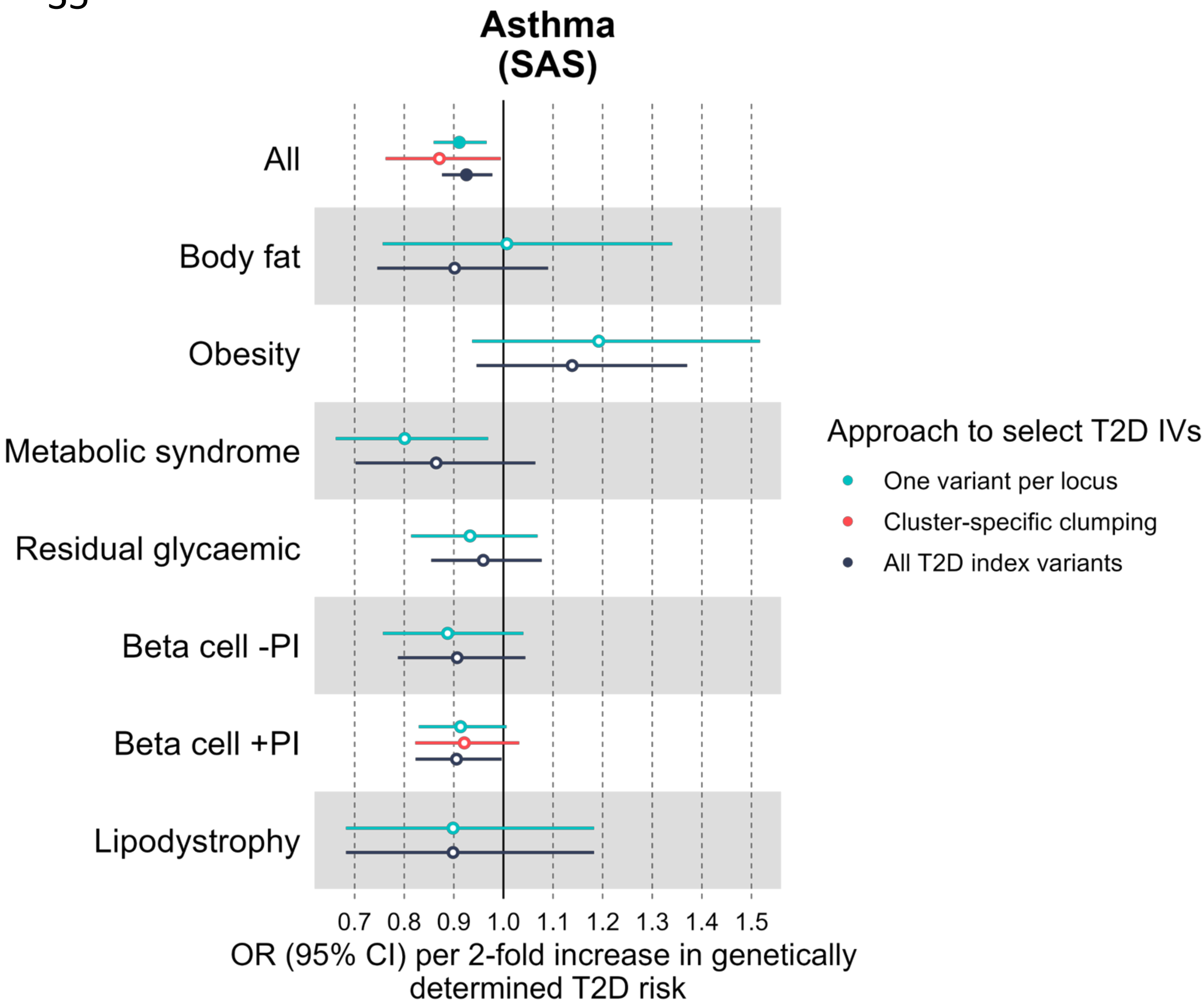

Asthma
(SAS)
All
Body fat
Obesity
Metabolic syndrome
Residual glycaemic
Beta cell -PI
Beta cell +PI
Lipodystrophy
0.7 0.8 0.9 1.0 1.1 1.2 1.3 1.4 1.5
OR (95% CI) per 2-fold increase in genetically determined T2D risk
Approach to select T2D IVs
One variant per locus
Cluster-specific clumping
All T2D index variants

36

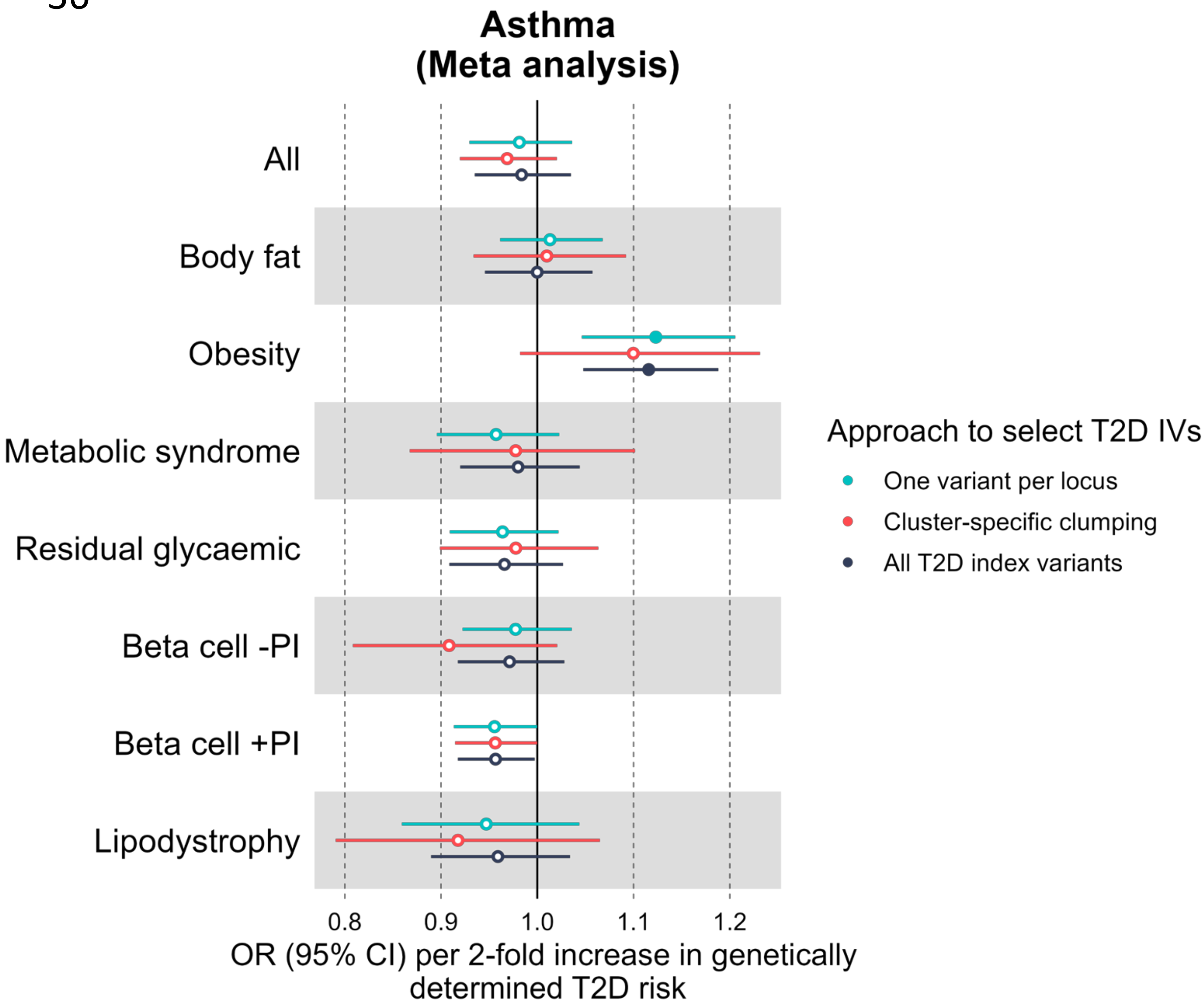

Asthma
(Meta analysis)
All
Body fat
Obesity
Metabolic syndrome
Residual glycaemic
Beta cell -PI
Beta cell +PI
Lipodystrophy
0.8
0.9
1.0
1.1
1.2
OR (95% CI) per 2-fold increase in genetically determined T2D risk
Approach to select T2D IVs
One variant per locus
Cluster-specific clumping
All T2D index variants

37

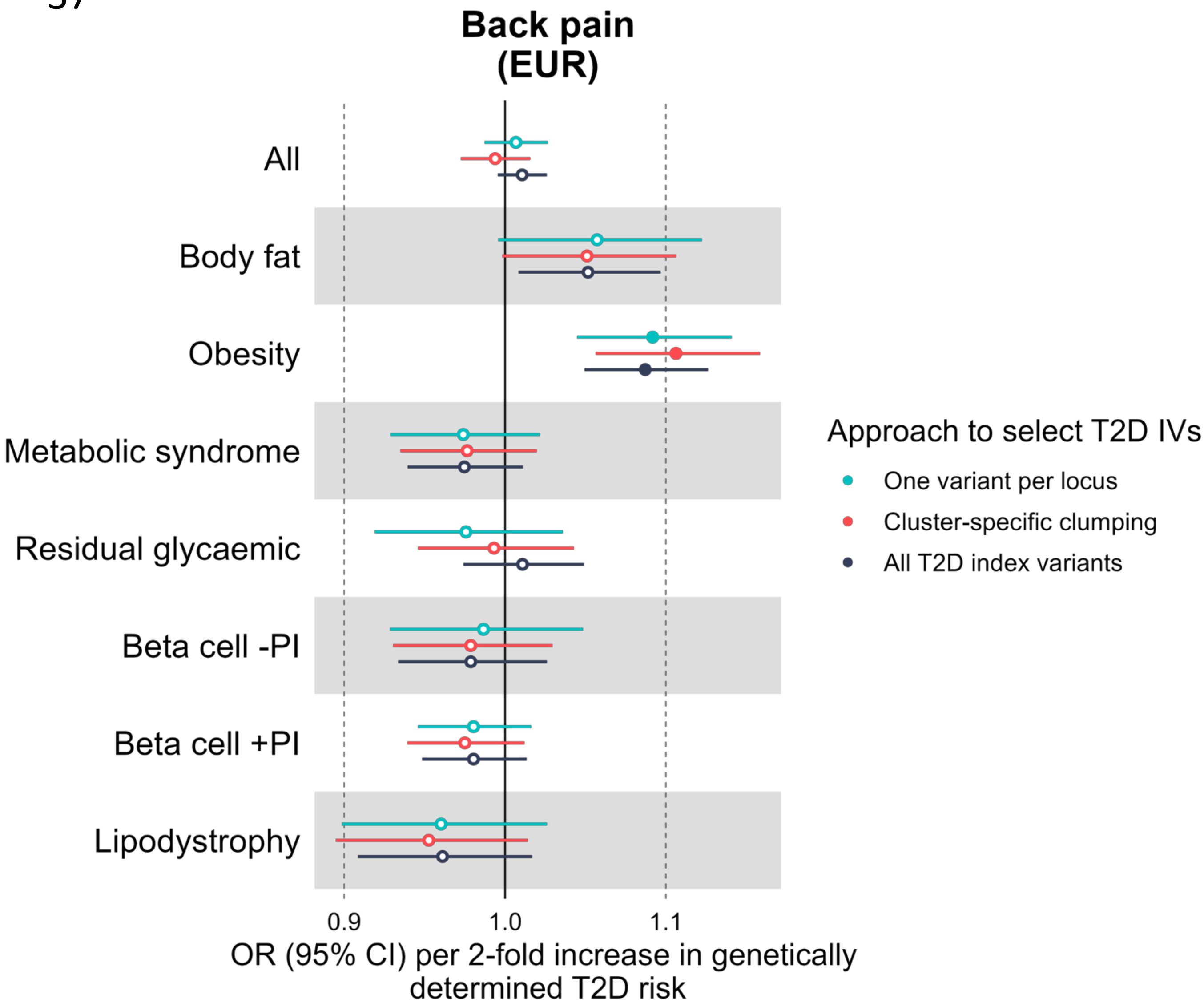

Back pain
(EUR)
All
Body fat
Obesity
Metabolic syndrome
Residual glycaemic
Beta cell -PI
Beta cell +PI
Lipodystrophy
0.9
1.0
1.1
OR (95% CI) per 2-fold increase in genetically determined T2D risk
Approach to select T2D IVs
One variant per locus
Cluster-specific clumping
All T2D index variants

38

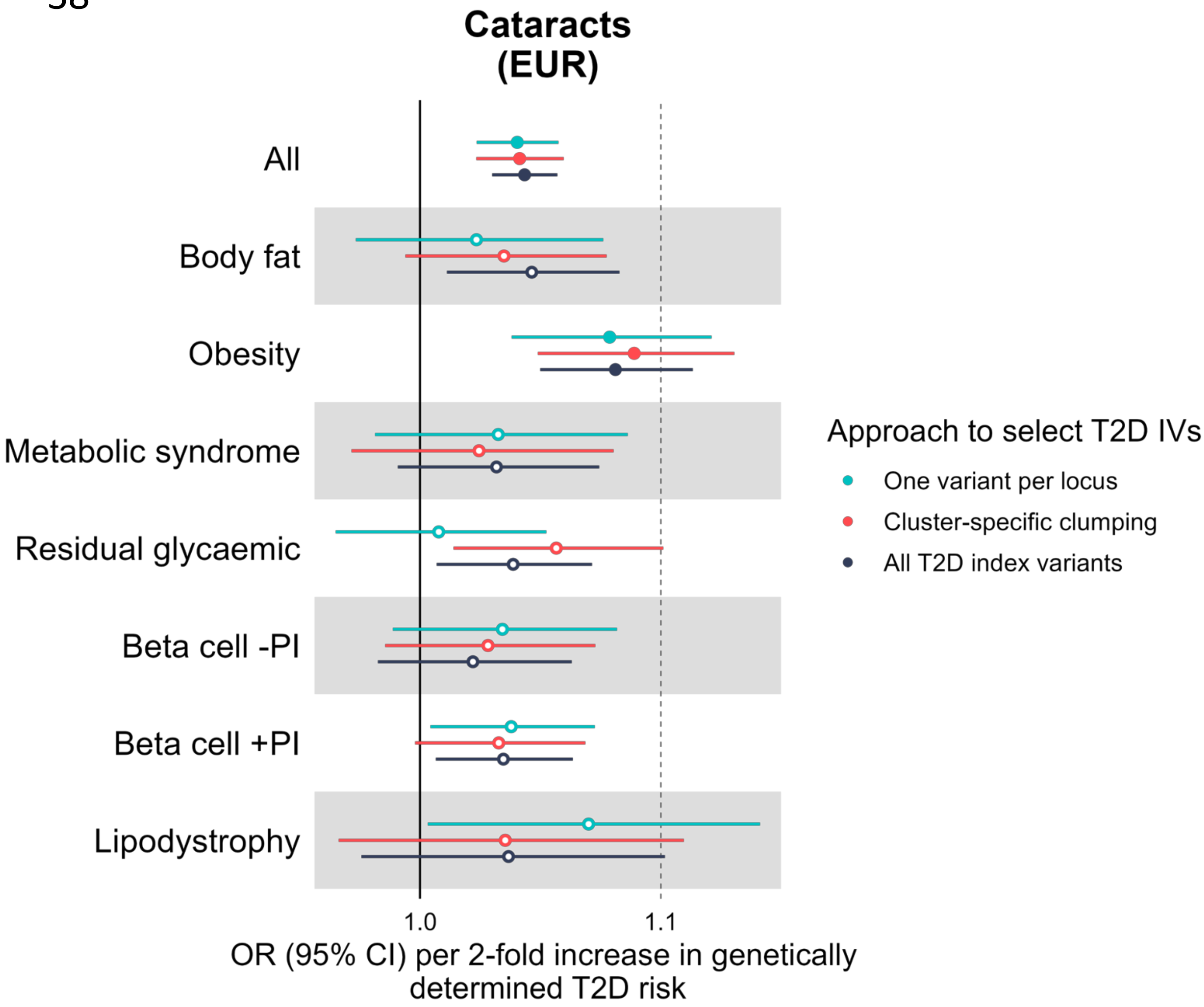

Cataracts
(EUR)
All
Body fat
Obesity
Metabolic syndrome
Residual glycaemic
Beta cell -PI
Beta cell +PI
Lipodystrophy
1.0
1.1
OR (95% CI) per 2-fold increase in genetically determined T2D risk
Approach to select T2D IVs
One variant per locus
Cluster-specific clumping
All T2D index variants

39

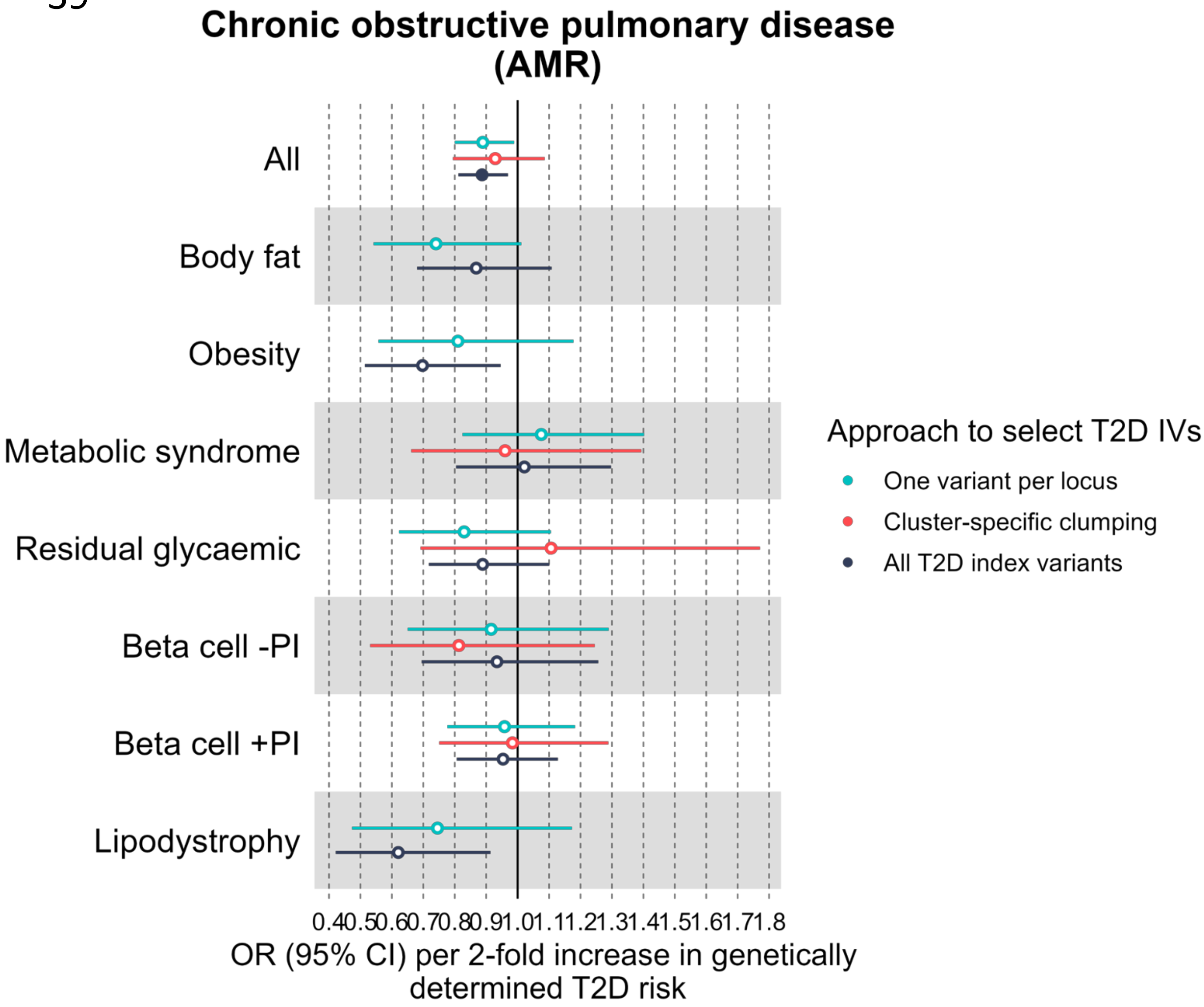
Chronic obstructive pulmonary disease
(AMR)
All
Body fat
Obesity
Metabolic syndrome
Residual glycaemic
Beta cell -PI
Beta cell +PI
Lipodystrophy
0.4 0.5 0.6 0.7 0.8 0.9 1.0 1.1 1.2 1.3 1.4 1.5 1.6 1.7 1.8
OR (95% CI) per 2-fold increase in genetically determined T2D risk
Approach to select T2D IVs
One variant per locus
Cluster-specific clumping
All T2D index variants

40

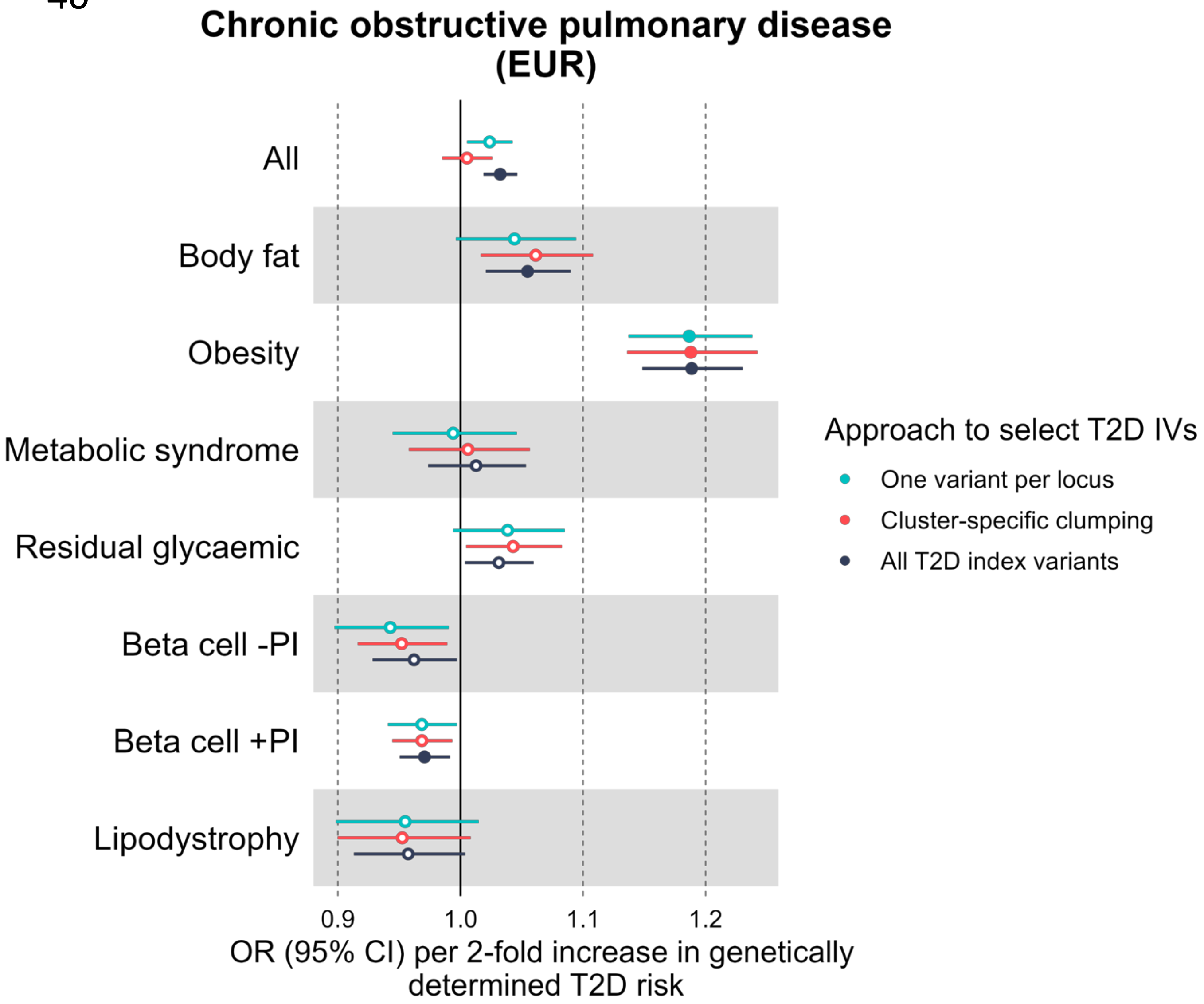
Chronic obstructive pulmonary disease
(EUR)
All
Body fat
Obesity
Metabolic syndrome
Residual glycaemic
Beta cell -PI
Beta cell +PI
Lipodystrophy
0.9
1.0
1.1
1.2
OR (95% CI) per 2-fold increase in genetically
determined T2D risk
Approach to select T2D IVs
One variant per locus
Cluster-specific clumping
All T2D index variants

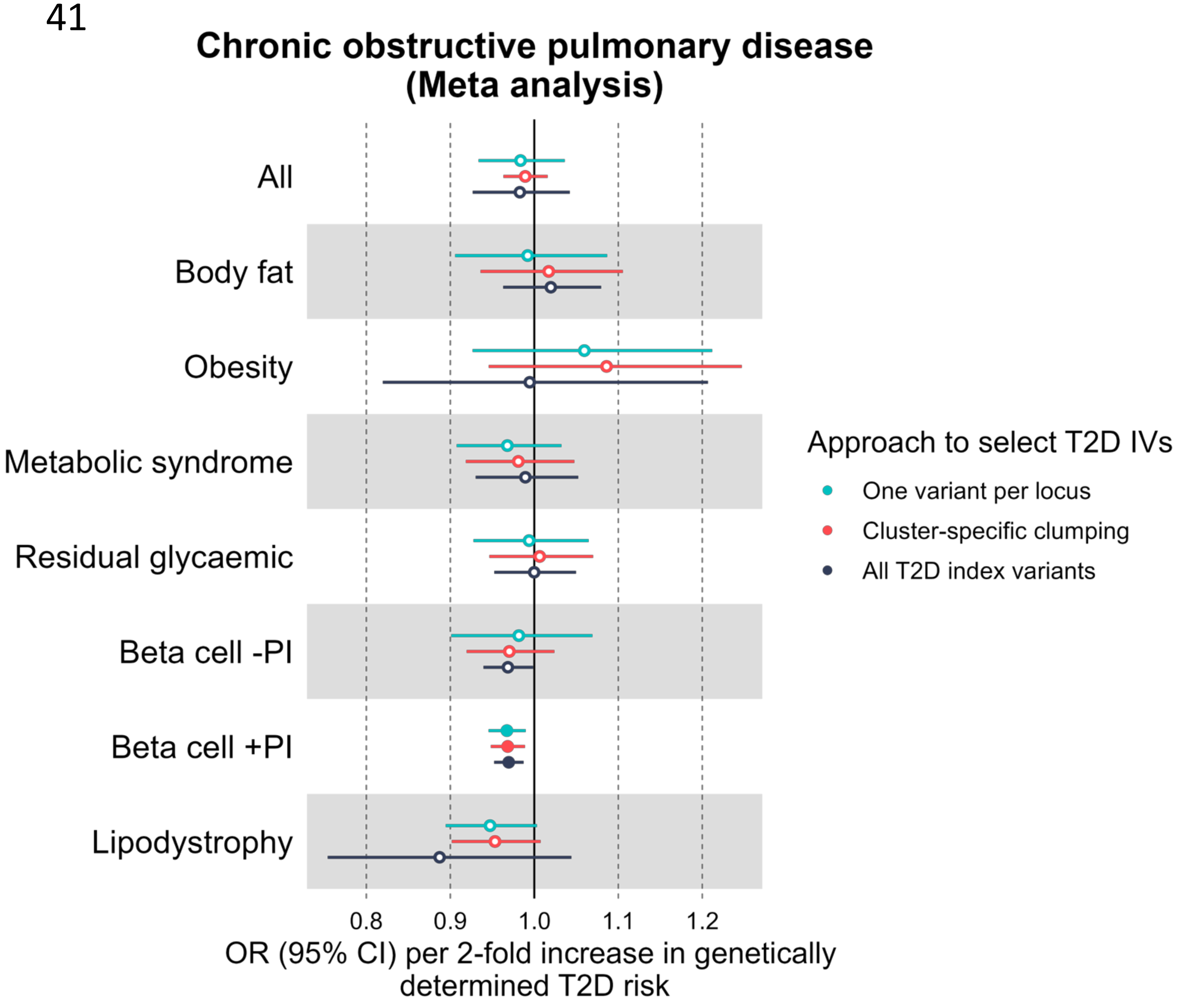
Chronic obstructive pulmonary disease
(Meta analysis)
All
Body fat
Obesity
Metabolic syndrome
Residual glycaemic
Beta cell -PI
Beta cell +PI
Lipodystrophy
0.8
0.9
1.0
1.1
1.2
OR (95% CI) per 2-fold increase in genetically determined T2D risk
Approach to select T2D IVs
One variant per locus
Cluster-specific clumping
All T2D index variants

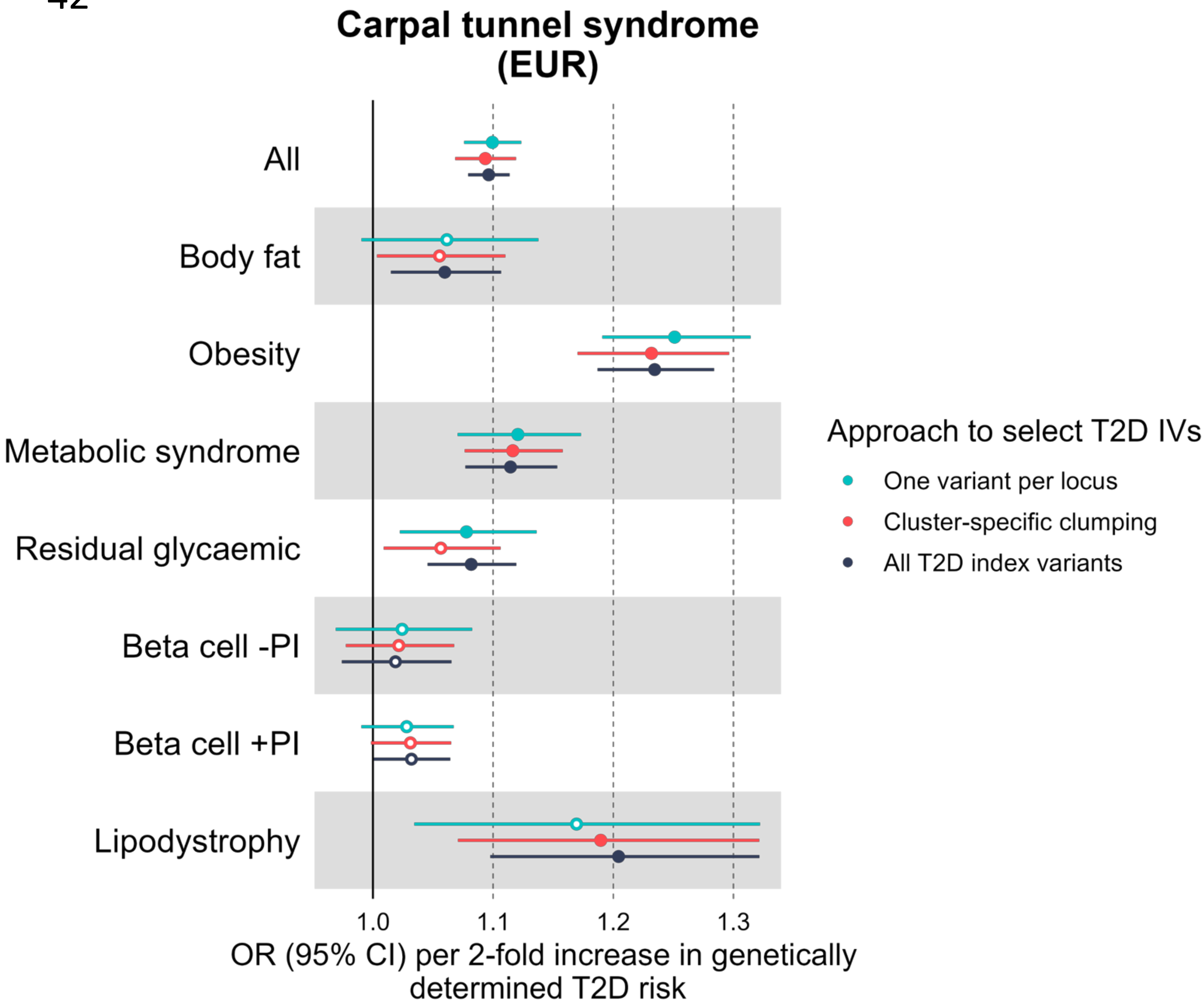
Carpal tunnel syndrome (EUR)
All
Body fat
Obesity
Metabolic syndrome
Residual glycaemic
Beta cell -PI
Beta cell +PI
Lipodystrophy
1.0
1.1
1.2
1.3
OR (95% CI) per 2-fold increase in genetically determined T2D risk
Approach to select T2D IVs
One variant per locus
Cluster-specific clumping
All T2D index variants

43

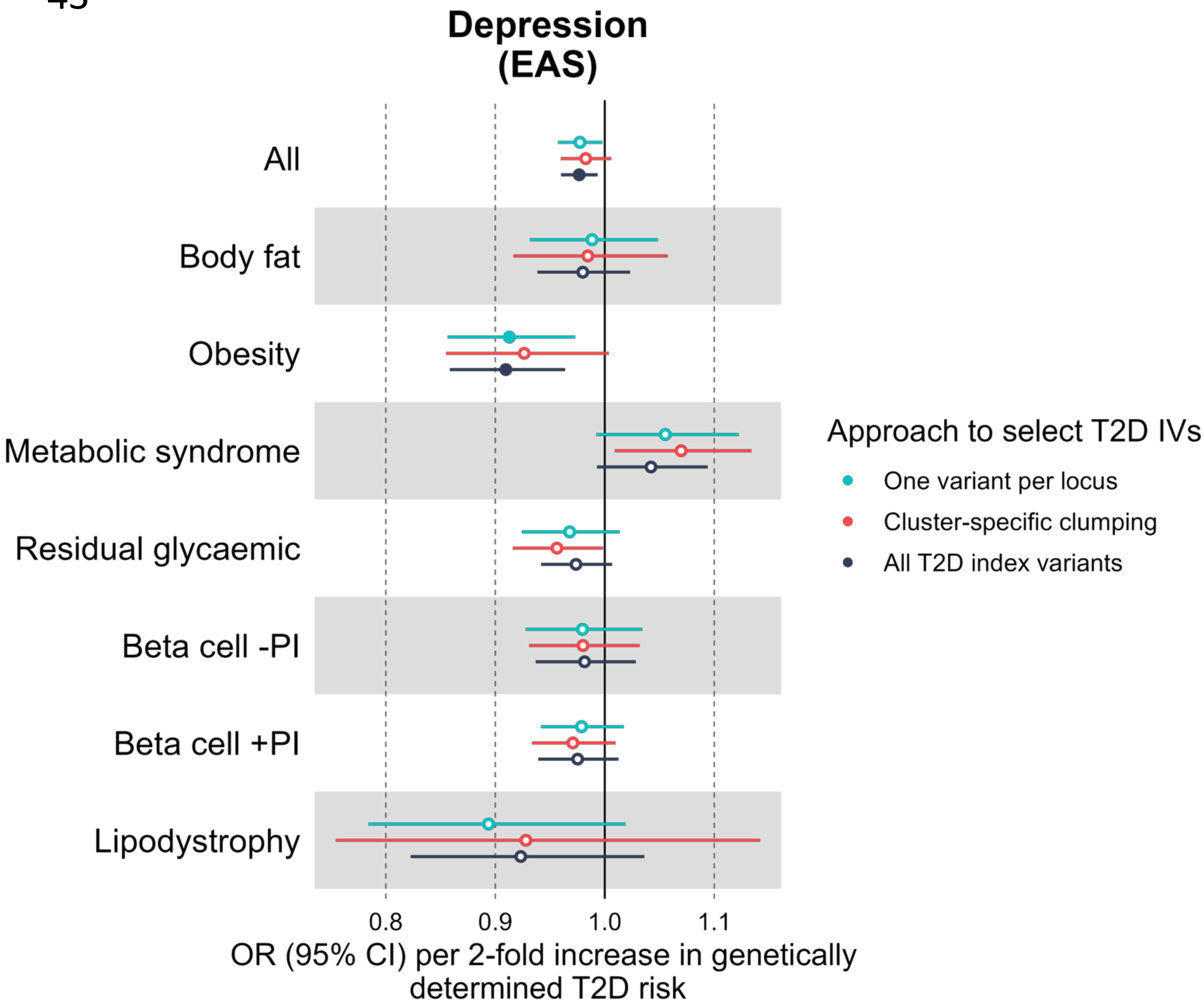

Depression
(EAS)
All
Body fat
Obesity
Metabolic syndrome
Residual glycaemic
Beta cell -PI
Beta cell +PI
Lipodystrophy
0.8
0.9
1.0
1.1
OR (95% CI) per 2-fold increase in genetically determined T2D risk
Approach to select T2D IVs
One variant per locus
Cluster-specific clumping
All T2D index variants

44

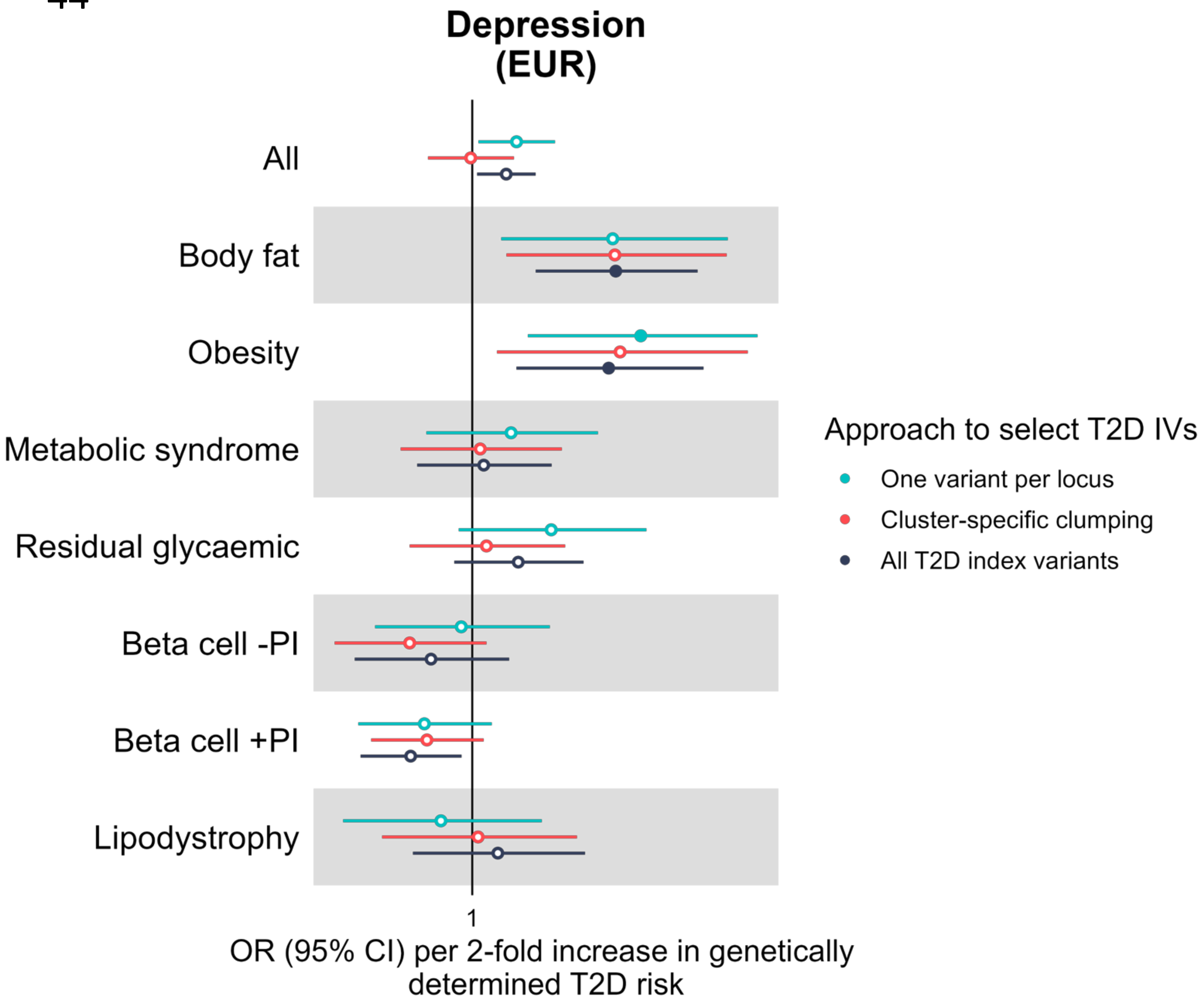

Depression
(EUR)
All
Body fat
Obesity
Metabolic syndrome
Residual glycaemic
Beta cell -PI
Beta cell +PI
Lipodystrophy
1
OR (95% CI) per 2-fold increase in genetically determined T2D risk
Approach to select T2D IVs
One variant per locus
Cluster-specific clumping
All T2D index variants

45

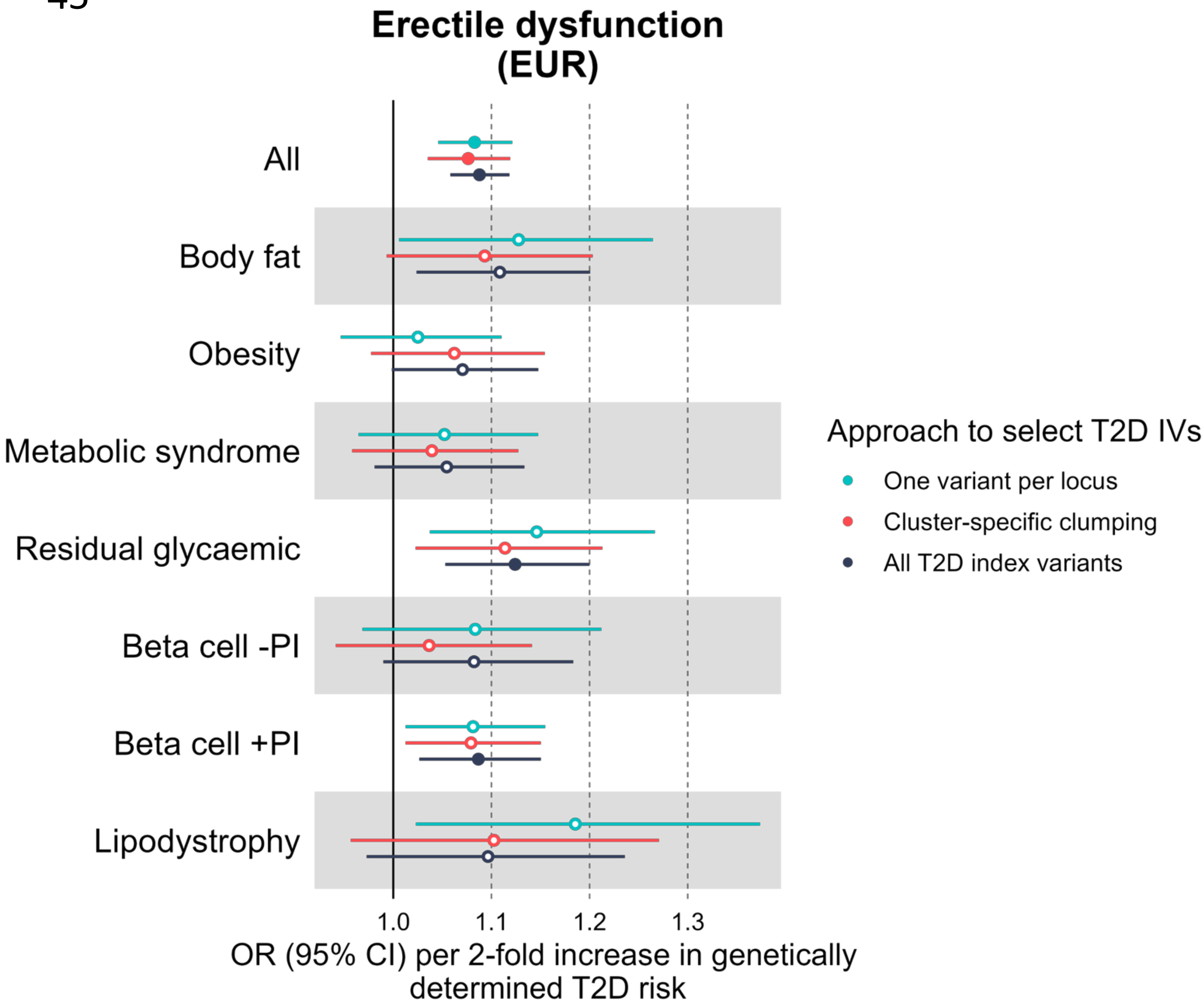

Erectile dysfunction
(EUR)
All
Body fat
Obesity
Metabolic syndrome
Residual glycaemic
Beta cell -PI
Beta cell +PI
Lipodystrophy
1.0
1.1
1.2
1.3
OR (95% CI) per 2-fold increase in genetically determined T2D risk
Approach to select T2D IVs
One variant per locus
Cluster-specific clumping
All T2D index variants

46

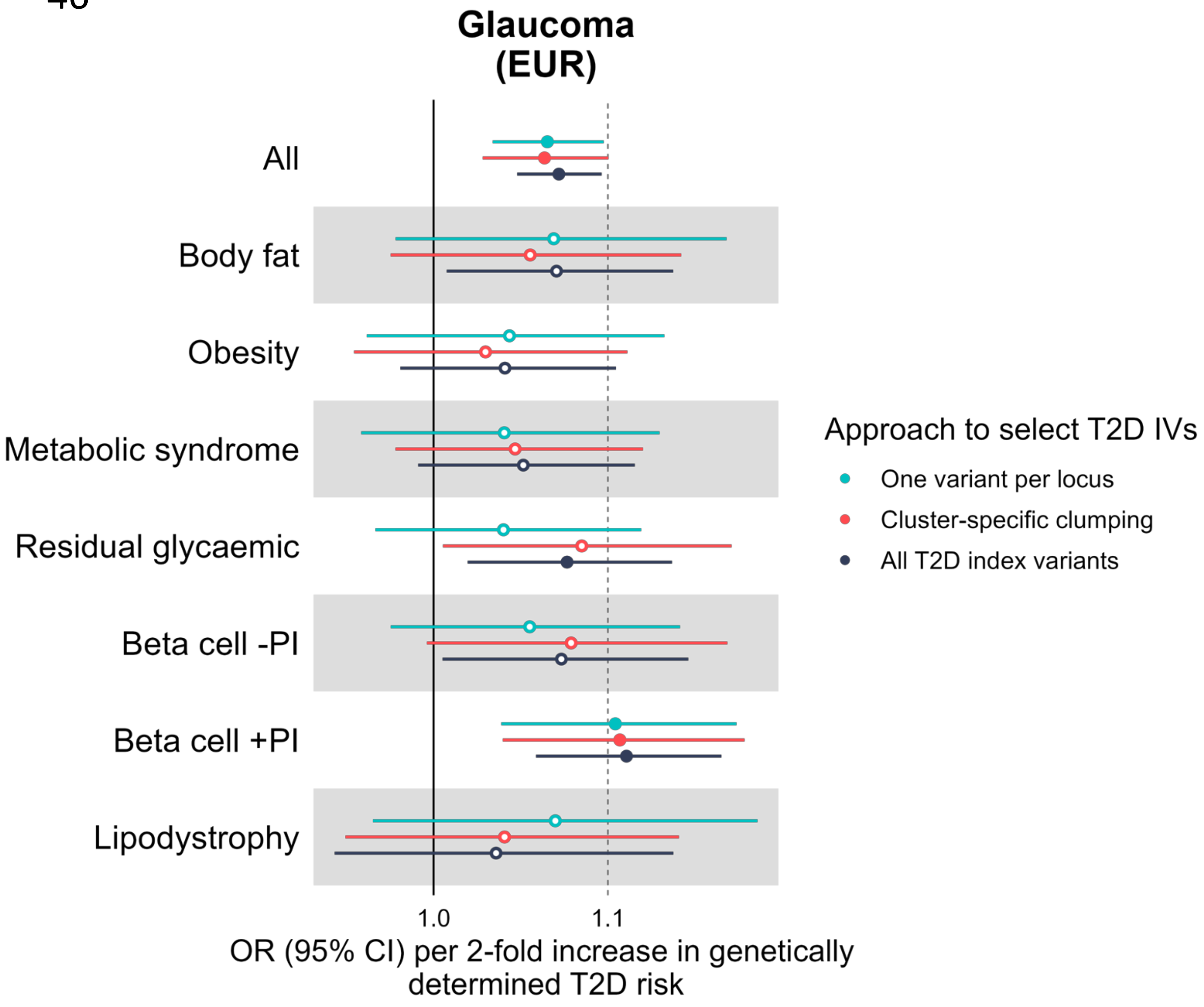

Glaucoma
(EUR)
All
Body fat
Obesity
Metabolic syndrome
Residual glycaemic
Beta cell -PI
Beta cell +PI
Lipodystrophy
1.0
1.1
OR (95% CI) per 2-fold increase in genetically determined T2D risk
Approach to select T2D IVs
One variant per locus
Cluster-specific clumping
All T2D index variants

47

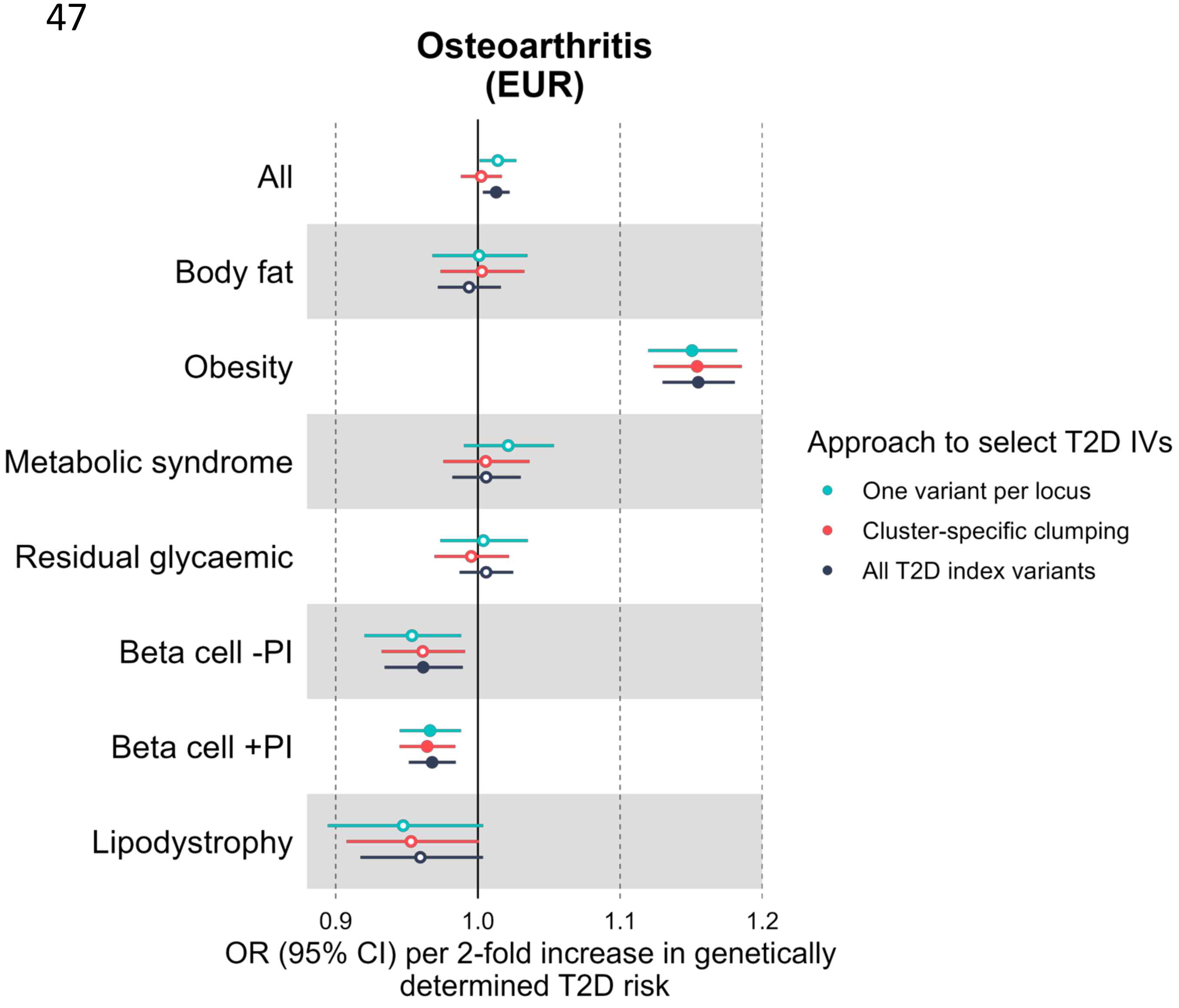

Osteoarthritis
(EUR)
All
Body fat
Obesity
Metabolic syndrome
Residual glycaemic
Beta cell -PI
Beta cell +PI
Lipodystrophy
0.9
1.0
1.1
1.2
OR (95% CI) per 2-fold increase in genetically
determined T2D risk
Approach to select T2D IVs
One variant per locus
Cluster-specific clumping
All T2D index variants

48

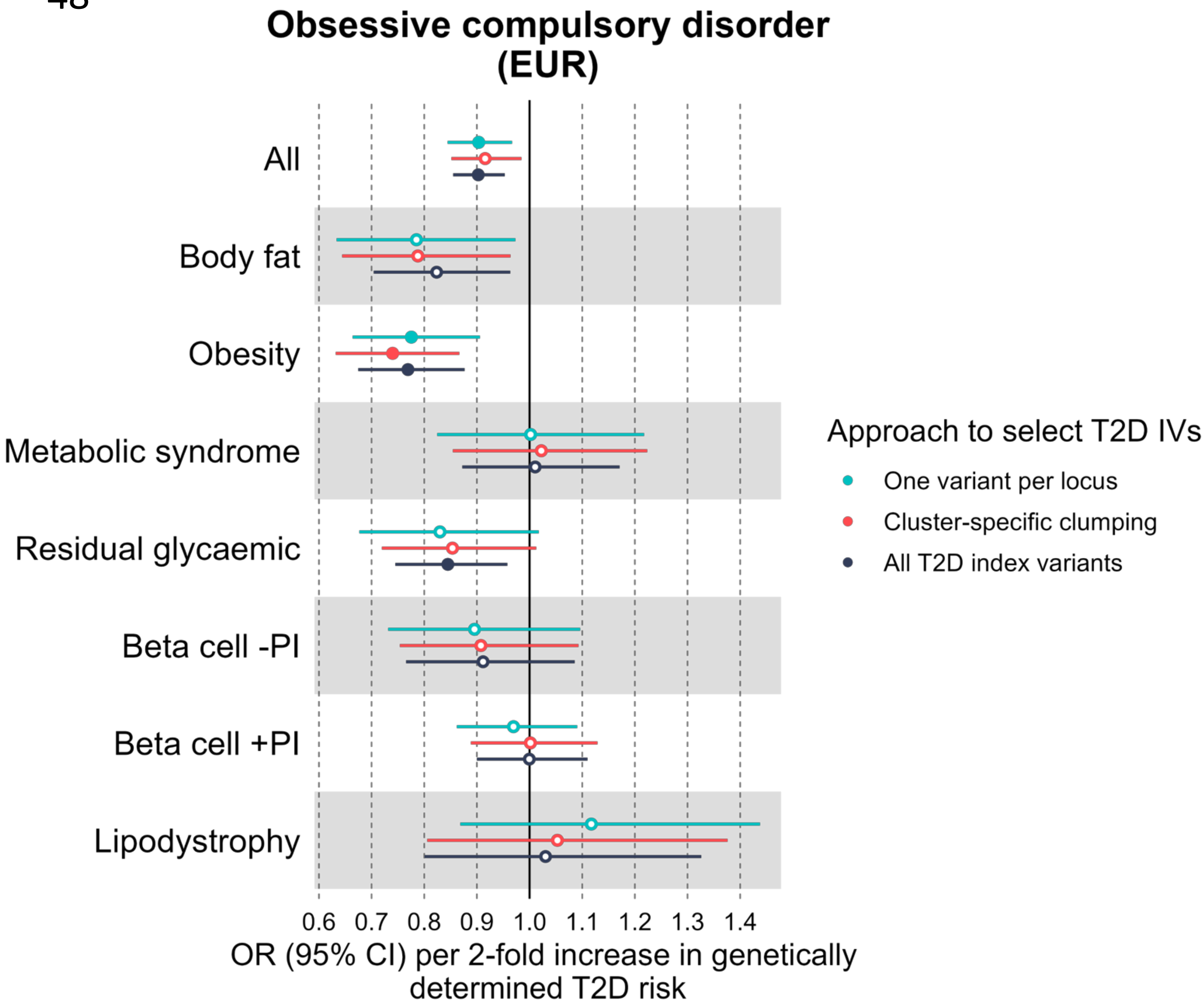

Obsessive compulsory disorder
(EUR)
All
Body fat
Obesity
Metabolic syndrome
Residual glycaemic
Beta cell -PI
Beta cell +PI
Lipodystrophy
0.6
0.7
0.8
0.9
1.0
1.1
1.2
1.3
1.4
OR (95% CI) per 2-fold increase in genetically determined T2D risk
Approach to select T2D IVs
One variant per locus
Cluster-specific clumping
All T2D index variants

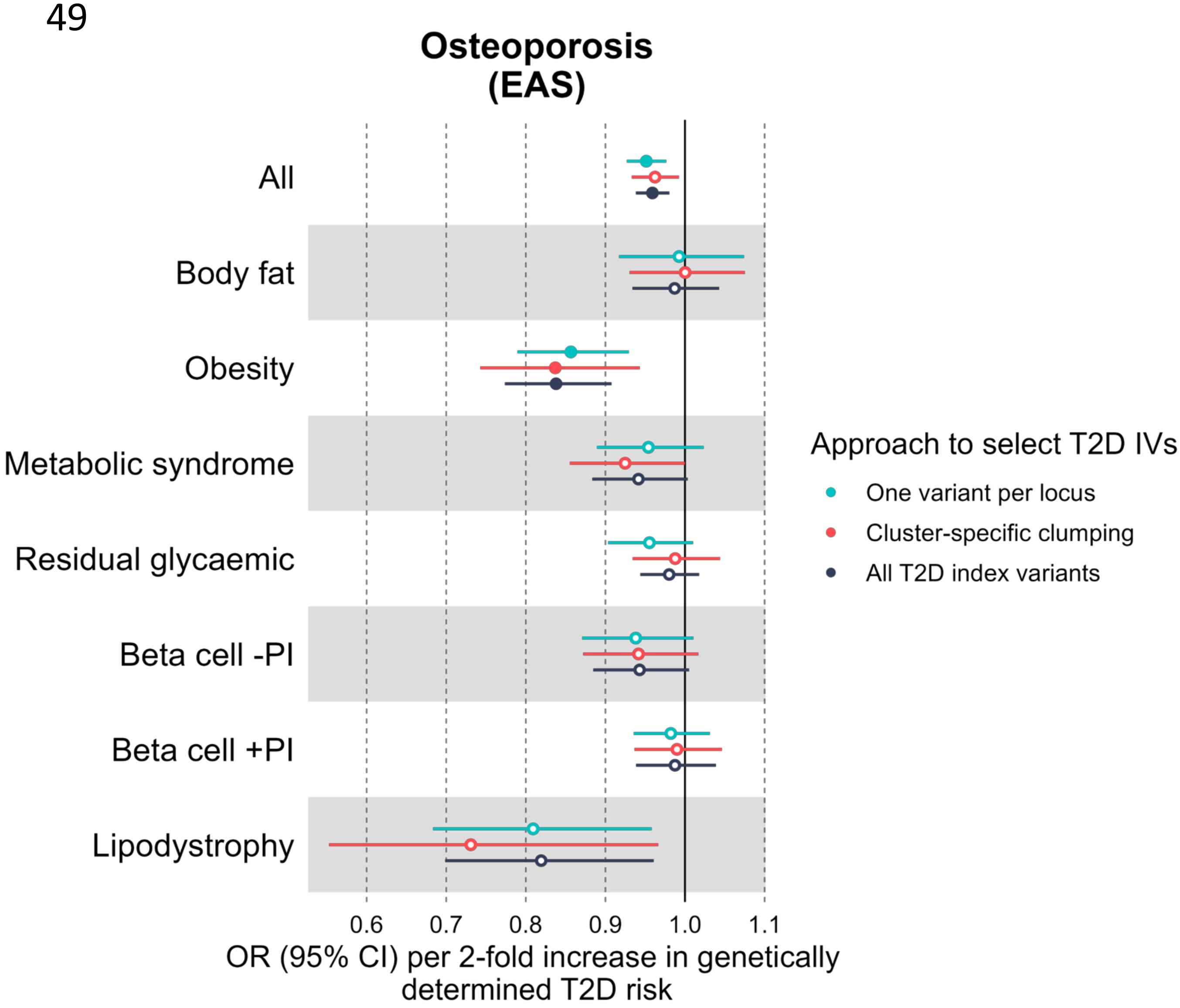
Osteoporosis
(EAS)
All
Body fat
Obesity
Metabolic syndrome
Residual glycaemic
Beta cell -PI
Beta cell +PI
Lipodystrophy
0.6
0.7
0.8
0.9
1.0
1.1
OR (95% CI) per 2-fold increase in genetically determined T2D risk
Approach to select T2D IVs
One variant per locus
Cluster-specific clumping
All T2D index variants

50

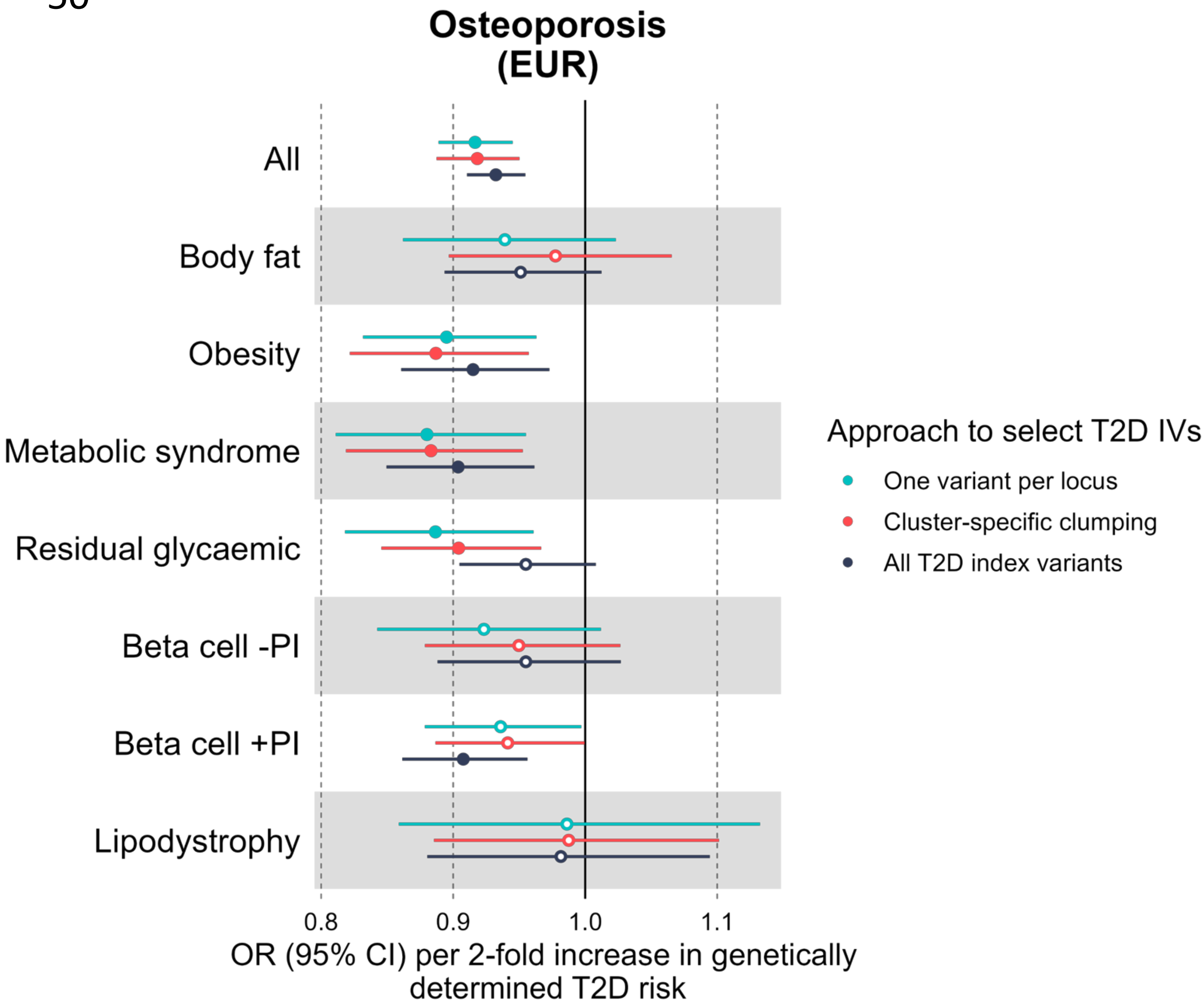

Osteoporosis
(EUR)
All
Body fat
Obesity
Metabolic syndrome
Residual glycaemic
Beta cell -PI
Beta cell +PI
Lipodystrophy
0.8
0.9
1.0
1.1
OR (95% CI) per 2-fold increase in genetically determined T2D risk
Approach to select T2D IVs
One variant per locus
Cluster-specific clumping
All T2D index variants

51

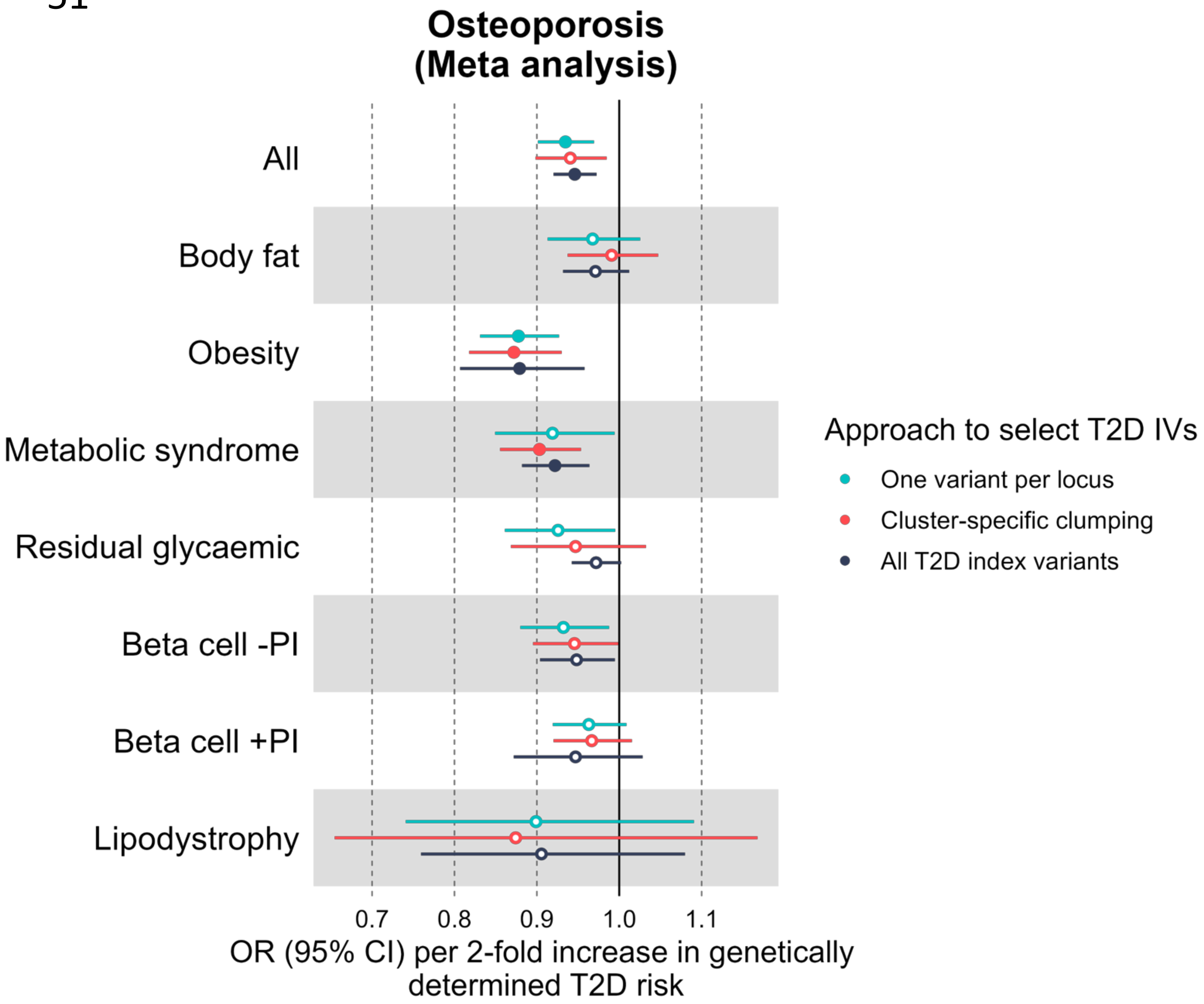
Osteoporosis
(Meta analysis)
All
Body fat
Obesity
Metabolic syndrome
Residual glycaemic
Beta cell -PI
Beta cell +PI
Lipodystrophy
0.7
0.8
0.9
1.0
1.1
OR (95% CI) per 2-fold increase in genetically determined T2D risk
Approach to select T2D IVs
One variant per locus
Cluster-specific clumping
All T2D index variants

52

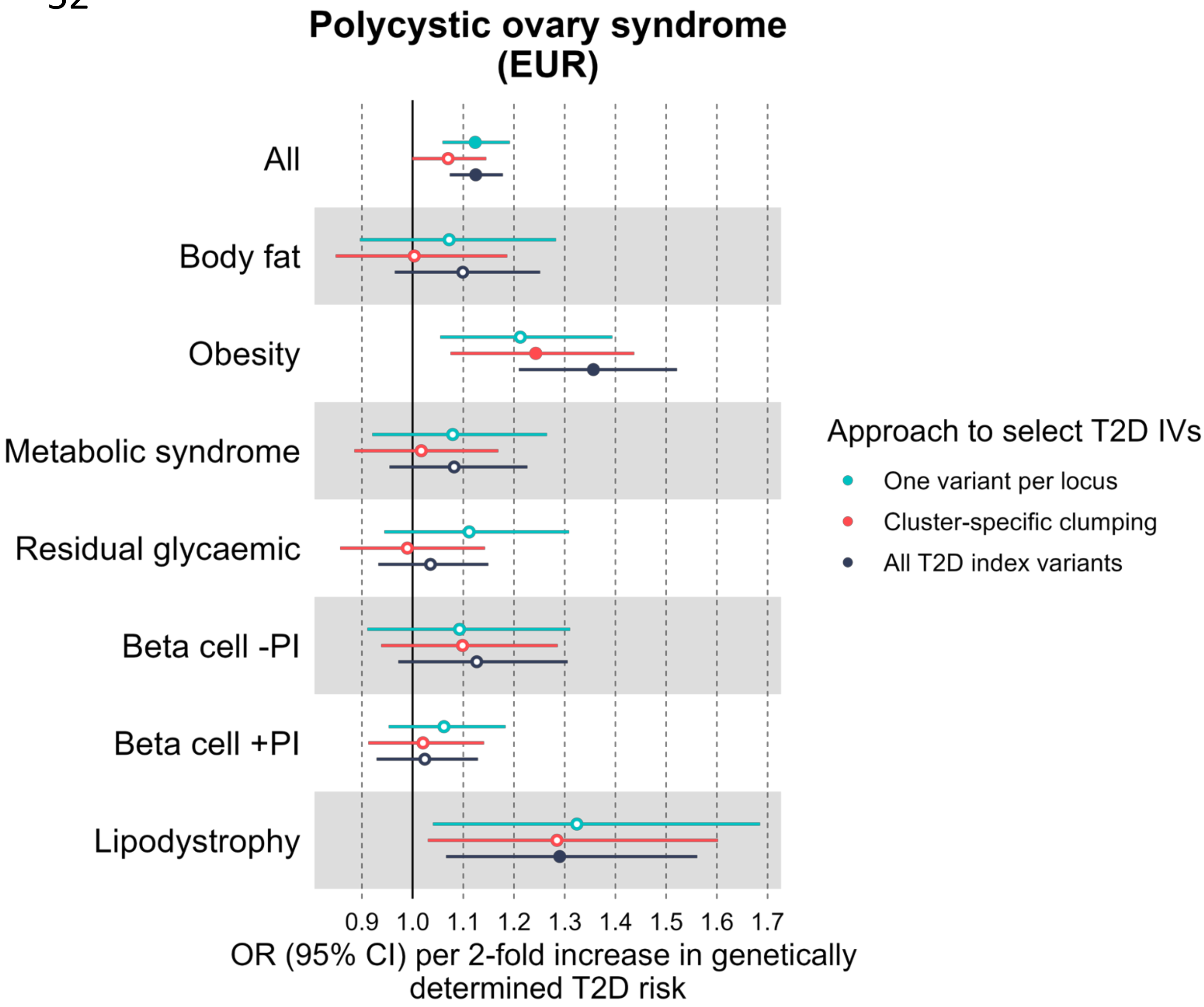
Polycystic ovary syndrome (EUR)
All
Body fat
Obesity
Metabolic syndrome
Residual glycaemic
Beta cell -PI
Beta cell +PI
Lipodystrophy
0.9 1.0 1.1 1.2 1.3 1.4 1.5 1.6 1.7
OR (95% CI) per 2-fold increase in genetically determined T2D risk
Approach to select T2D IVs
One variant per locus
Cluster-specific clumping
All T2D index variants

53

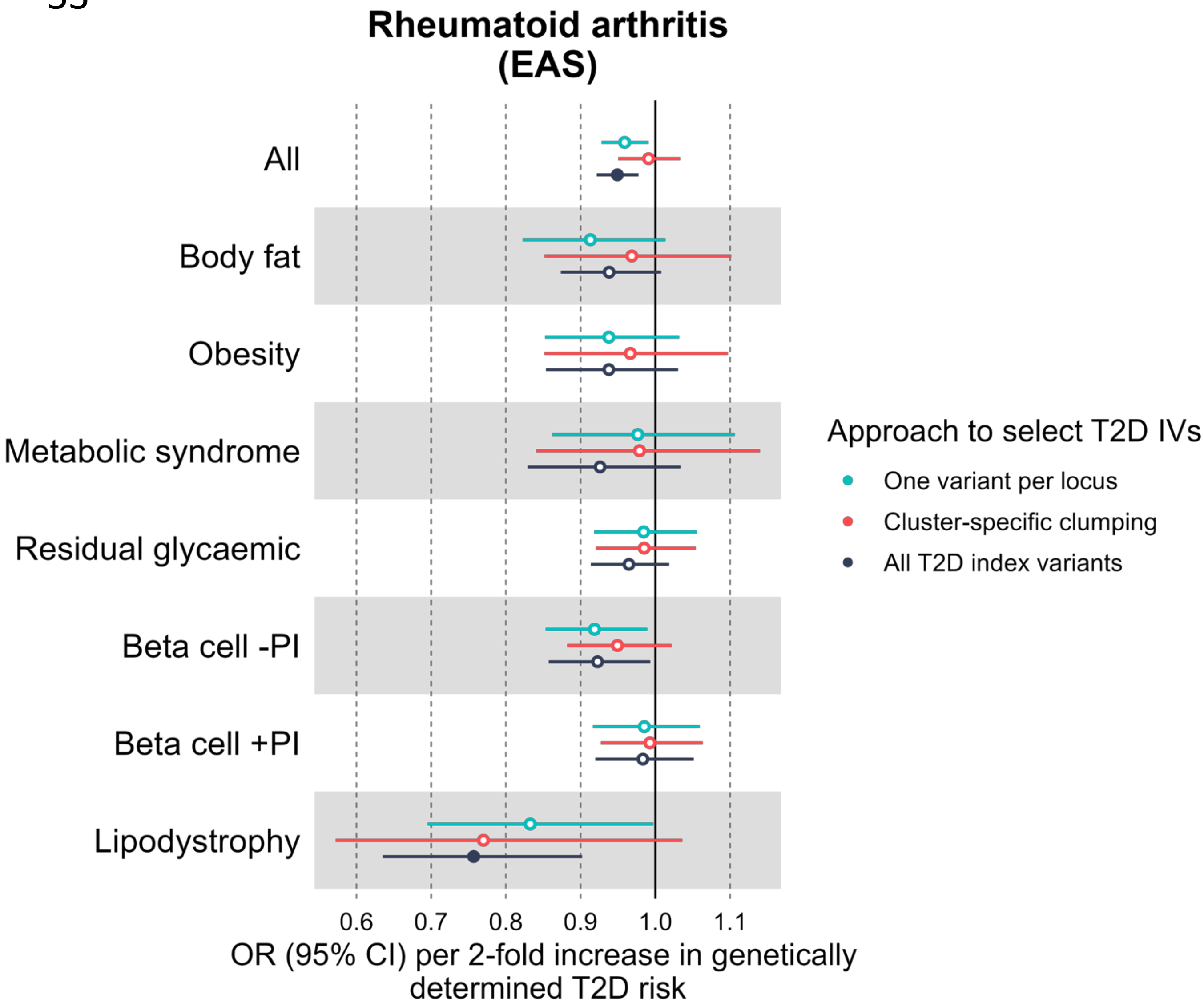
Rheumatoid arthritis
(EAS)
All
Body fat
Obesity
Metabolic syndrome
Residual glycaemic
Beta cell -PI
Beta cell +PI
Lipodystrophy
0.6
0.7
0.8
0.9
1.0
1.1
OR (95% CI) per 2-fold increase in genetically determined T2D risk
Approach to select T2D IVs
One variant per locus
Cluster-specific clumping
All T2D index variants

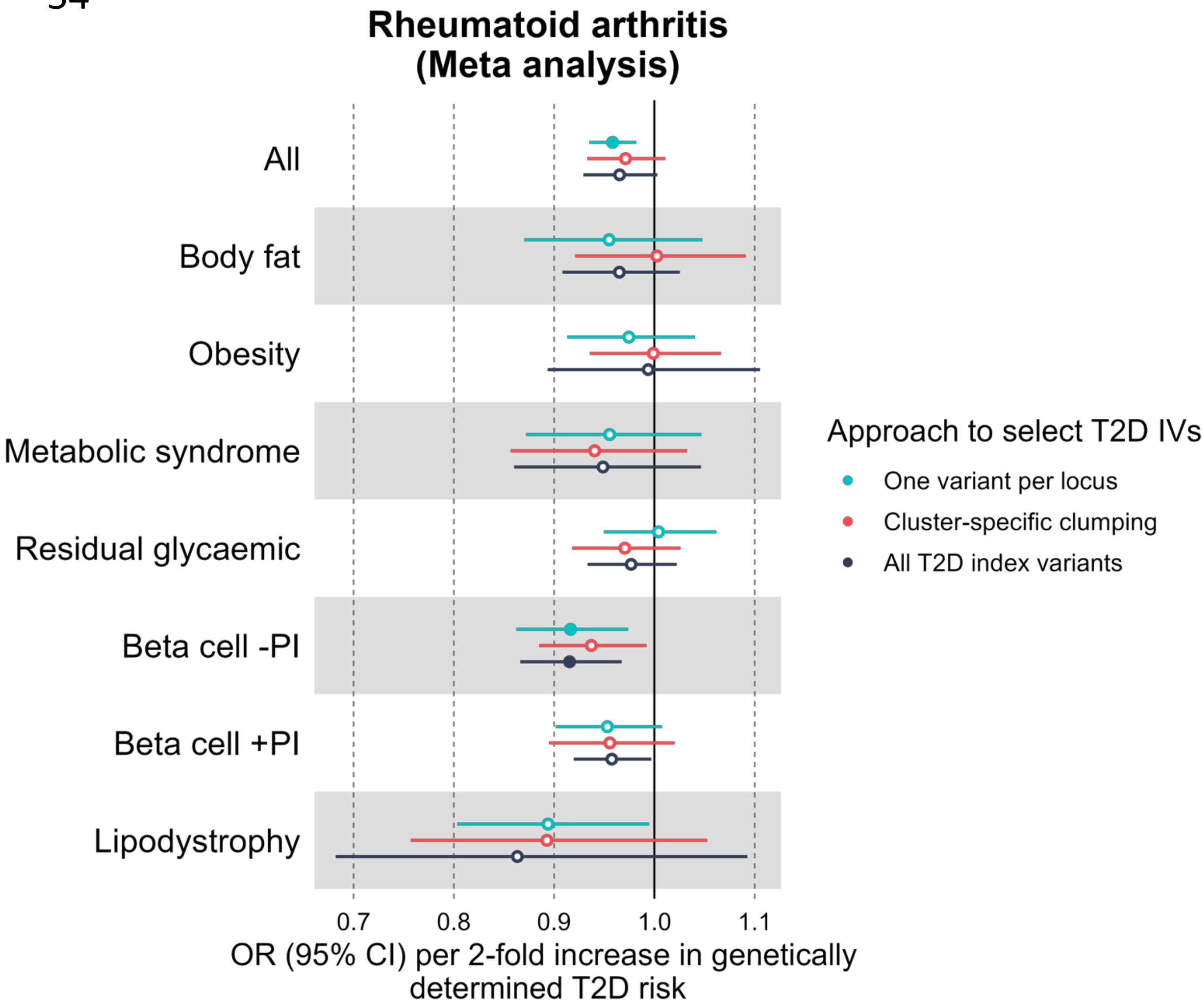
Rheumatoid arthritis
(Meta analysis)
All
Body fat
Obesity
Metabolic syndrome
Residual glycaemic
Beta cell -PI
Beta cell +PI
Lipodystrophy
0.7
0.8
0.9
1.0
1.1
OR (95% CI) per 2-fold increase in genetically determined T2D risk
Approach to select T2D IVs
One variant per locus
Cluster-specific clumping
All T2D index variants

55

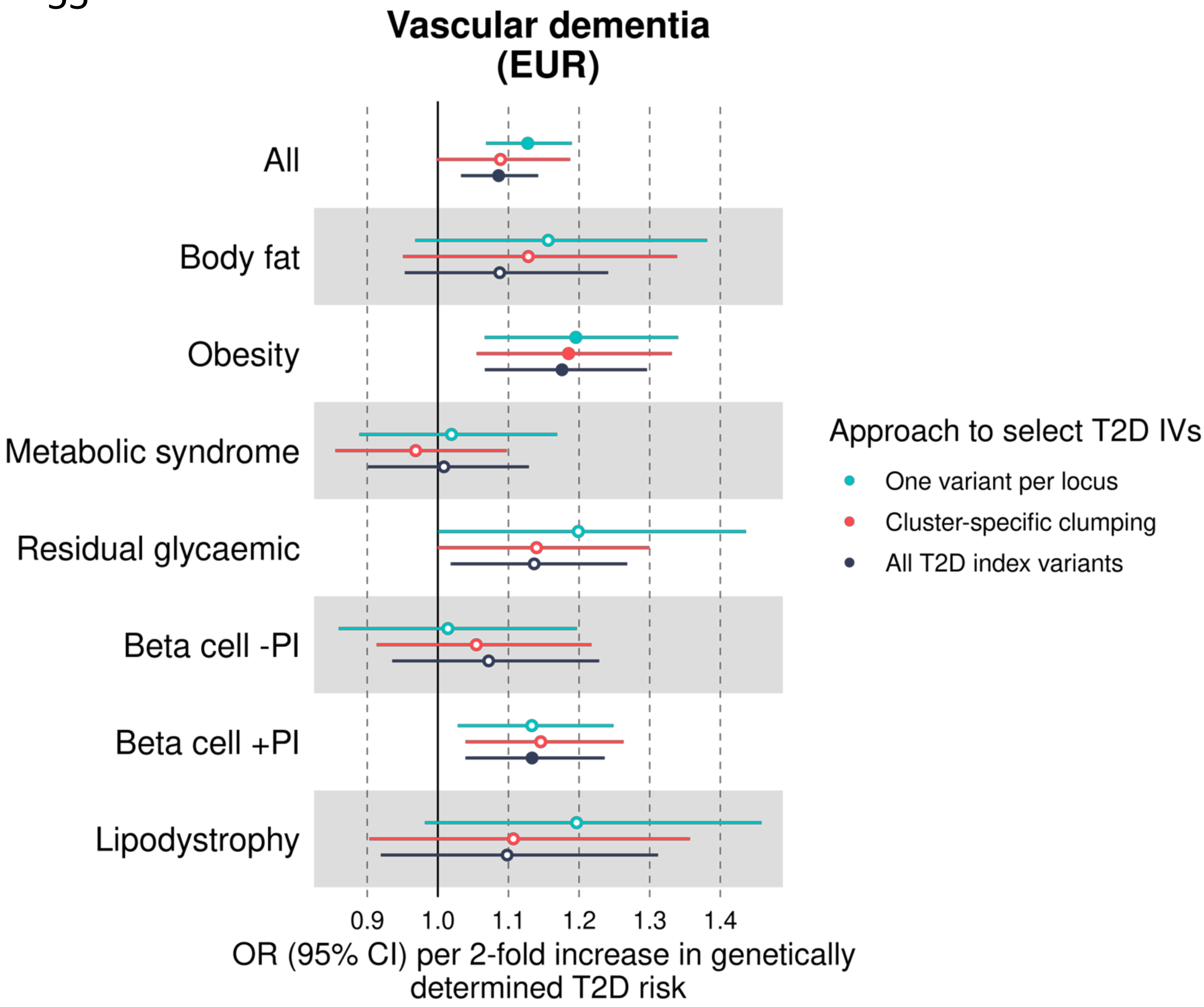

Vascular dementia
(EUR)
All
Body fat
Obesity
Metabolic syndrome
Residual glycaemic
Beta cell -PI
Beta cell +PI
Lipodystrophy
0.9
1.0
1.1
1.2
1.3
1.4
OR (95% CI) per 2-fold increase in genetically determined T2D risk
Approach to select T2D IVs
One variant per locus
Cluster-specific clumping
All T2D index variants

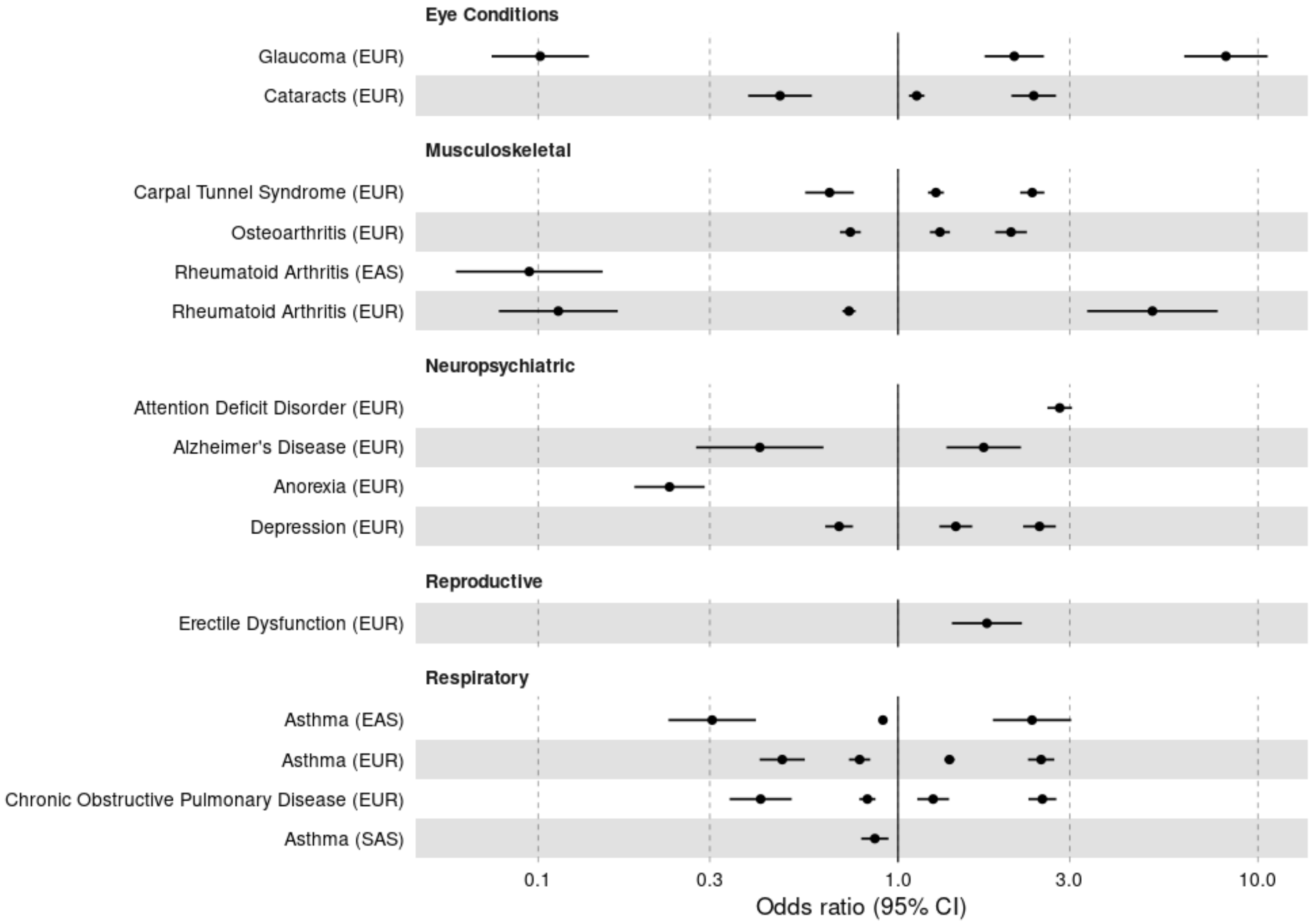

Supplementary Figure 56: MR-Clust results for the estimated cluster-stratified MR results significant after FDR correction. Each estimate corresponds to one cluster identified by MR-Clust.

Supplementary Figures 57-71: MR-Clust scatterplots of putative T2D causal clusters on non-cardiometabolic comorbidities. MR-Clust (V.0.1.0)[1] was used to identify putatively causal clusters of genetic predisposition to T2D as an exposure on non-cardiometabolic comorbidities as an outcome. We restricted putative causal clusters to instrumental variables (IVs) with a >80% probability of inclusion in the cluster, and removed any IVs placed in "Null" or "Junk" clusters by the MR-Clust algorithm. The genetic ancestry groups represent individuals genetically similar to Africans (AFR), East Asians (EAS), Europeans (EUR), admixed Americans (AMR) and South Asians (SAS) as defined by the 1000 Genomes Project. All scatterplots were generated using the "two_stage_plot" function implemented in MR-Clust. When possible, T2D IVs were annotated in the scatterplot by their cardiometabolic cluster from Suzuki et al[2].

Putative causal MR-Clust clusters of T2D genetic predisposition on carpal tunnel syndrome in the EUR ancestry group

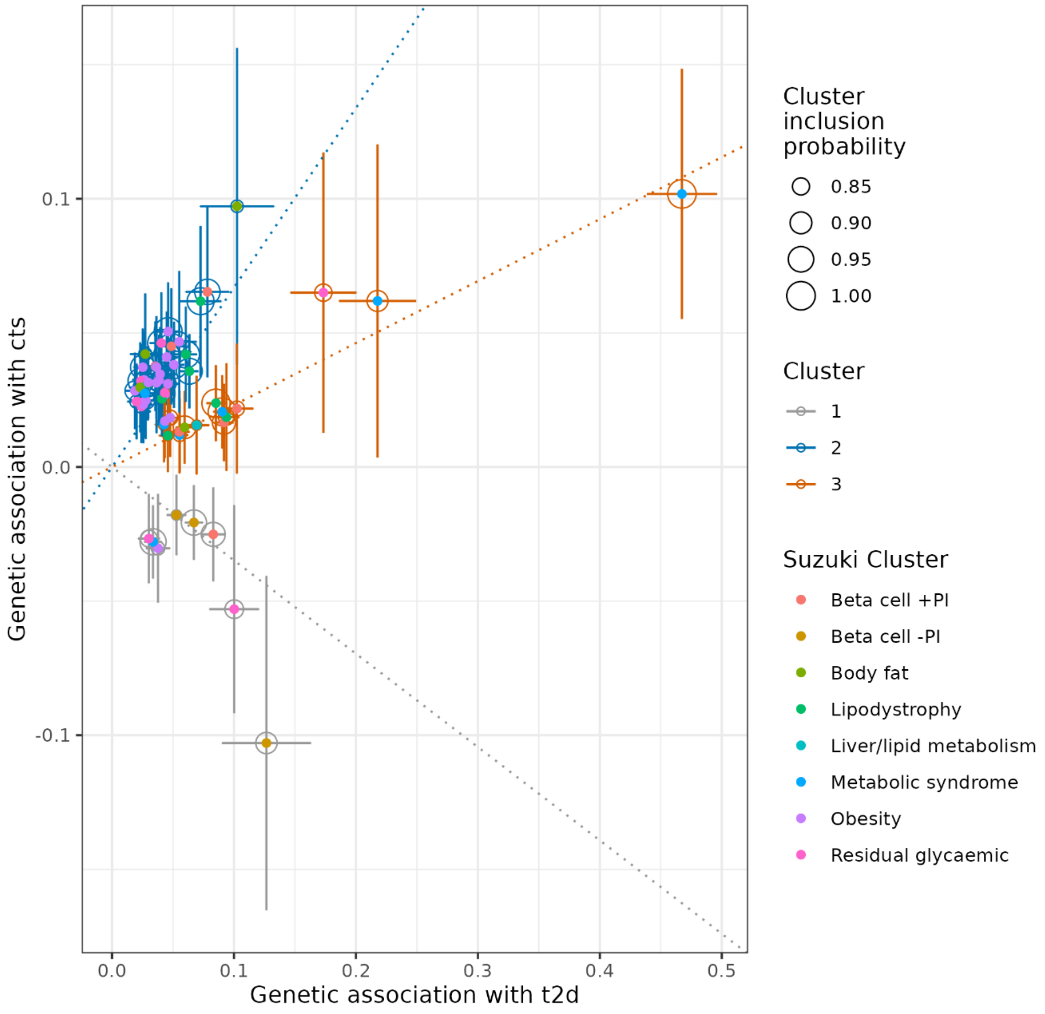

Putative causal MR-Clust clusters of T2D genetic predisposition on osteoarthritis in the EUR ancestry group

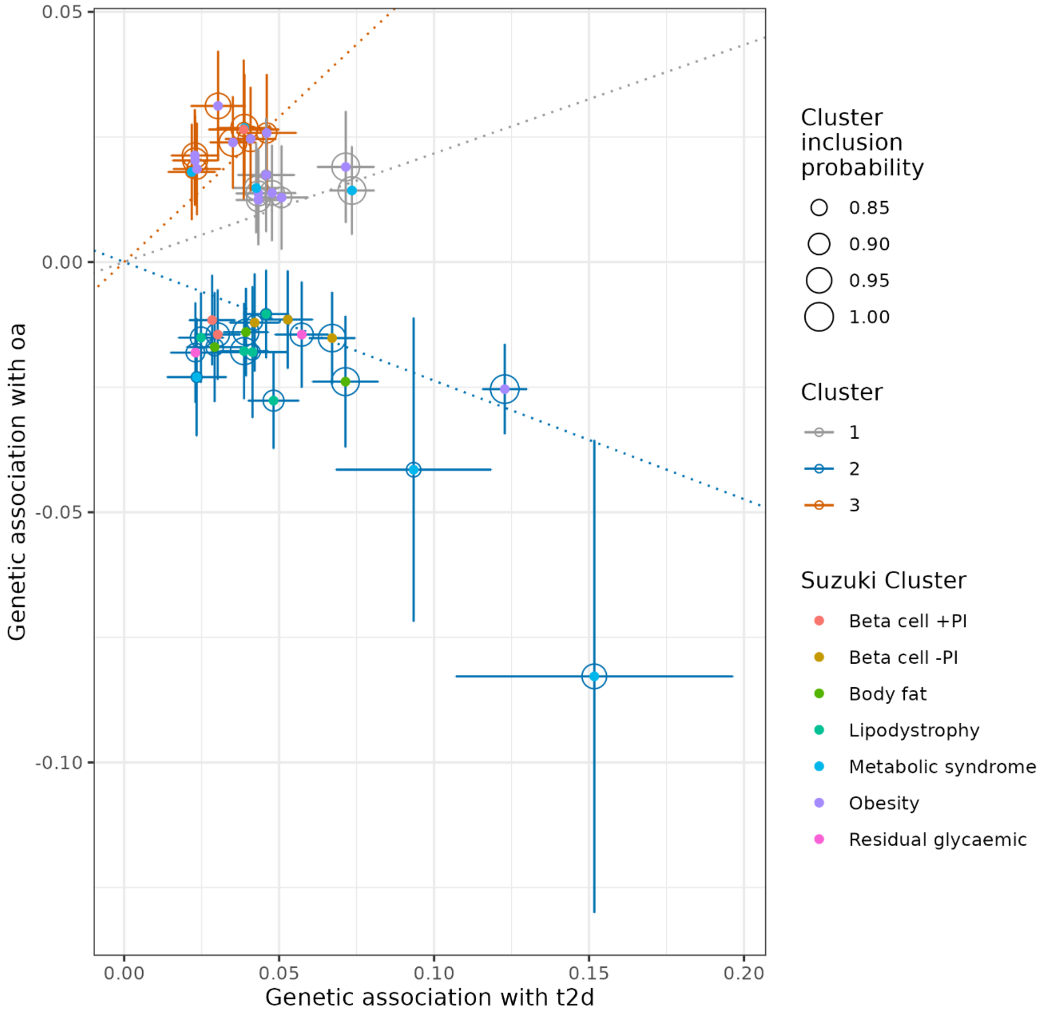

Putative causal MR-Clust clusters of T2D genetic predisposition on rheumatoid arthritis in the EUR ancestry group

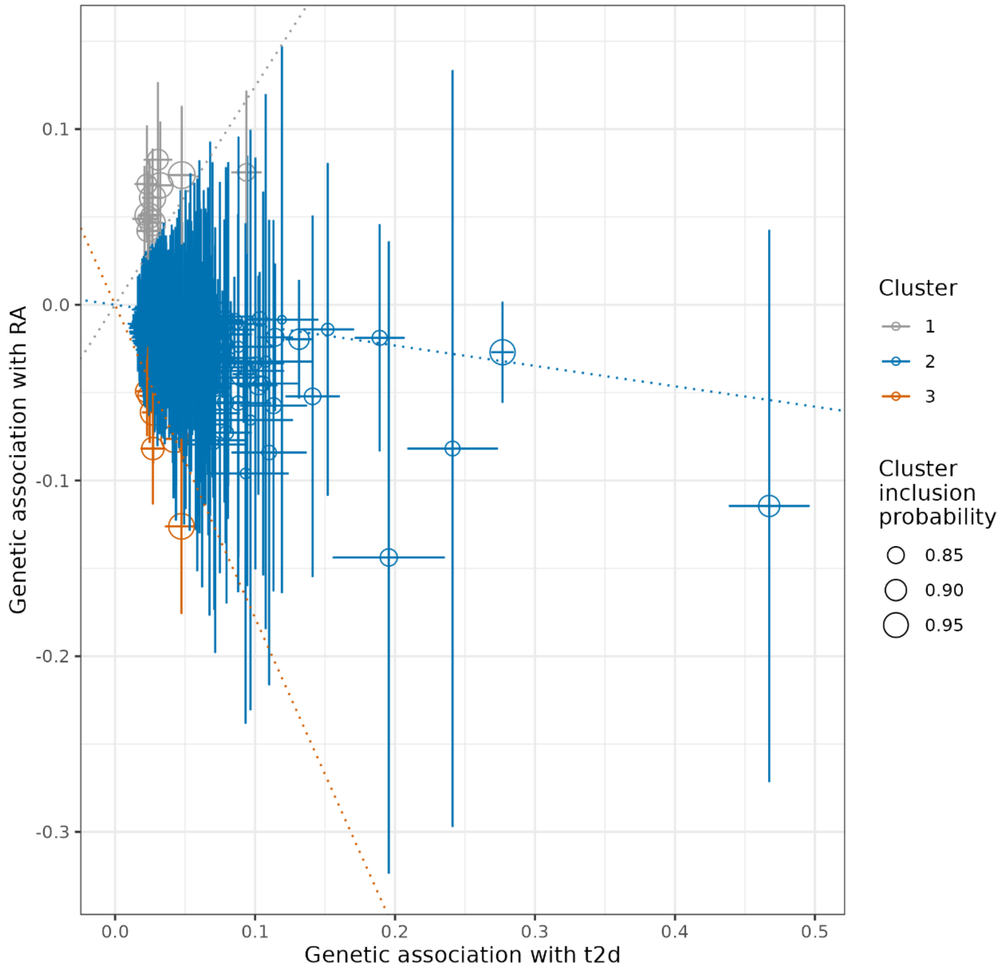

Putative causal MR-Clust clusters of T2D genetic predisposition on rheumatoid arthritis in the EAS ancestry group

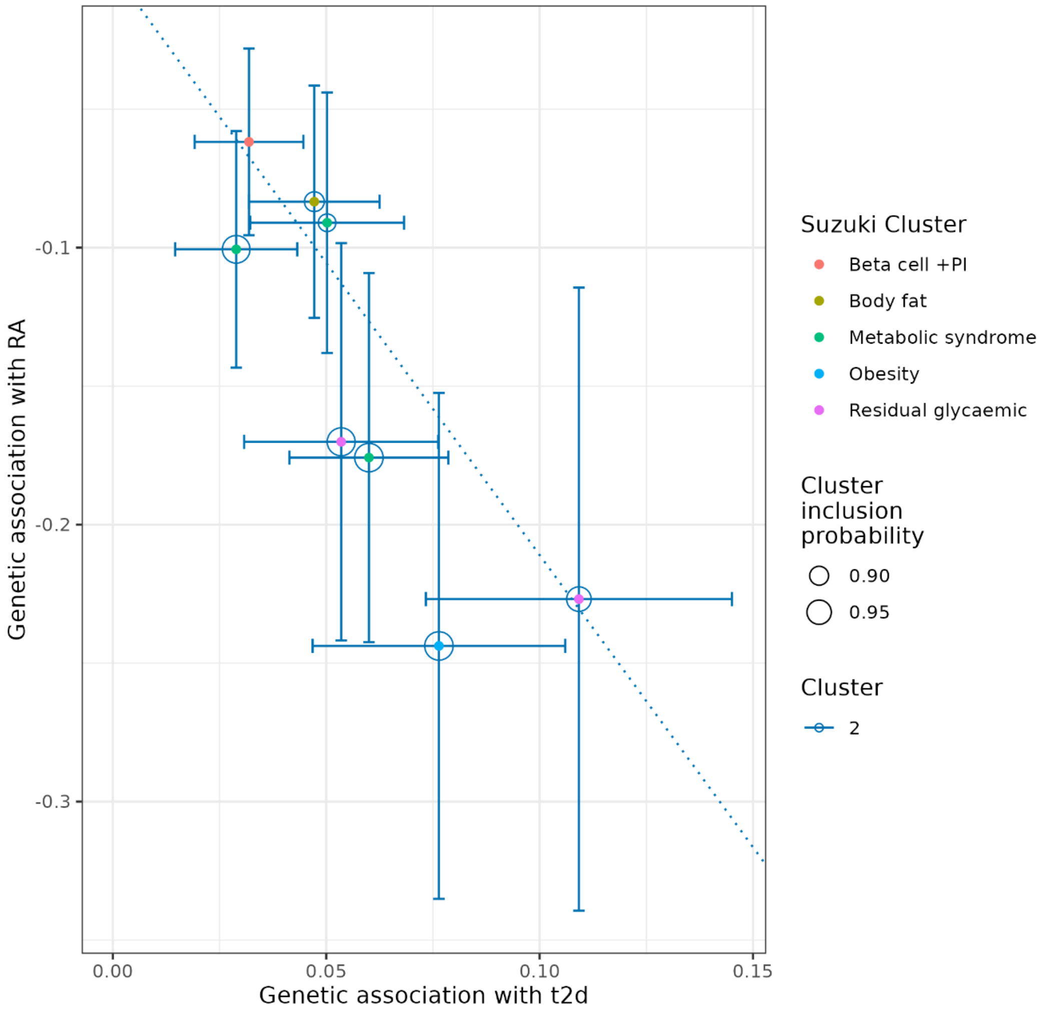

Putative causal MR-Clust clusters of T2D genetic predisposition on attention deficit disorder in the EUR ancestry group

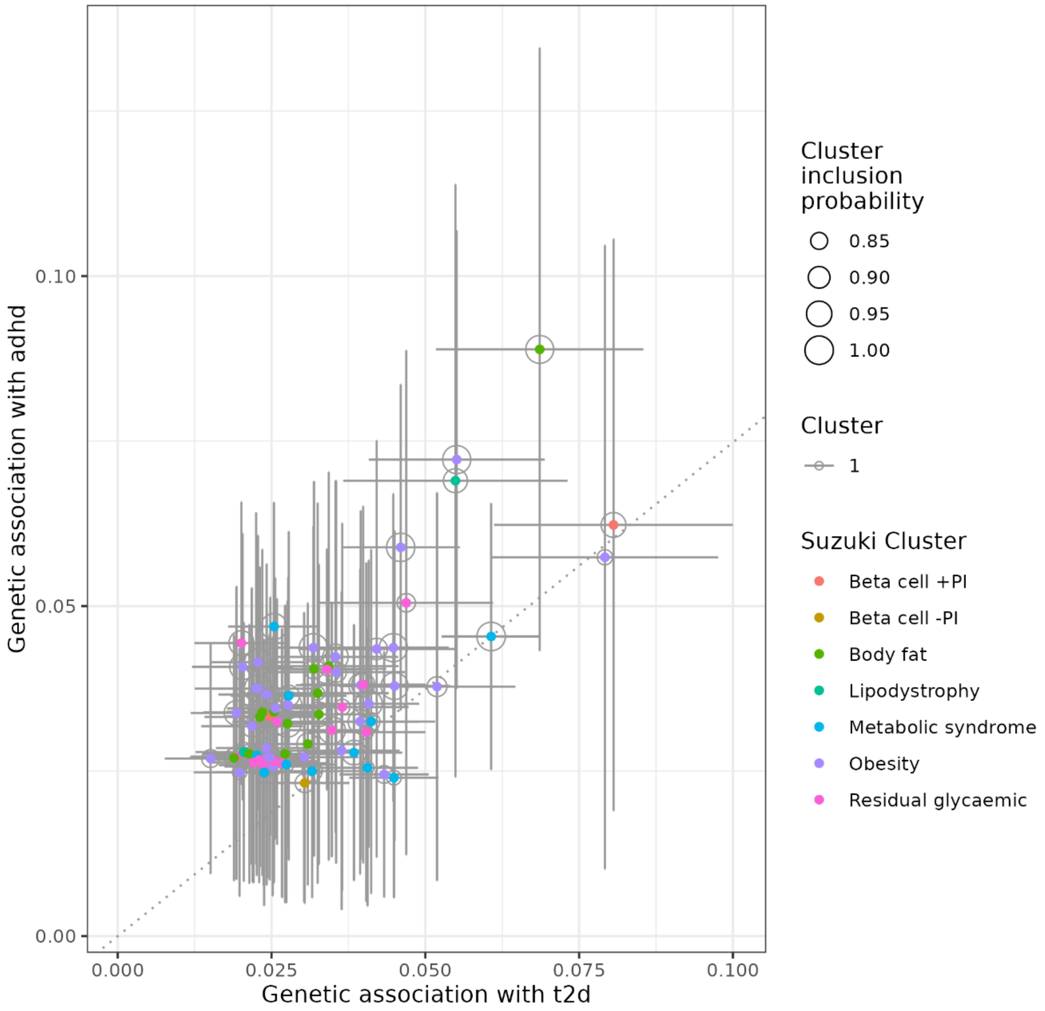

Putative causal MR-Clust clusters of T2D genetic predisposition on clinically diagnosed Alzheimer's disease in the EUR ancestry group

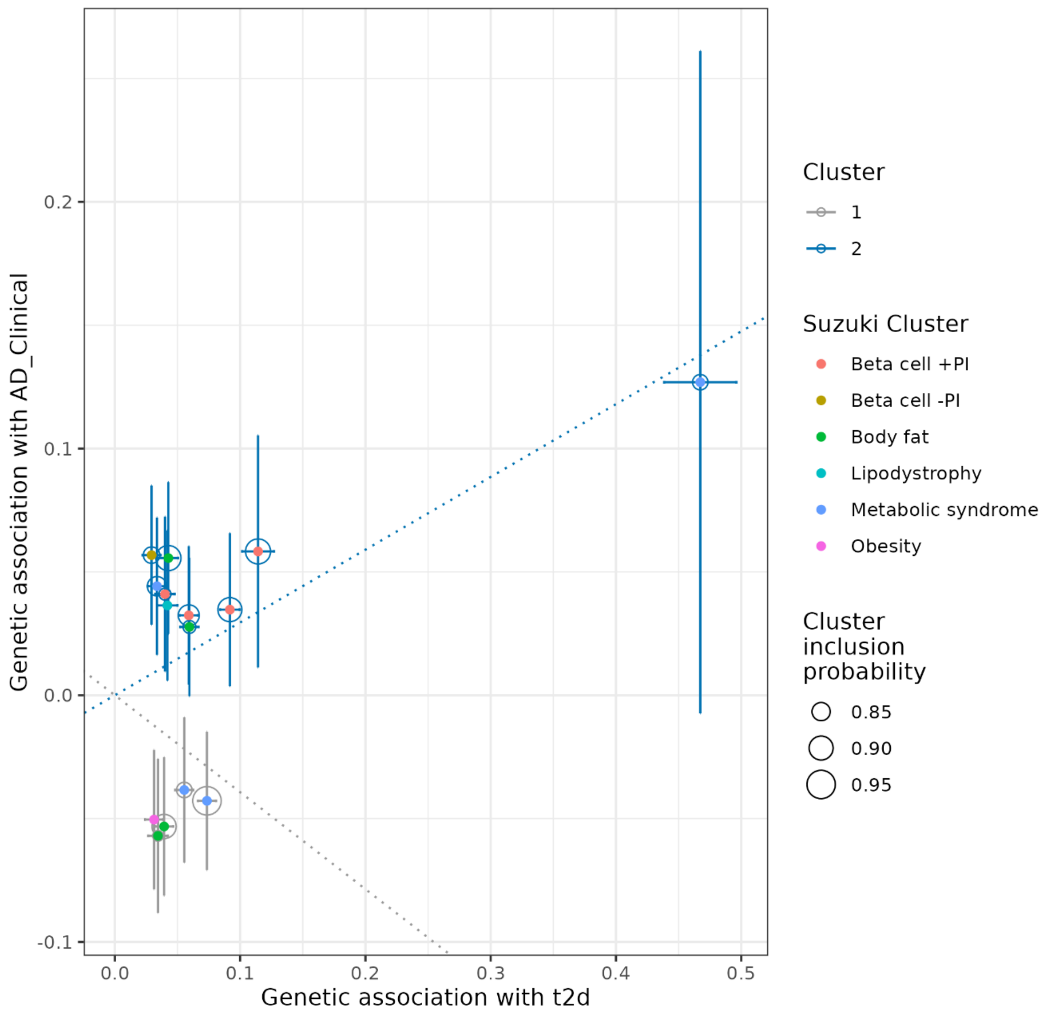

Putative causal MR-Clust clusters of T2D genetic predisposition on anorexia in the EUR ancestry group

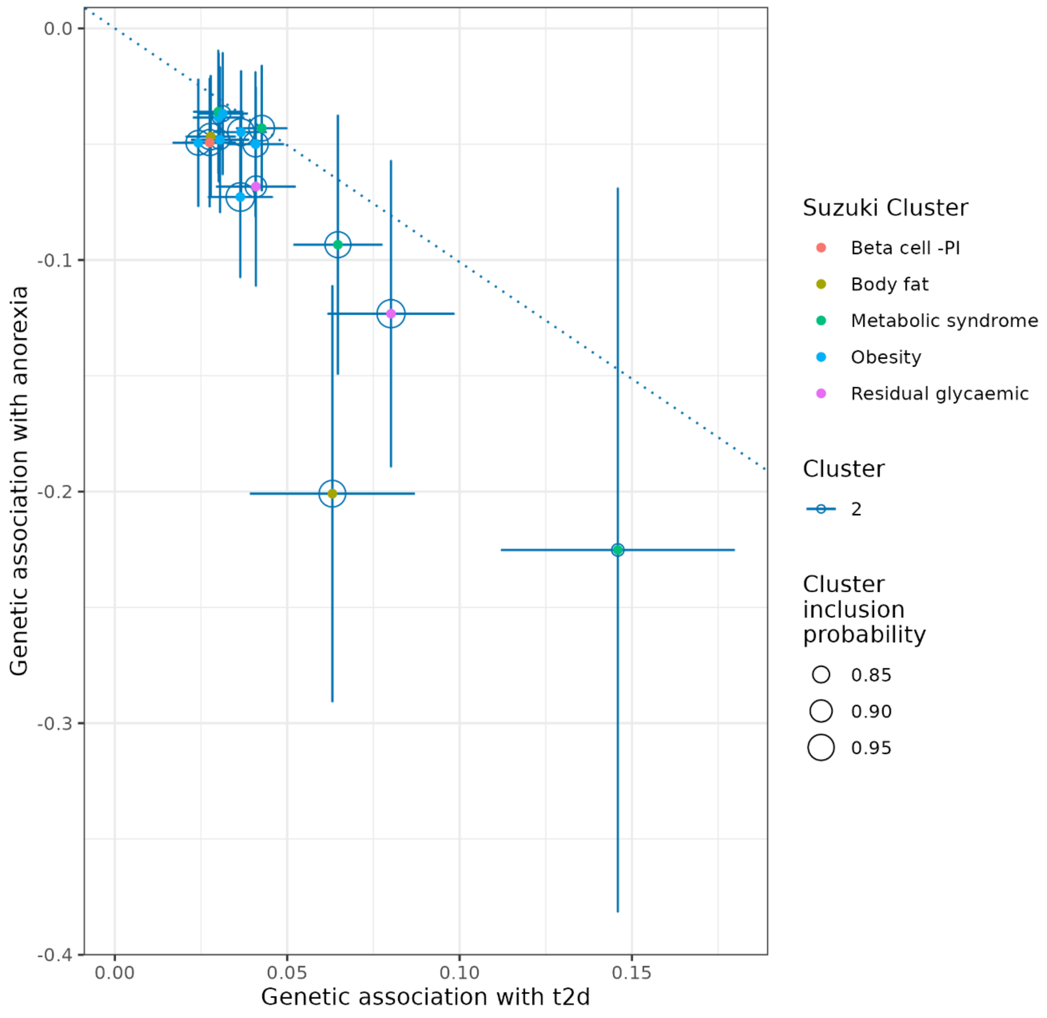

Putative causal MR-Clust clusters of T2D genetic predisposition on depression in the EUR ancestry group

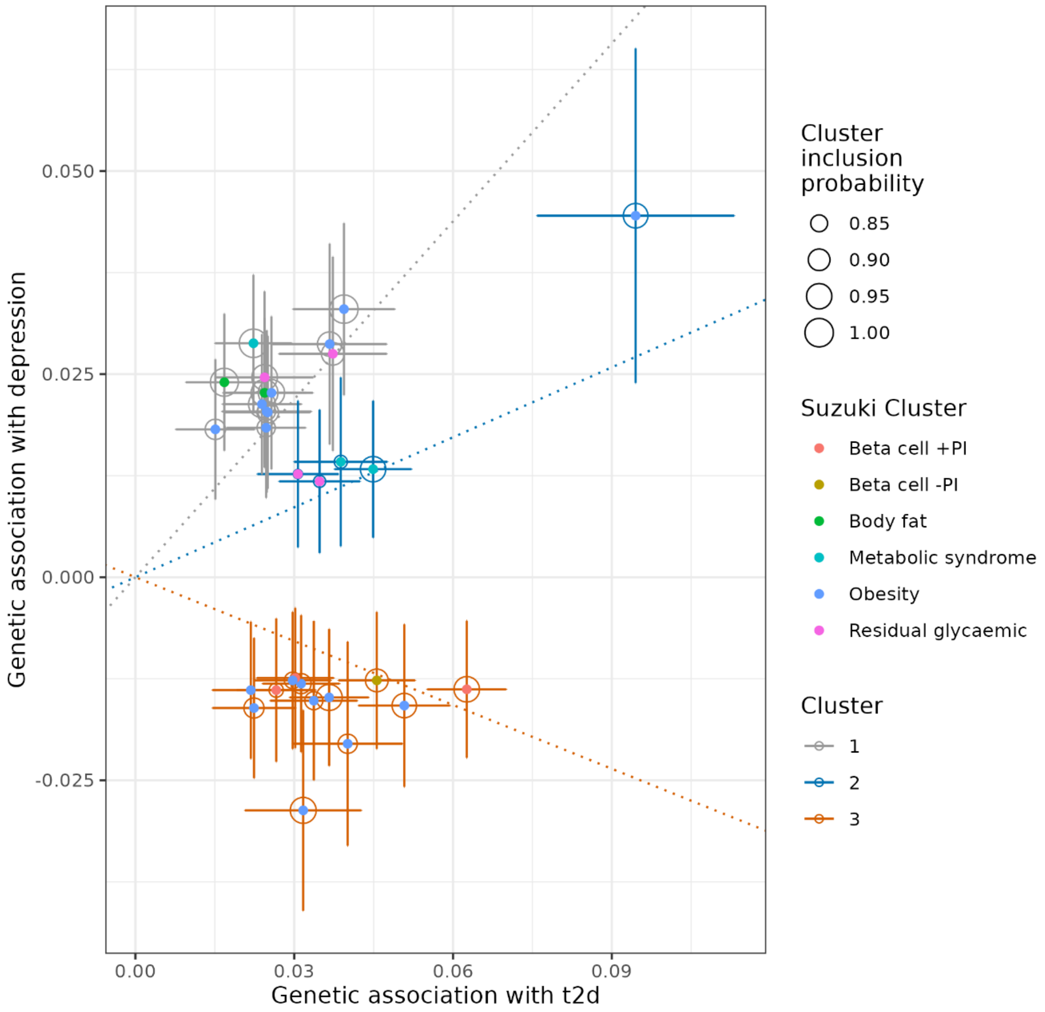

Putative causal MR-Clust clusters of T2D genetic predisposition on glaucoma in the EUR ancestry group

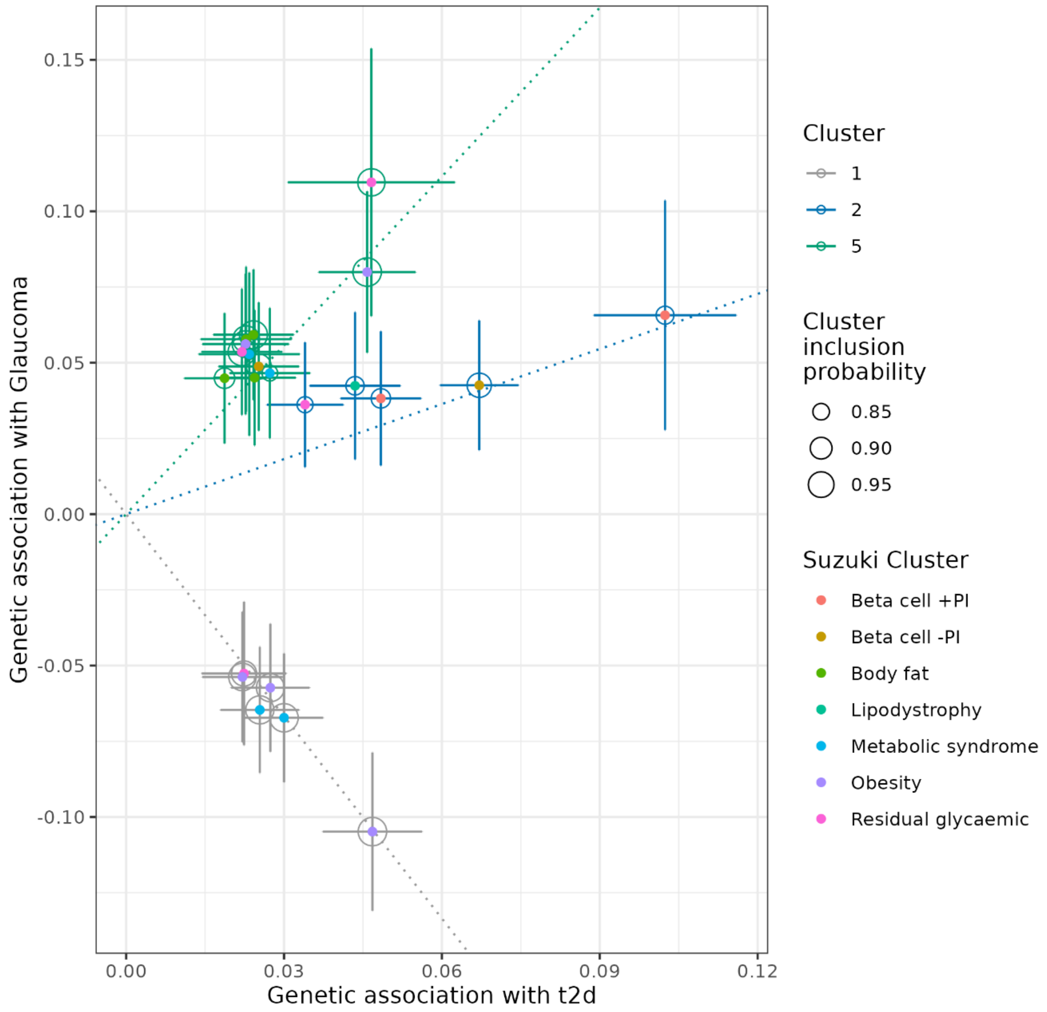

Putative causal MR-Clust clusters of T2D genetic predisposition on cataracts in the EUR ancestry group

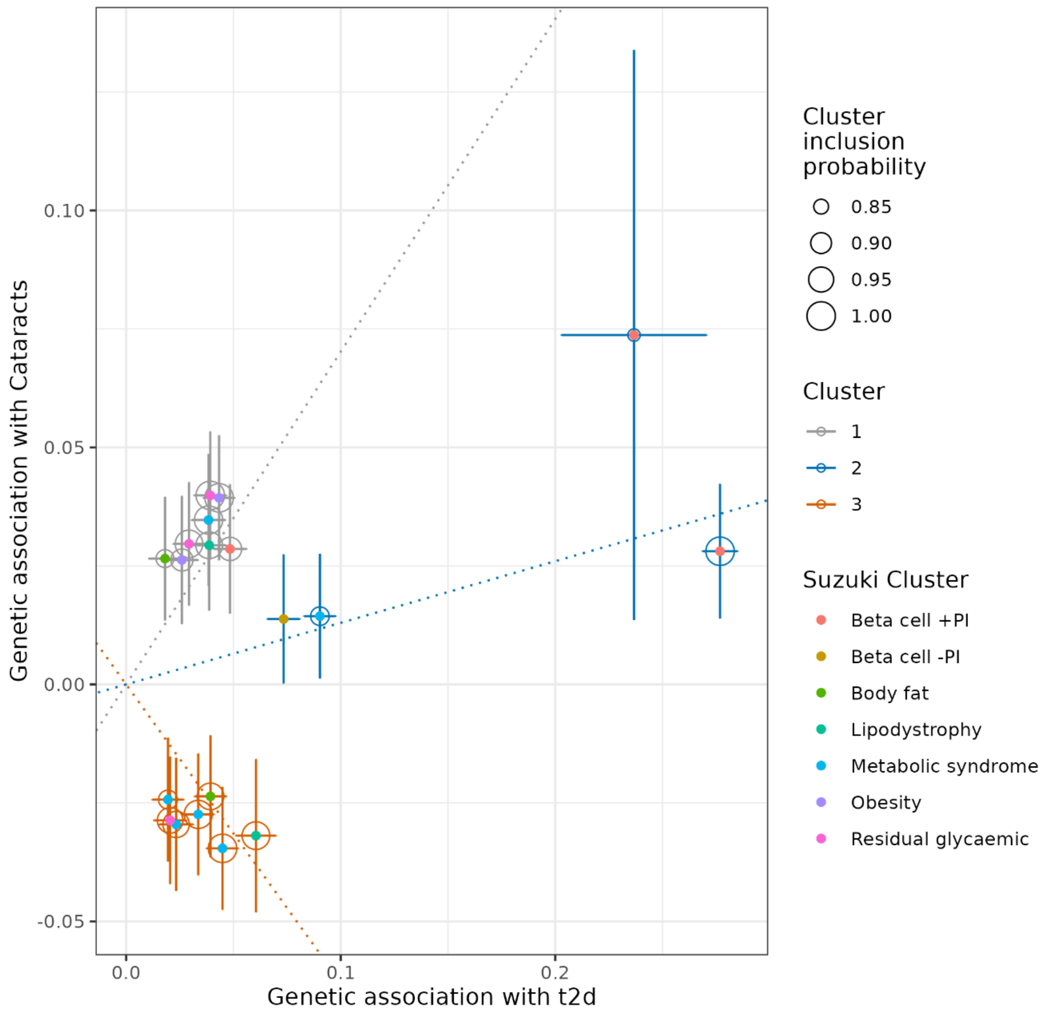

Putative causal MR-Clust clusters of T2D genetic predisposition on erectile dysfunction in the EUR ancestry group

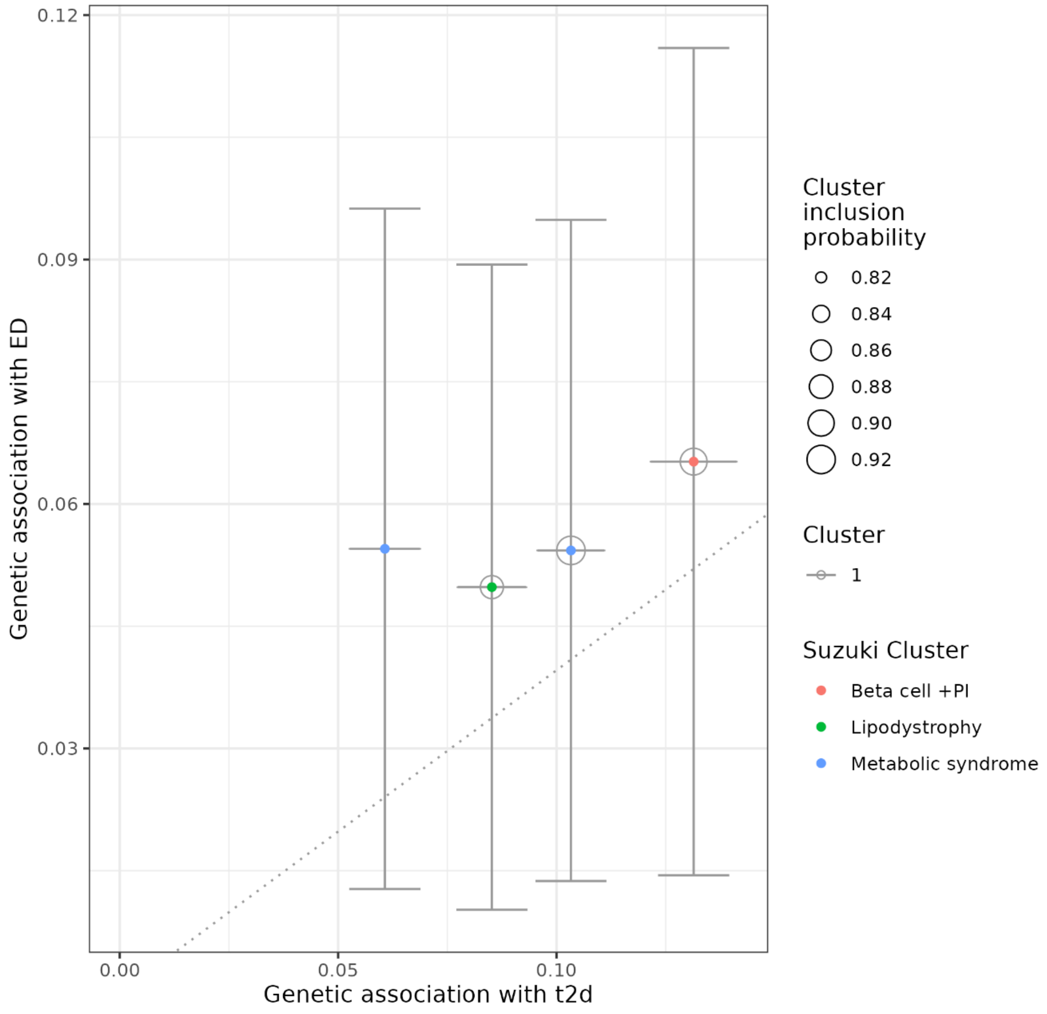

Putative causal MR-Clust clusters of T2D genetic predisposition on chronic obstructive pulmonary disease in the EUR ancestry group

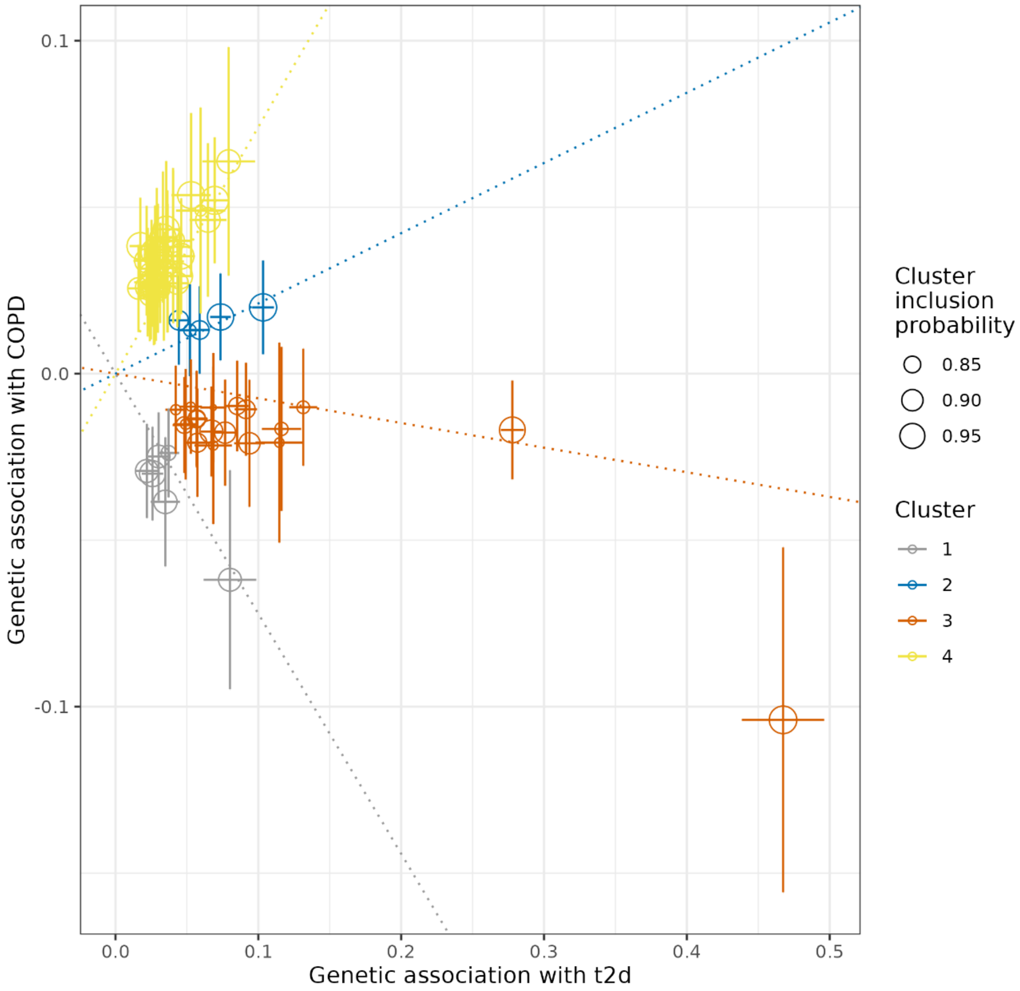

Putative causal MR-Clust clusters of T2D genetic predisposition on asthma in the EUR ancestry group

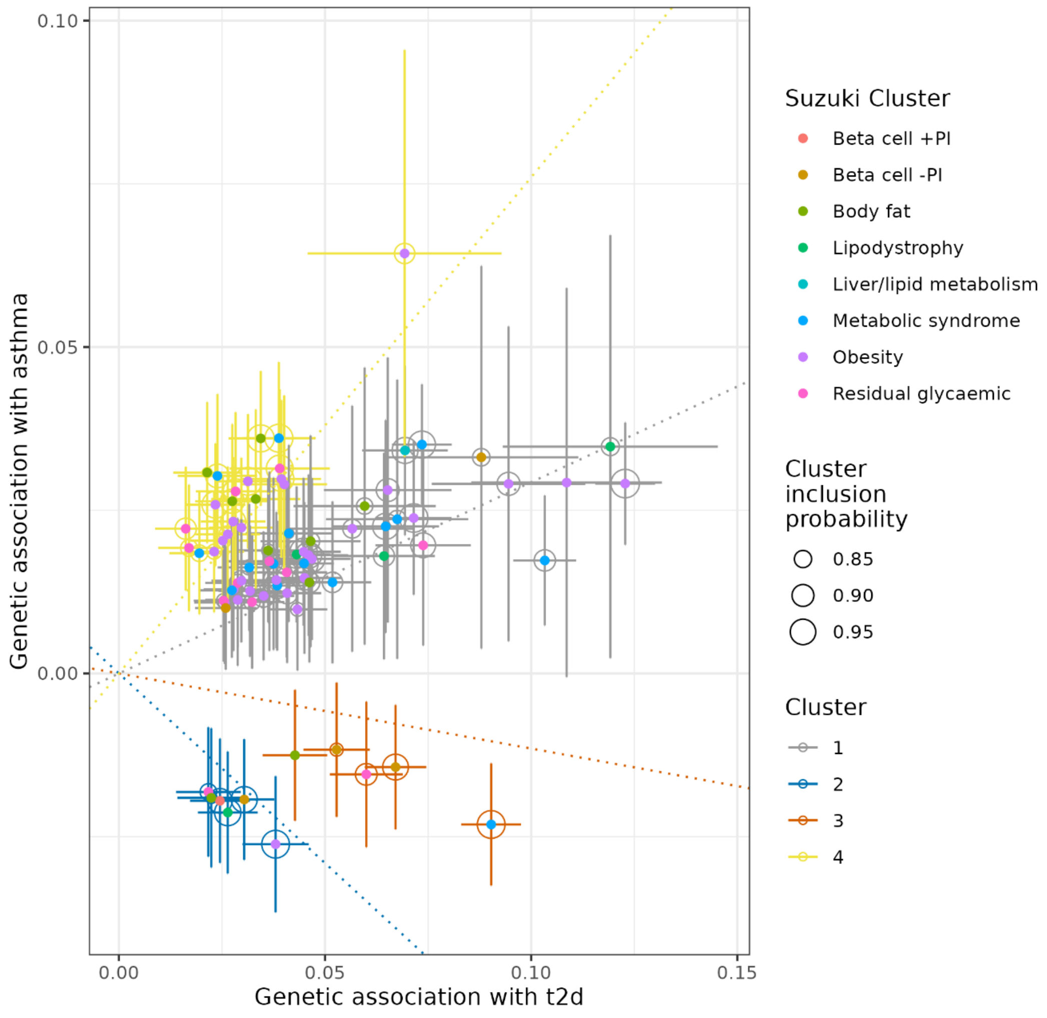

Putative causal MR-Clust clusters of T2D genetic predisposition on asthma in the EAS ancestry group

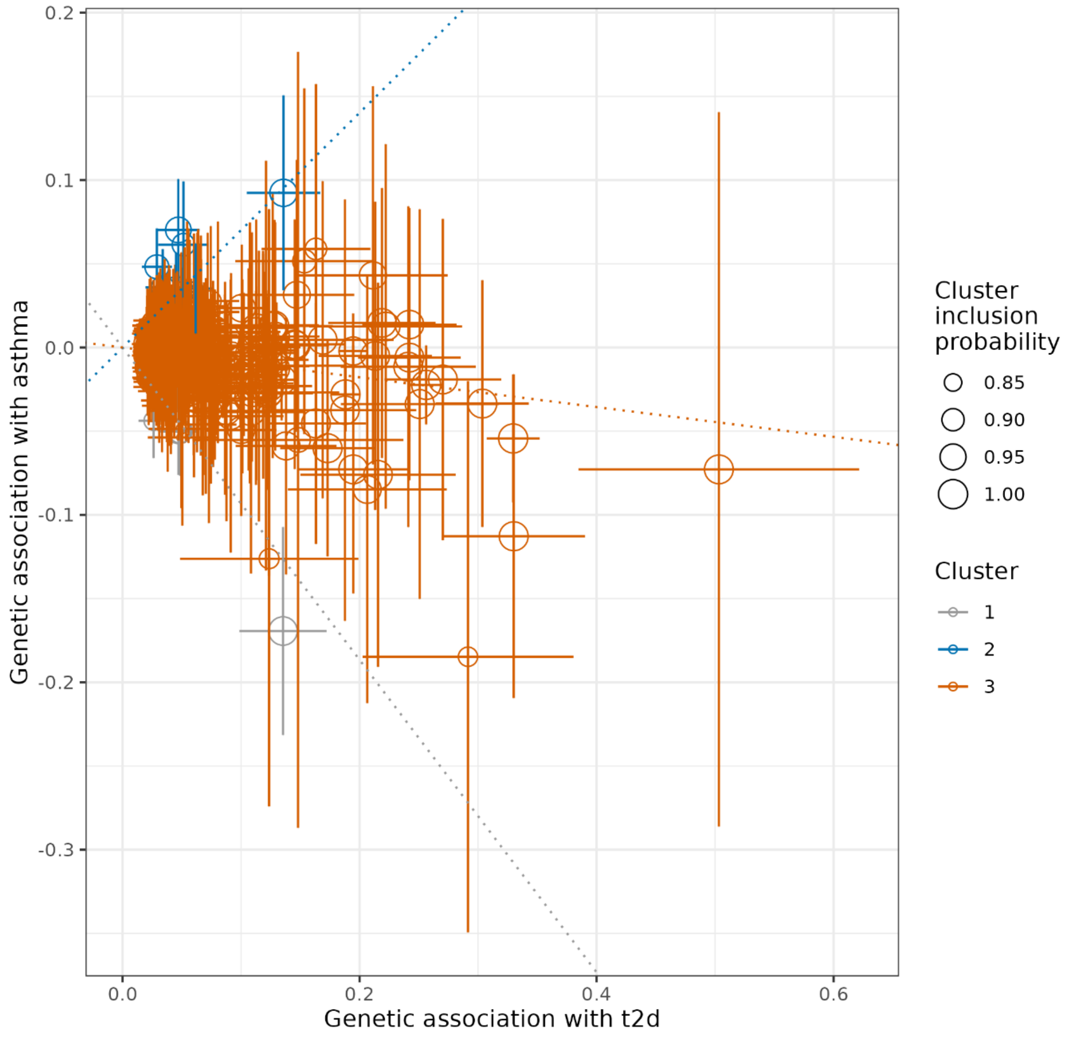

Putative causal MR-Clust clusters of T2D genetic predisposition on asthma in the SAS ancestry group

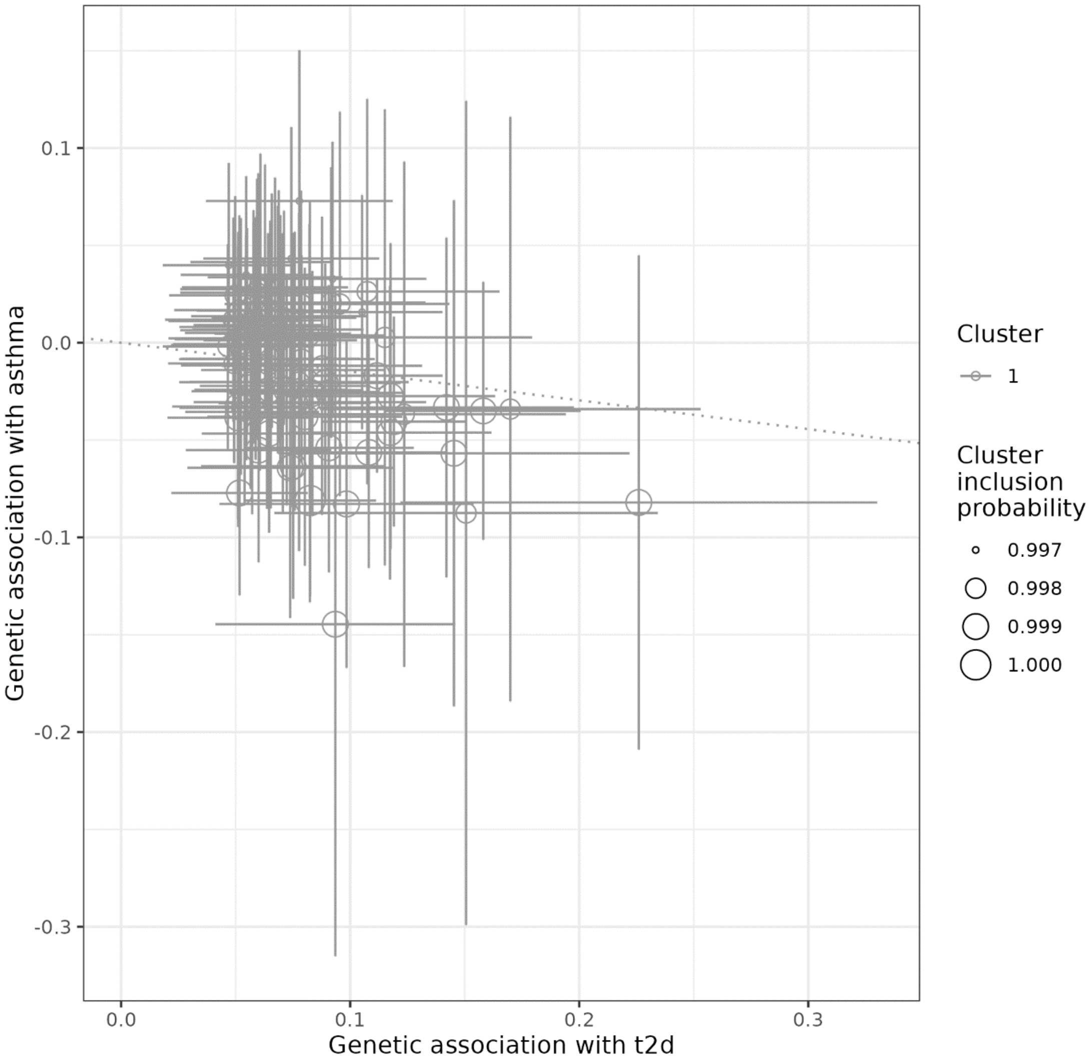

Supplementary Notes

Supplementary Note 1: Detailed description of the results of the sensitivity analysis to assess the validity of the forward Mendelian randomization assumptions for the statistically significant IVW results at an FDR of 5%.

We have detailed all sensitivity analyses we have performed in Supplementary Table 5.

- Direction of association:
    - Steiger-filter-based directionality test
    - Steiger-filtered IVW
    - Reverse MR
- i.i.d. assumption of genetic instrumental variables (IVs):
    - Different set of T2D IVs: we compared the effect magnitude of our results with alternative approaches to select T2D IVs. We have employed three additional approaches to define T2D IVs: selecting one variant per locus or clumping the independent risk variants either all at once or per cluster
    - Correlated IVW (need FDR-adjusted p-value < 0.05)
- Inverse cluster-stratified MR analysis (Leave-One-Cluster-Out Analysis)

Additional analyses to triangulate evidence of the Mendelian randomization analyses

- PheWAS
- MR-Clust

We describe attenuation as an adjusted IVW estimate that is no longer statistically significant at FDR of 5%. Mutual attenuation describes the case where both exposure estimates are no longer statistically significant at FDR of 5% after the adjustment. Mutual attenuation suggests a shared etiology. The results of the multivariable MR analysis can be found in Supplementary Data 5, the results the sensitivity analyses that assess the validity of the exclusion-restriction criteria can be found in Supplementary Data 1 and Supplementary Data 7, and the results of the Steiger-filter directionality test can be found in Supplementary Data 10.

Osteoarthritis

For osteoarthritis, we find evidence of a risk-increasing causal effect of T2D genetic predisposition and genetic risk linked to the obesity cluster. In contrast, T2D genetic predisposition linked to both beta cell clusters is protective to osteoarthritis. We adjust all estimates for cardiometabolic traits that show a statistically significant IVW effect after FDR correction at 5%, namely body mass index (BMI), waist-to-hip ratio (WHR), and subcutaneous adipose tissue (SAT) volume.

All:

- Statistical evidence of horizontal pleiotropy
- Statistical evidence of heterogeneity
- Different directions between MR methods: MR-Egger, penalized weighted median, weighted median, and weighted mode
- Changes direction but remains significant after adjustment for BMI
- No longer significant after adjusting for WHR
- Mutual attenuation: SAT volume

Obesity:

- Statistical evidence of heterogeneity
- Changes direction but remains significant after adjustment for BMI
- No longer significant after adjusting for BMI
- T2D attenuates the effect of WHR

Beta cell +PI/-PI:

- No longer significant after adjusting for BMI, WHR and SAT volume

Carpal tunnel syndrome (CTS)

We either observe no or mutual attenuation for all estimates of T2D genetic predisposition on CTS upon adjustment for cardiometabolic traits.

All:

- Statistical evidence of horizontal pleiotropy
- Statistical evidence of heterogeneity
- T2D attenuates the effect of fasting glucose (FG) (changes direction), random glucose (RG) and triglycerides (TG)
- Steiger-filter directionality test: incorrect direction

Obesity:

- Statistical evidence of horizontal pleiotropy
- Statistical evidence of heterogeneity
- T2D attenuates the effect of FG (changes direction), RG and TG

Body fat:

- Statistical evidence of heterogeneity
- Attenuated by BMI
- Mutual attenuation with FG, RG
- T2D attenuates the effect of TG

Lipodystrophy:

- Statistical evidence of heterogeneity
- T2D attenuates the effect of TG, FG
- Mutual attenuation with RG

Metabolic syndrome:

- T2D attenuates the effect of TG, FG
- Mutual attenuation with RG

Residual glycaemic:

- No longer significant after adjusting for BMI
- T2D attenuates the effect of TG, FG
- Mutual attenuation with RG

Back pain

The risk-increasing effect of T2D genetic predisposition linked to the obesity cluster on chronic back pain attenuates to zero upon adjustment for BMI and shows mutual attenuation when controlling for WHR, suggesting no direct effect of T2D on disease and shared etiology via obesity-related measures.

Obesity:

- Different directions between MR methods: Steiger IVW, MR-Egger, weighted mode
- Statistical evidence of horizontal pleiotropy
- No longer significant after adjusting for BMI
- Mutual attenuation with WHR

Osteoporosis

Individuals genetically similar to Europeans:

- All:
  - Statistical evidence of horizontal pleiotropy
  - T2D attenuates the effect of BMI
- Beta cell +PI: T2D attenuates the effect of BMI
- Obesity: T2D attenuates the effect of BMI
- MetSyn: T2D attenuates the effect of BMI

Meta-analysis across genetic similarity groups:

- All: statistical evidence of heterogeneity
- Obesity: statistical evidence of heterogeneity

Rheumatoid analysis

All (Individuals genetically similar to East Asians):

- Different directions between MR methods: MR-Egger
- Statistical evidence of horizontal pleiotropy

ADHD

All:

- Statistical evidence of horizontal pleiotropy

Obesity:

- Statistical evidence of heterogeneity
- No longer significant after adjusting for BMI
- T2D attenuates the effect of WHR

Body fat:

- No longer significant after adjusting for BMI

Metabolic syndrome:

- T2D attenuates the effect of WHR

Alzheimer's disease

Body fat:

- Changes direction but remains significant after adjustment for high-density lipoprotein (HDL) cholesterol levels

Anorexia nervosa

We identify a robust causal association between overall T2D genetic predisposition and decreased anorexia nervosa risk without any evidence of a cluster-stratified effect.

All:

- No longer significant after adjusting for BMI or WHR

OCD

We find evidence of reverse causation for all clusters, estimated by the Steiger directionality test. However, we do not find a statistically significant effect at FDR 5% of genetic predisposition to OCD on T2D risk.

All:

- No longer significant after adjusting for BMI
- T2D attenuates the effect of WHR
- Steiger-filter directionality test: incorrect direction

Obesity:

- No longer significant after adjusting for BMI and WHR
- Steiger-filter directionality test: incorrect direction

Residual glycaemic:

- No longer significant after adjusting for BMI
- Mutual attenuation with WHR
- Steiger-filter directionality test: incorrect direction

Glaucoma

All:

- Statistical evidence of heterogeneity
- Mutual attenuation with RG

Residual glycaemic:

- Statistical evidence of heterogeneity

- Mutual attenuation with RG

Beta cell +PI:

- Statistical evidence of heterogeneity
- Mutual attenuation with RG

Cataracts

All:

- No longer significant after adjusting for BMI and WHR

Obesity:

- T2D attenuates the effect of BMI and WHR

PCOS

All:

- Different directions between MR methods: Steiger IVW, MR-Egger, weighted mode
- Statistical evidence of horizontal pleiotropy
- No longer significant after adjusting for BMI and WHR
- T2D attenuates the effect of SAT volume

Obesity:

- Different directions between MR methods: MR-Egger
- Statistical evidence of horizontal pleiotropy
- No longer significant after adjusting for BMI and WHR
- T2D attenuates the effect of SAT volume

Erectile dysfunction

All:

- T2D attenuates the effect of BMI
- Mutual attenuation with WHR

Beta cell +PI:

- Mutual attenuation with BMI and WHR

Residual glycaemic:

- Mutual attenuation with BMI and WHR

Vascular dementia

All:

- T2D attenuates the effect of HDL

Obesity:

- No longer significant after adjusting for HDL

Beta cell +PI:

- No longer significant after adjusting for HDL

Asthma

Individuals genetically similar to Europeans:

- All:
    - Different directions between MR methods: MR-Egger, penalized weighted median, weighted median, weighted mode
    - Statistical evidence of horizontal pleiotropy
    - Statistical evidence of heterogeneity
    - No longer significant after adjusting for BMI and WHR
    - T2D attenuates the effect of VAT volume
- Residual glycaemic:
    - Different directions between MR methods: MR-Egger, weighted mode
    - Statistical evidence of horizontal pleiotropy
    - Statistical evidence of heterogeneity

    - No longer significant after adjusting for BMI
    - T2D attenuates the effect of VAT volume
- Obesity:
    - Statistical evidence of heterogeneity
    - No longer significant after adjusting for BMI
    - T2D attenuates the effect of VAT volume

Individuals genetically similar to East Asians:

- All:
    - TG effect changes direction to positive after adjustment for T2D effect, but it remains significant
- Residual glycaemic:
    - No longer significant after adjusting for TG levels
    - TG effect changes direction to positive after adjustment for T2D
- Beta cell +PI:
    - Statistical evidence of heterogeneity
    - TG effect changes direction to positive after adjustment for T2D, but it remains significant

Meta-analysis across genetic similarity groups:

- Statistical evidence of heterogeneity

COPD

Individuals genetically similar to Europeans:

- All:
    - Different directions between MR methods: Steiger IVW, MR-Egger, penalized weighted median, weighted median, weighted mode
    - Statistical evidence of horizontal pleiotropy
    - Statistical evidence of heterogeneity
    - T2D attenuates the effect of GFAT volume
    - No longer significant after adjusting for WHR
- Body fat:
    - Different directions between MR methods: Steiger IVW, MR-Egger, weighted mode
    - T2D attenuates the effect of GFAT volume
    - No longer significant after adjusting for BMI
- Obesity:
    - Statistical evidence of horizontal pleiotropy
    - Statistical evidence of heterogeneity
    - T2D attenuates the effect of GFAT volume
    - No longer significant after adjusting for BMI
- Beta cell +PI:
    - No longer significant after adjusting for BMI and WHR
    - Mutual attenuation with GFAT volume

Depression

Individuals genetically similar to Europeans:

- Body fat:
    - Different directions between MR methods: MR-Egger
    - Mutual attenuation with BMI
- Obesity:
    - Statistical evidence of heterogeneity

- No longer significant after adjusting for BMI

Supplementary Note 2: Detailed description of the results of the results of the multivariable Mendelian randomization analysis on the reverse statistically significant IVW results at an FDR of 5%.

Alzheimer's disease

The protective effect of Alzheimer's disease on T2D gets attenuated after adjustment for HDL levels, BMI or WHR.

Vascular dementia

Similarly to he protective effect of vascular dementia on T2D is no longer significant after adjustemnt for HDL levels, BMI or WHR.

Supplementary Note 3: MR-Clust identifies many clusters with opposite directions of effect.

As an additional comparison with our main analysis, we used the MR-Clust method, which groups together IVs with similar causal effect estimates on the outcome trait into distinct cluster[1]. In theory, IVs in these causal clusters may be involved in similar biological pathways, which allows one to identify unique pathways through which exposure has a causal effect on the outcome.

We tested T2D-comorbidity pairs with evidence of a causal relationship from the main analysis in MR-Clust. For each relationship analyzed with the MR-Clust algorithm, we used all 1,289 T2D IVs[2]. MR-Clust clusters were considered causal if they had at least 4 IVs with an 80% or greater probability of being included in the cluster. "Junk" and "Null" clusters identified by MR-Clust were ignored, and any IVs with less than 80% probability of cluster inclusion were removed from the cluster. Following the identification of causal clusters in each T2D-comorbidity pair, we used the IVW method to assess their direction of effect and significance (FDR adjusted p-value < 0.05). The cardiometabolic cluster information for each IV was then merged with each causal cluster to see if there were consistent relationships between an IV's cardiometabolic cluster and MR-Clust cluster.

We compared our biologically informed results with a statistical approach based on the similarity of the causal estimates implemented in the MR-Clust method[1]. MR-Clust identifies at least one causal cluster for 11 out of 15 comorbidities (73.3%) causally affected by T2D genetic risk (Supplementary Data 11, Supplementary Figure 54). For anorexia nervosa, osteoarthritis, ADHD, rheumatoid arthritis, and erectile dysfunction, MR-Clust and our results show concordant directions of effects. For instance, MR-Clust identifies a single cluster of T2D genetic predisposition causally associated with increased risk for anorexia nervosa consisting of 16 IVs (OR=0.23, q-value=1.08×10^{-36}). MR-Clust finds two causal clusters of T2D genetic predisposition causally associated with increased osteoarthritis risk, primarily composed of variants assigned to the obesity cluster, supporting our results (Supplementary Figure 56). However, many causal clusters for different comorbidities have a highly heterogeneous combination of variants contributing to their observed effect, such as ADHD's single causal cluster, which includes risk variants from all T2DGGI clusters (Supplementary Figures 57-71). A notable difference between both approaches is that MR-Clust often identifies cluster-stratified effects for one disease in opposite directions. In summary, we find consistent directions of causal estimates between our biologically informed approach and MR-Clust for five diseases. The MR-Clust clusters are, however, more complex to interpret biologically and tend to have opposing directions of effect for one comorbidity compared to our biologically informed clustering approach.

Supplementary References

1. Foley, C.N., et al., *MR-Clust: clustering of genetic variants in Mendelian randomization with similar causal estimates.* Bioinformatics, 2021. **37**(4): p. 531-541.
2. Suzuki, K., et al., *Genetic drivers of heterogeneity in type 2 diabetes pathophysiology.* Nature, 2024. **627**(8003): p. 347-357.
