# Supplementary material for: The effect of type 2 diabetes genetic predisposition on non-cardiovascular comorbidities
Source: Nat Commun. 2025 Oct 10;16:9042. doi: 10.1038/s41467-025-64927-5 (PMC12514310; doi:10.1038/s41467-025-64927-5)
Supplement: Supplementary file 2 — Description of Additional Supplementary Files [file 41467_2025_64927_MOESM2_ESM.docx]

# Description of files in Supplementary Information

## Supplementary Tables

**Supplementary Table 1**: Overview of results of previous two-sample Mendelian randomization studies investigating the causal effect of type 2 diabetes liability on comorbidity risk. (OR = odds ratio; lci_OR = lower bound of 95% confidence interval of OR; uci_OR = upper bound of 95% confidence interval of OR).

**Supplementary Table 2**: Sample size of each genetic similarity group type 2 diabetes GWAS meta-analysis from T2DGGI consortium.

**Supplementary Table 3**: Number of type 2 diabetes genetic instrumental variables (IVs) for each mechanistic cluster using different approaches to select IVs employed as sensitivity analysis for the Mendelian randomization analysis. (As per 1000 Genomes Project genetic ancestry groups: AFR = African, AMR = admixed American, EAS = East Asian, EUR = European, SAS = South Asian).

**Supplementary Table 4**: Sample size of each genetic similarity group from the All of Us genetic data used to perform the PheWAS. (As per 1000 Genomes Project genetic ancestry groups: AFR = African, AMR = admixed American, EAS = East Asian, EUR = European, SAS = South Asian).

**Supplementary Table 5**: Overview of the sensitivity analyses performed to assess the validity of the Mendelian randomization (MR) assumptions. * Definition of potential confounders/mediators: cardiometabolic traits used to cluster the type 2 diabetes (T2D) variants that have a potential causal IVW effect on the comorbidity at a false discovery rate (FDR) of 5%.

## Supplementary Figures

**Supplementary Figure 1:** Comparison of ours and previous studies results of two-sample Mendelian randomization (MR) analysis of genetic predisposition for type 2 diabetes (T2D) on non-cardiovascular comorbidity risk for the causal relationships. Causal estimates are expressed as the odds ratio (OR) for each comorbidity per doubling (2-fold increase) in genetically determined dichotomous T2D risk. (CI = confidence interval; CTS = Carpal tunnel syndrome; ADHD = attention-deficit/hyperactivity disorder; OCD = obsessive-compulsive disorder; PCOS = polycystic ovary syndrome).

**Supplementary Figure 2:** Results of the reverse Mendelian randomization (MR) analysis using genetic predisposition for type 2 diabetes (T2D) comorbidities as exposure and T2D as the outcome. Causal estimates are expressed as the odds ratio of comorbidity risk per doubling (2-fold increase) in genetically determined dichotomous T2D risk. Filled circles mark estimates with a false-discovery-rate correction of 5%. No estimate passed the MR sensitivity analyses. The genetic ancestry groups represent individuals genetically similar to Africans (AFR), East Asians (EAS), Europeans (EUR), admixed Americans (AMR) and South Asians (SAS) as defined by the 1000 Genomes Project. (CI = confidence interval).

**Supplementary Figure 3**: Results of cluster-stratified two-sample Mendelian randomization (MR) analysis of genetic predisposition for type 2 diabetes (T2D) on non-cardiovascular comorbidities risk. Causal estimates are expressed as the odds ratio (OR) of each comorbidity per doubling (2-fold increase) in genetically determined dichotomous T2D risk. Filled circles mark estimates with a q-value < 0.05 that passed all sensitivity analyses to assess the validity of the MR assumptions. (T2DGGI = Type 2 Diabetes Global Genomics Initiative; CI = confidence interval; PI = proinsulin; CTS = Carpal tunnel syndrome; ADHD = attention-deficit/hyperactivity disorder; OCD = obsessive-compulsive disorder; PCOS = polycystic ovary syndrome; PCOS = polycystic ovary syndrome; COPD = chronic obstructive pulmonary disease)

**Supplementary Figure 4:** Univariable Mendelian randomization (MR) results with genetic predisposition for cardiometabolic traits as exposures and type 2 diabetes (T2D) comorbidities as outcomes. Causal estimates are expressed as the odds ratio of comorbidity risk per doubling (2-fold increase) in genetically determined dichotomous T2D risk. Filled circles denote robust causal estimates that passed sensitivity analysis and false-discovery rate correction at 5%. (CI = confidence interval; PI = proinsulin; CTS = Carpal tunnel syndrome; ADHD = attention-deficit/hyperactivity disorder; OCD = obsessive-compulsive disorder; PCOS = polycystic ovary syndrome; COPD = chronic obstructive pulmonary disease; HDL=high-density lipoprotein cholesterol; WHR = waist-to-hip ratio; BMI=body mass index).

**Supplementary Figures 5-28**: Forest plots comparing the results of the univariable and multivariable Mendelian randomization (MR) analyses using data from individuals genetically similar to Europeans. Causal estimates are expressed as the odds ratio of comorbidity risk per doubling (2-fold increase) in genetically determined dichotomous T2D risk. (HDL=high-density lipoprotein cholesterol; WHR = waist-to-hip ratio; BMI=body mass index; CI=confidence interval; FDR=false-discovery-rate at 5%).

**Supplementary Figure 29**: Results of the single-ancestry cluster-stratified two-sample Mendelian randomization (MR) analysis of genetic predisposition for type 2 diabetes (T2D) on non-cardiovascular comorbidities risk. Causal estimates are expressed as the odds ratio (OR) of each comorbidity per doubling (2-fold increase) in genetically determined dichotomous T2D risk. Filled circles mark estimates with a q-value < 0.05 that passed all sensitivity analyses to assess the validity of the MR assumptions. (T2DGGI = Type 2 Diabetes Global Genomics Initiative; CI = confidence interval; PI = proinsulin; COPD = chronic obstructive pulmonary disease)

**Supplementary Figures 30-55:** Comparison between the results of Mendelian randomization (MR) results using the inverse variance weighted method and different approaches to select genetic instrumental variables (IVs). The forest plots depict all cluster-stratified estimates of genetic predisposition for type 2 diabetes (T2D) on comorbidity risk. Causal estimates are expressed as the odds ratio of comorbidity risk per doubling (2-fold increase) in genetically determined dichotomous T2D risk. Filled circles mark estimates with FDR < 5%. The genetic ancestry groups represent individuals genetically similar to Africans (AFR), East Asians (EAS), Europeans (EUR), admixed Americans (AMR) and South Asians (SAS) as defined by the 1000 Genomes Project. (CI = confidence interval).

**Supplementary Figure 56**: MR-Clust results for the es-mated cluster-stratified MR significant after FDR correction. Each estimate corresponds to one cluster identified by MR-Clust.

**Supplementary Figures 57-71**: MR-Clust scatterplots of putative T2D causal clusters on non-cardiometabolic comorbidities. MR-Clust (V.0.1.0) was used to identify putatively causal clusters of genetic predisposition to T2D as an exposure on non-cardiometabolic comorbidities as an outcome. We restricted putative causal clusters to instrumental variables (IVs) with a >80% probability of inclusion in the cluster, and removed any IVs placed in “Null” or “Junk” clusters by the MR-Clust algorithm. The genetic ancestry groups represent individuals genetically similar to Africans (AFR), East Asians (EAS), Europeans (EUR), admixed Americans (AMR) and South Asians (SAS) as defined by the 1000 Genomes Project. All scatterplots were generated using the “two_stage_plot” function implemented in MR-Clust. When possible, T2D IVs were annotated in the scatterplot by their cardiometabolic cluster from Suzuki et al. [2].

## Supplementary Notes

**Supplementary Note 1**: Detailed description of the results of the sensitivity analysis to assess the validity of the forward Mendelian randomization assumptions for the statistically significant IVW results at an FDR of 5%.

**Supplementary Note 2**: Detailed description of the results of the results of the multivariable Mendelian randomization analysis on the reverse statistically significant IVW results at an FDR of 5%.

**Supplementary Note 3**: MR-Clust identifies many clusters with opposite directions of effect.
