# Supplementary material for: The effect of type 2 diabetes genetic predisposition on non-cardiovascular comorbidities
Source: Nat Commun. 2025 Oct 10;16:9042. doi: 10.1038/s41467-025-64927-5 (PMC12514310; doi:10.1038/s41467-025-64927-5)
Supplement: Supplementary file 14 — Reporting Summary [file 41467_2025_64927_MOESM14_ESM.pdf]

nature portfolio

Corresponding author(s): Ana Luiza Arruda, Eleftheria Zeggini

Last updated by author(s): 27/09/2025

Reporting Summary

Nature Portfolio wishes to improve the reproducibility of the work that we publish. This form provides structure for consistency and transparency in reporting. For further information on Nature Portfolio policies, see our Editorial Policies and the Editorial Policy Checklist.

Statistics

For all statistical analyses, confirm that the following items are present in the figure legend, table legend, main text, or Methods section.

n/a	Confirmed	
☐	☒	The exact sample size (*n*) for each experimental group/condition, given as a discrete number and unit of measurement
☐	☒	A statement on whether measurements were taken from distinct samples or whether the same sample was measured repeatedly
☐	☒	The statistical test(s) used AND whether they are one- or two-sided<br>*Only common tests should be described solely by name; describe more complex techniques in the Methods section.*
☐	☒	A description of all covariates tested
☐	☒	A description of any assumptions or corrections, such as tests of normality and adjustment for multiple comparisons
☐	☒	A full description of the statistical parameters including central tendency (e.g. means) or other basic estimates (e.g. regression coefficient) AND variation (e.g. standard deviation) or associated estimates of uncertainty (e.g. confidence intervals)
☐	☒	For null hypothesis testing, the test statistic (e.g. *F*, *t*, *r*) with confidence intervals, effect sizes, degrees of freedom and *P* value noted<br>*Give P values as exact values whenever suitable.*
☒	☐	For Bayesian analysis, information on the choice of priors and Markov chain Monte Carlo settings
☐	☒	For hierarchical and complex designs, identification of the appropriate level for tests and full reporting of outcomes
☒	☐	Estimates of effect sizes (e.g. Cohen's *d*, Pearson's *r*), indicating how they were calculated

Our web collection on statistics for biologists contains articles on many of the points above.

Software and code

Policy information about availability of computer code

Data collection	No data was collected
Data analysis	The code used to perform all the MR-related and PheWAS analyses is publicly available and archived on Zenodo: https://doi.org/10.5281/zenodo.15168490.<br>LDlinkR R package (v1.3.0) https://doi.org/10.32614/CRAN.package.LDlinkR<br>TwoSampleMR R package (v0.5.7) https://mrcieu.github.io/TwoSampleMR/index.html<br>PLINK (v2) https://www.cog-genomics.org/plink/2.0/<br>MR-PRESSO https://doi.org/10.1038/s41588-018-0099-7<br>MendelianRandomization R package (v0.10) https://doi.org/10.32614/CRAN.package.MendelianRandomization<br>metafor R package (v4.6) https://doi.org/10.32614/CRAN.package.metafor<br>MVMR R package (v0.4) https://wspiller.github.io/MVMR/<br>smartpca (v7.2.1) https://christianhuber.github.io/smartsnp<br>PheTK package (v0.2.1rc5) https://pypi.org/project/PheTK/<br>ieugwasr R function (v1.0.2) https://doi.org/10.32614/CRAN.package.ieugwasr

For manuscripts utilizing custom algorithms or software that are central to the research but not yet described in published literature, software must be made available to editors and reviewers. We strongly encourage code deposition in a community repository (e.g. GitHub). See the Nature Portfolio guidelines for submitting code & software for further information.

Data

Policy information about availability of data

All manuscripts must include a data availability statement. This statement should provide the following information, where applicable:

- Accession codes, unique identifiers, or web links for publicly available datasets
- A description of any restrictions on data availability
- For clinical datasets or third party data, please ensure that the statement adheres to our policy

Researchers can apply to access the individual-level data of the All of Us Research Program (https://researchallofus.org/) used to perform the PheWAS. The publicly available GWAS summary statistics used in this work are referenced below and in Supplementary Data 8.
Alzheimer's disease: GWAS data has been deposited in The National Institute on Aging Genetics of Alzheimer's Disease Data Storage Site (NIAGADS)—a NIA/NIH-sanctioned qualified-access data repository, under accession NG00075.
Anorexia: https://pgc.unc.edu/for-researchers/download-results/
Asthma: https://www.globalbiobankmeta.org/resources
ADHD: https://pgc.unc.edu/for-researchers/download-results/
Austism: https://pgc.unc.edu/for-researchers/download-results/
Back pain: the dataset can also be accessed under 'Chronic back pain' from https://gwasarchive.org
Bipolar disorder: https://pgc.unc.edu/for-researchers/download-results/
CTS: https://www.decode.com/summarydata/
Cataracts: https://www.ebi.ac.uk/gwas/studies/GCST90014268
COPD: https://www.globalbiobankmeta.org/resources
Depression EUR: https://pgc.unc.edu/for-researchers/download-results/
Depression non-EUR: https://pgc.unc.edu/for-researchers/download-results/
Epilepsy: https://www.ebi.ac.uk/gwas/studies/GCST90271608
Erectile dysfunction: http://www.geenivaramu.ee/tools/ED_AJHG_Bovijn_et_al_2018.gz
Glaucoma: https://xikunhan.github.io/site/publication/
OCD: https://pgc.unc.edu/for-researchers/download-results/
Osteoarthritis: https://msk.hugeamp.org/downloads.html
Osteoporosis: https://pheweb.jp/
PCOS: https://doi.org/10.17863/CAM.27720
Rheumatoid arthritis: https://www.ebi.ac.uk/gwas/studies/GCST90132223
Schizophrenia: https://pgc.unc.edu/for-researchers/download-results/

Research involving human participants, their data, or biological material

Policy information about studies with human participants or human data. See also policy information about sex, gender (identity/presentation), and sexual orientation and race, ethnicity and racism.

Reporting on sex and gender	We did not collect any data, instead we use publicly available genome-wide association study (GWAS) summary statistics. The data we used was not stratified by sex nor gender. Except for sex-specific diseases like erectile dysfunction or polycystic ovary syndrome.
Reporting on race, ethnicity, or other socially relevant groupings	We did not collect any data, instead we use publicly available genome-wide association study (GWAS) summary statistics. If GWAS data from different genetic similarity groups were available, we performed analysis within genetic ancestry group and meta-analyzed the results.
Population characteristics	We did not collect any data, instead we use publicly available genome-wide association study (GWAS) summary statistics. We described the data in summary level, in Table 1 the more detailed description can be found in the respective GWAS publications.
Recruitment	We did not collect any data, instead we use publicly available genome-wide association study (GWAS) summary statistics.
Ethics oversight	We did not collect any data, instead we use publicly available genome-wide association study (GWAS) summary statistics.

Note that full information on the approval of the study protocol must also be provided in the manuscript.

Field-specific reporting

Please select the one below that is the best fit for your research. If you are not sure, read the appropriate sections before making your selection.

[x] Life sciences [ ] Behavioural & social sciences [ ] Ecological, evolutionary & environmental sciences

For a reference copy of the document with all sections, see nature.com/documents/nr-reporting-summary-flat.pdf

Life sciences study design

All studies must disclose on these points even when the disclosure is negative.

Sample size	No formal sample size calculation was performed for this study. We used the maximum available sample sizes from the respective genome-wide association studies (GWAS) for both exposures and outcomes. These GWAS were conducted in large, well-powered cohorts, with sample sizes ranging from, which ensures adequate statistical power to detect modest genetic associations. Leveraging these large-scale summary statistics is standard practice in Mendelian randomization studies and provides sufficient sample sizes to obtain reliable causal effect estimates.
Data exclusions	We did not exclude any data given that we used summary-level data only.
Replication	We replicated or findings using a PheWAS approach in the independent data of the All of Us research program. This cohort was not included in any of the GWAS summary statistics used in our MR analysis.
Randomization	In two-sample Mendelian Randomization, covariates are not directly included in the causal inference model. Instead, the approach relies on genetic variants as instrumental variables, which are randomly allocated at conception and thus independent of most environmental and lifestyle confounders. The genome-wide association studies (GWAS) from which the summary statistics were obtained had already adjusted their association estimates for key covariates such as age, sex, and principal components of ancestry to control for population stratification. Therefore, no additional covariate adjustment was performed within our MR analyses.
Blinding	This study is based on summary-level data from previously published genome-wide association studies (GWAS) and does not involve new data collection or allocation of participants into experimental groups. Therefore, blinding of investigators to group allocation was not applicable.

Reporting for specific materials, systems and methods

We require information from authors about some types of materials, experimental systems and methods used in many studies. Here, indicate whether each material, system or method listed is relevant to your study. If you are not sure if a list item applies to your research, read the appropriate section before selecting a response.

Materials & experimental systems

n/a	Involved in the study	
☒	☐	Antibodies
☒	☐	Eukaryotic cell lines
☒	☐	Palaeontology and archaeology
☒	☐	Animals and other organisms
☒	☐	Clinical data
☒	☐	Dual use research of concern
☒	☐	Plants

Methods

n/a	Involved in the study	
☒	☐	ChIP-seq
☒	☐	Flow cytometry
☒	☐	MRI-based neuroimaging

Plants

Seed stocks	*Report on the source of all seed stocks or other plant material used. If applicable, state the seed stock centre and catalogue number. If plant specimens were collected from the field, describe the collection location, date and sampling procedures.*
Novel plant genotypes	*Describe the methods by which all novel plant genotypes were produced. This includes those generated by transgenic approaches, gene editing, chemical/radiation-based mutagenesis and hybridization. For transgenic lines, describe the transformation method, the number of independent lines analyzed and the generation upon which experiments were performed. For gene-edited lines, describe the editor used, the endogenous sequence targeted for editing, the targeting guide RNA sequence (if applicable) and how the editor was applied.*
Authentication	*Describe any authentication procedures for each seed stock used or novel genotype generated. Describe any experiments used to assess the effect of a mutation and, where applicable, how potential secondary effects (e.g. second site T-DNA insertions, mosiacism, off-target gene editing) were examined.*
